# 5-Aminothiazoles Reveal a New Ligand-Binding Site on Prolyl Oligopeptidase Which is Important for Modulation of Its Protein–Protein Interaction-Derived Functions

**DOI:** 10.1021/acs.jmedchem.3c01993

**Published:** 2024-03-28

**Authors:** Henri T. Pätsi, Tommi P. Kilpeläinen, Mikael Jumppanen, Johanna Uhari-Väänänen, Pieter Van Wielendaele, Francesca De Lorenzo, Hengjing Cui, Samuli Auno, Janne Saharinen, Erin Seppälä, Nina Sipari, Juha Savinainen, Ingrid De Meester, Anne-Marie Lambeir, Maija Lahtela-Kakkonen, Timo T. Myöhänen, Erik A. A. Wallén

**Affiliations:** † Drug Research Program, Division of Pharmaceutical Chemistry and Technology, Faculty of Pharmacy, University of Helsinki, P.O. Box 56, 00014 Helsinki, Finland; ‡ Drug Research Program, Division of Pharmacology and Pharmacotherapy, Faculty of Pharmacy, University of Helsinki, P.O. Box 56, 00014 Helsinki, Finland; § Laboratory of Medical Biochemistry, Department of Pharmaceutical Sciences, Faculty of Pharmaceutical, Biomedical and Veterinary Sciences, University of Antwerp, 2610 Wilrijk, Belgium; ∥ School of Pharmacy, Faculty of Health Sciences, University of Eastern Finland, Yliopistonranta 1C, 70211 Kuopio, Finland; ⊥ School of Medicine/Biomedicine, Faculty of Health Sciences, University of Eastern Finland, Yliopistonranta 8, Kuopio 70211, Finland; # Viikki Metabolomics Unit, Faculty of Biological and Environmental Sciences, University of Helsinki, Viikinkaari 5 E, 00014 Helsinki, Finland; ¶ Division of Pharmacology, Faculty of Medicine, University of Helsinki, P.O.Box 63, 00014 Helsinki, Finland

## Abstract

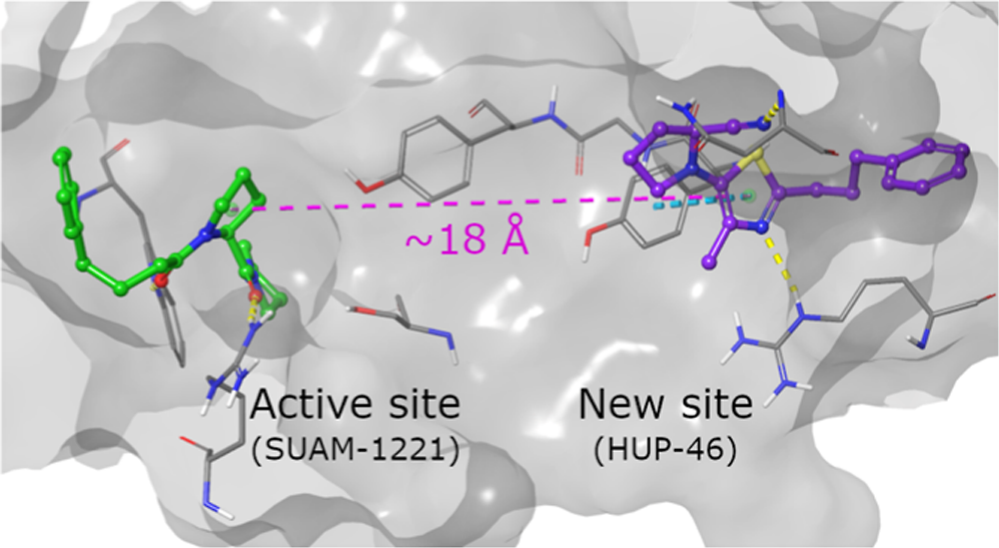

A series of novel 5-aminothiazole-based ligands for prolyl oligopeptidase (PREP) comprise selective, potent modulators of the protein–protein interaction (PPI)-mediated functions of PREP, although they are only weak inhibitors of the proteolytic activity of PREP. The disconnected structure–activity relationships are significantly more pronounced for the 5-aminothiazole-based ligands than for the earlier published 5-aminooxazole-based ligands. Furthermore, the stability of the 5-aminothiazole scaffold allowed exploration of wider substitution patterns than that was possible with the 5-aminooxazole scaffold. The intriguing structure–activity relationships for the modulation of the proteolytic activity and PPI-derived functions of PREP were elaborated by presenting a new binding site for PPI modulating PREP ligands, which was initially discovered using molecular modeling and later confirmed through point mutation studies. Our results suggest that this new binding site on PREP is clearly more important than the active site of PREP for the modulation of its PPI-mediated functions.

## Introduction

**Figure 1:**
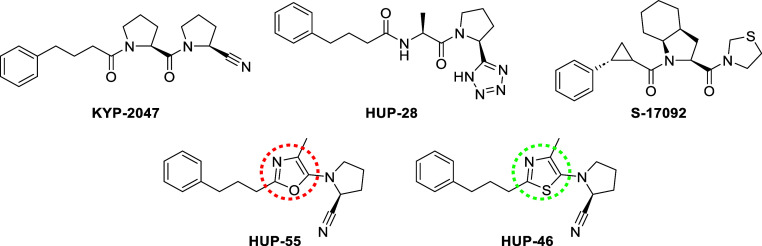
Peptide-like PREP inhibitors KYP-2047 and HUP-28, both of which are also strong modulators of the PPI-mediated functions of PREP. S-17092 is a peptide-like potent PREP inhibitor lacking an effect on multiple PPI-mediated functions of PREP. The structurally distinct nonpeptidic 5-aminooxazole-based PREP inhibitor HUP-55 and its new 5-aminothiazole-based analogue **HUP-46** presented in this paper.

Prolyl oligopeptidase (PREP; EC 3.4.21.26) is a proline-specific serine-type endopeptidase found in most tissues in the human body, especially in the brain, and also in high amounts in liver, testis, and ovary.^1^ Although it is a proteolytic enzyme, it also regulates many important processes in the human body via direct interactions with other proteins such as alpha-synuclein (αSyn), Tau, and protein phosphatase 2A (PP2A).^2−4^ These interactions result in increased αSyn and Tau aggregation, decreased PP2A activity and autophagy, and increased reactive oxygen species (ROS) production.^5^ Abnormal processing and aggregation of αSyn and Tau are thought to be the key players in cellular toxicity in Parkinson’s disease (PD) and Alzheimer’s disease (AD), respectively,^6,7^ and decreased PP2A levels and activity are detected in various neurodegenerative diseases (NDDs) such as PD, AD, and other tauopathies.^8,9^ Therefore, targeting the protein–protein interactions (PPIs) of PREP could be a viable therapeutic strategy for several NDDs.^10^ PREP inhibitors that can modulate the PPI-mediated functions of PREP, such as KYP-2047 (Figure 1), reduce αSyn and Tau aggregation, increase the clearance of αSyn and Tau aggregates via enhanced autophagy, and decrease ROS production in both in vitro and in vivo models of PD and AD.^4,5,11−19^ Originally it was believed that PREP inhibition, i.e., loss of conformational freedom of the enzyme upon inhibitor binding to the proteolytic active site, was responsible for modulating these PPIs. However, it is now evident that the structure–activity relationships (SARs) for the inhibition of its proteolytic activity and regulation of the PPI-derived functions are at least to some extent disconnected.^16−19^ Contrary to KYP-2047, another well-known potent PREP inhibitor S-17092, which is also the most recent PREP inhibitor that entered clinical trials,^20^ does not affect multiple PPI-derived functions of PREP in our assays.^17^ Furthermore, several weak inhibitors are significantly more effective modulators of the PPI-derived functions than KYP-2047.

In our efforts to clarify why PREP inhibition does not correlate with the effects of PPI-derived functions, we challenged the known SAR for PREP ligands. Typical PREP inhibitors are based on a peptide-like scaffold and contain two amide carbonyl groups that are essential for binding to the proteolytic active site (Figure 1).^21^ Currently, the most structurally distinct PREP inhibitors are our recently published 5-aminooxazoles, which lack both carbonyl groups.^18^ Although the 5-aminooxazole series contained several potent compounds, both in terms of inhibiting the proteolytic activity of PREP and modulating its PPI-mediated functions, the varying stability of 5-aminooxazoles restricted the full exploration of different amino groups in the 5-position and limited it to (*S*)-2-cyanopyrrolidine for the biologically active compounds. HUP-55 is the most potent compound in the oxazole series (Figure 1).

In this study the 5-membered heteroaromatic scaffold was further explored by replacing the oxazole ring with a thiazole ring (Figure 1). The 5-aminothiazoles are far more stable than the 5-aminooxazoles for the exploration of different substituents. The compounds were developed solely with a focus on their effect on αSyn dimerization and autophagy, which are the two most studied PPI-derived functions of PREP. We also set out to explain the disconnected SARs using molecular modeling supported by point mutation studies.

## Results and Discussion

### Synthesis

**Scheme 1:**
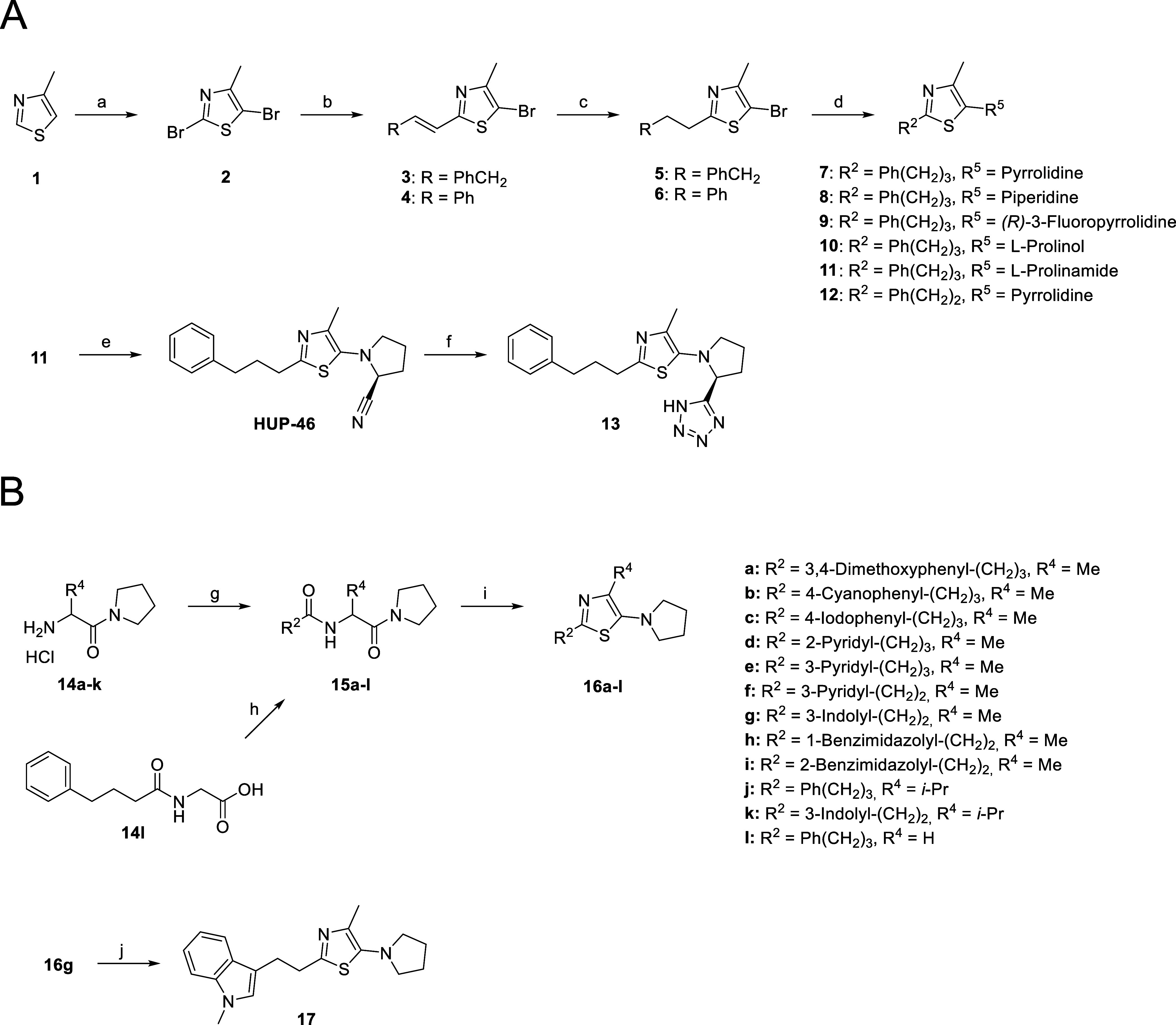
Synthesis of 5-Aminothiazoles Reagents and conditions: (a) CBr_4_, *t*BuONa/DMF, r.t., 1.5 h; (b) appropriate boronic acid, Pd(PPh_3_)_4_, Li_2_CO_3_/dioxane, H_2_O, 100 °C, 24 h; (c) *N*,*O*-bis(trifluoroacetyl)hydroxylamine, NH_2_OH/dioxane, 100 °C, 22 h; (d) appropriate amine, Cs_2_CO_3_/DMF, 180 °C, 30 min, or alternatively, appropriate amine, *t*BuONa, Rh(cod)_2_BF_4_, 1,3-diisopropylimidazolium chloride/dimethoxyethane, 80 °C, 19 h (compounds **7**, **8**, and **10**); (e) TFAA, Et_3_N/THF, 0 °C, 2 h; (f) ZnBr_2_, NaN_3_/H_2_O, reflux, 20 h; (g) R^2^CO_2_H, HBTU, DIPEA/DCM (or in DMF for compounds **15a**, **15e**, and **15g**), r.t., 16 h, or alternatively, R^2^COCl, Et_3_N/DCM, r.t., 19 h (compound **15c**); (h) (1) pivaloyl chloride, Et_3_N/DCM, 0 °C, 1 h and (2) pyrrolidine, Et_3_N/DCM, r.t., 3 h; (i) Lawesson’s reagent/pyridine, MW, 150 °C, 30 min; (j) NaH, MeI, DMF, r.t., 4 d.

The thiazoles were initially synthesized via route A of Scheme 1. This route was planned to access the thiazole analogues of HUP-55 with and without the nitrile group, and other analogues could also be synthesized via this route. In route A, 4-methylthiazole **1** was first brominated at the 2 and 5 positions, resulting in compound **2**.^22^ Compound **2** was then reacted via a Suzuki reaction with *trans*-3-phenyl-1-propen-1-ylboronic acid and *trans*-2-phenylvinylboronic acid, resulting in compounds **3** and **4**, respectively. This reaction was completely selective for bromine in the 2-position. Attempts to directly react the bromine in the 5-position resulted in a mixture of correct product and another product where the bromine had reacted but the amine was attached to the conjugated double bond in the 2-position. We therefore found it necessary to first reduce the alkene. Unfortunately, catalytic hydrogenation with Pd/C resulted in dehalogenation. However, when compounds **3** and **4** were reacted with the milder reducing reagent *N*,*O*-bis(trifluoroacetyl)hydroxylamine,^23^ we obtained compounds **5** and **6**, respectively, with a conversion of ca. 75% without any dehalogenation. The desired amine could then be introduced via a nucleophilic aromatic substitution reaction on the bromine to the 5-position of the thiazole ring. Finally, the amide of compound **11** was dehydrated to a nitrile using TFAA to obtain compound **HUP-46**, which was then reacted with NaN_3_ to obtain the corresponding tetrazole, compound **13**.^16^

An alternative route was chosen to synthesize a wider series of analogues (route B in Scheme 1). This route was similar to the primary route we used in the synthesis of 5-aminooxazoles.^18^ The central peptidic intermediates **15a**–**k** were synthesized in one step from suitable amino acid derivatives, such as amino acid pyrrolidides **14a**–**k** or *N*-(4-phenylbutanoyl)glycine **14l**. Lawesson’s reagent was used to form the thiazole ring and obtain the final 5-aminothiazole products **16a**–**l**.^24^ Finally, compound **16g** was methylated to compound **17**. Compounds **HUP-46** and **7** were later also synthesized using route B, resulting in better overall yields than that with route A.

### In Vitro and Cellular Screening Results of Synthesized Compounds

The thiazoles were tested for their ability (1) to inhibit the proteolytic activity of PREP using recombinant porcine PREP due to its similar structure to human PREP (hPREP) (97% similarity),^27^ (2) to block αSyn dimerization in a cellular protein fragment complementation assay (PCA), (3) to induce autophagy in a green fluorescent protein (GFP)-tagged microtubule-associated proteins’ 1A/1B light chain 3B (LC3B) expressing human embryonic kidney 293 (HEK-293) cell culture, and (4) to reduce ROS production in FeCl_2_- and H_2_O_2_-stressed SH-SY5Y cells, as previously described for 5-aminooxazoles.^18^ The results are presented in Table 1. In the autophagy assay, a decreased GFP signal compared to the DMSO control is indicative of increased autophagic flux. Rapamycin, a potent autophagy inducer, reduces the GFP signal to about 65% of the baseline (Supporting Information Figure S51), which can be considered the maximum effect a compound can have in this assay. Compounds that reduce the fluorescence signal in the αSyn dimerization assay and the GFP signal in the autophagy assay below 90% of the baseline can be considered active, as the in vivo effective reference compounds KYP-2047 and HUP-55^18^ reduce them to 87 and 89%, respectively.

**Table 1:**
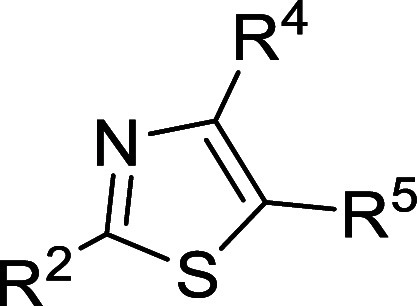
Biological Activities of the Synthesized 5-Aminothiazoles^i^

compound	R^2^	R^4^	R^5^	IC_50_ (nM)^a^ (95% CI)		αSyn dimerization at 10 μM (%)^b^	autophagy at 10 μM (%)^c^	ROS at 10 μM (%)^d^
KYP-2047^e^				<1^f^		87 ± 1	89 ± 3	88 ± 3
HUP-28^g^				130	(71–230)	75 ± 5	71 ± 6	95 ± 7
HUP-55^e^				5.0	(3.2–7.6)	85 ± 2	87 ± 3	85 ± 3
**HUP-46**	Ph(CH_2_)_3_–	Me	(*S*)-2-cyanopyrrolidin-1-yl	8000	(4900–13,000)	74 ± 2	77 ± 3	85 ± 11
**7**	Ph(CH_2_)_3_–	Me	pyrrolidin-1-yl	4800	(2100–11,000)	74 ± 2	87 ± 4	75 ± 9
**8**	Ph(CH_2_)_3_–	Me	piperidin-1-yl	210,000	(74,000–590,000)	82 ± 11	106 ± 10	73 ± 26
**9**	Ph(CH_2_)_3_–	Me	(*R*)-3-fluoropyrrolidin-1-yl	97,000	(25,000–380,000)	95 ± 1	95 ± 2	n.d.
**10**	Ph(CH_2_)_3_–	Me	(*S*)-2-hydroxymethylpyrrolidin-1-yl	95,000	(35,000–260,000)	99 ± 19	96 ± 3	n.d.
**11**^h^	Ph(CH_2_)_3_–	Me	(*S*)-2-carbamoylpyrrolidin-1-yl	80,000	(15,000–420,000)	94 ± 8	84 ± 3	86 ± 4
**12**	Ph(CH_2_)_2_–	Me	pyrrolidin-1-yl	47,000	(18,000–120,000)	107 ± 15	89 ± 2	59 ± 18
**13**	Ph(CH_2_)_3_–	Me	2-tetrazolylpyrrolidin-1-yl	n.d.	n.d.	111 ± 10	98 ± 5	86 ± 11
**16a**	3,4-dimethoxyphenyl–(CH_2_)_3_–	Me	pyrrolidin-1-yl	12,000	(4100–33,000)	92 ± 5	83 ± 6	48 ± 3
**16b**	4-cyanophenyl–(CH_2_)_3_–	Me	pyrrolidin-1-yl	340	(68–1700)	113 ± 9	96 ± 1	53 ± 4
**16c**	4-iodophenyl–(CH_2_)_3_–	Me	pyrrolidin-1-yl	9800	(2600–38,000)	n.d.	97 ± 3	n.d.
**16d**	2-pyridyl–(CH_2_)_3_–	Me	pyrrolidin-1-yl	7300	(4800–11,000)	96 ± 7	104 ± 4	57 ± 2
**16e**	3-pyridyl–(CH_2_)_3_–	Me	pyrrolidin-1-yl	7900	(5700–11,000)	105 ± 3	102 ± 5	60 ± 3
**16f**	3-pyridyl–(CH_2_)_2_–	Me	pyrrolidin-1-yl	3300	(2000–5300)	96 ± 8	108 ± 3	62 ± 5
**16g**	3-indolyl–(CH_2_)_2_–	Me	pyrrolidin-1-yl	5700	(3700–8600)	105 ± 3	83 ± 5	55 ± 3
**16h**	1-benzimidazolyl–(CH_2_)_2_–	Me	pyrrolidin-1-yl	62,400	(37,680–103,200)	86 ± 8	78 ± 5	n.d.
**16i**	2-benzimidazolyl–(CH_2_)_2_–	Me	pyrrolidin-1-yl	2100	(730–6300)	93 ± 2	101 ± 6	59 ± 3
**16j**	Ph(CH_2_)_3_–	isopropyl	pyrrolidin-1-yl	4000	(2600–6100)	90 ± 6	97 ± 3	85 ± 5
**16k**	3-indolyl–(CH_2_)_2_–	isopropyl	pyrrolidin-1-yl	5800	(12,000–79,000)	105 ± 8	100 ± 6	83 ± 4
**16l**	Ph(CH_2_)_3_–	H	pyrrolidin-1-yl	126,000	(49,350–340,500)	125 ± 21	87 ± 4	n.d.
**17**	*N*-methyl-3-indolyl–(CH_2_)_2_–	Me	pyrrolidin-1-yl	3900	(1900–7700)	89 ± 8	103 ± 3	n.d.

a Assessed using recombinant porcine PREP with Suc-Gly-Pro-AMC as the substrate.

b Luminescence signal percentage of DMSO control with SEM, assessed with a split *Gaussia* luciferase-based method using Neuro2A cells.

c GFP signal percentage of DMSO control with SEM, assessed using HEK-293 cells stably expressing GFP-LC3B.

d Fluorescence signal percentage of DMSO control with SEM, assessed using a fluorogenic ROS assay.

e Results reported by Kilpeläinen et al.^18^

f The assay is limited by the enzyme concentration of 2 nM for IC_50_ values under this concentration; KYP-2047 is a slow, tight-binding inhibitor with a *K*_*i*_-value of 0.02 nM.^25,26^

g Results except ROS assay reported by Kilpeläinen et al.^16^ or Pätsi et al.^19^

h Synthesis intermediate.

i n.d.: not determined.

A comparison of the inhibitory potencies of 5-aminooxazole HUP-55 (5 nM) and the corresponding 5-aminothiazole, **HUP-46** (8 μM), supports the postulated binding mode for HUP-55 at the active site, where the oxygen atom in the oxazole ring is involved in a hydrogen bond (Supporting Information Figure S42).^18^ Replacing the oxazole oxygen with sulfur in thiazoles removes this binding interaction, which could explain the decrease in inhibitory potency. This is a general characteristic for all thiazoles in our series, and none of them are nanomolar inhibitors of the proteolytic activity of PREP. Despite the weak inhibitory potency, **HUP-46** was an even more potent modulator of αSyn dimerization and autophagy than HUP-55 or KYP-2047. Compound **7** was equipotent to **HUP-46** in modulating αSyn dimerization and only slightly less active in modulating autophagy, although compound **7** lacks the nitrile group.

Compound **11** demonstrated that an amide group cannot be placed in the position of the nitrile group as it had no effect on αSyn dimerization; however, its effect on autophagy was similar to that of compound **7**. It should be noted that the purity of this synthesis intermediate was only determined by NMR. Other substituents on the pyrrolidine ring gave inactive compounds. Interestingly, increasing the size of the pyrrolidine ring to a piperidine ring in compound **8** removed the activity on autophagy but only slightly weakened the effect on αSyn dimerization compared to compound **7**.

Shortening the linker length in the 2-position of compound **7** by one carbon, resulting in compound **12**, removed the effect on αSyn dimerization but maintained the effect on autophagy. Compound **16a**, where the phenyl group was 3,4-dimethoxy-substituted, also maintained the effect on autophagy while losing the effect on αSyn dimerization. Other replacements in the 2-position of the thiazole ring typically resulted in a loss of effect in both assays. Compound **16g**, with 2-(3-indolyl)ethyl as the 2-substituent, had an effect on autophagy while lacking an effect on αSyn dimerization. Its N-methylated analogue, compound **17**, was inactive in both assays. Replacing the 3-indolyl group with a 1-benzimidazolyl group resulted in compound **16h**, which showed a comparable effect in both assays to **HUP-46** and compound **7**. However, the corresponding 2-benzimidazolyl analogue was inactive in both assays.

Replacing the methyl group in the 4-position of compounds **7** and **16g** with an isopropyl group, resulting in compounds **16j** and **16k**, respectively, also led to a loss of activity for both PPI-mediated functions. This is opposite to what we saw with oxazoles, where a small increase in the size of this substituent improved the activity. It should be noted that in the case of the corresponding oxazoles, the 5-substituent was always (*S*)-2-cyanopyrrolidine. Removing the methyl group in the 4-position resulted in compound **16l**, which had an effect on autophagy but not on αSyn dimerization.

Interestingly, several thiazoles were highly effective at reducing the ROS levels in cells with oxidative stress. Eteläinen et al.^5^ showed that KYP-2047 reduces ROS levels by reducing the activity of NADPH oxidase via PP2A activation. Therefore, autophagy and ROS reduction results should have a correlation, but it appears not to be the case with thiazoles. However, thiazoles have been reported to have antioxidant properties themselves,^28^ and this may explain the discrepancy between the autophagy induction and ROS production.

**HUP-46**, **7**, and **16h** were the most potent modulators of the PPI-mediated functions of PREP in our assays. **HUP-46** was the overall most effective compound, being equipotent to compound **7** in reducing αSyn dimerization and equipotent to compound **16h** in increasing autophagy, and it was therefore chosen for further testing in the cellular assays. Overall, **HUP-46** is one of the most potent modulators of the PPI-mediated functions of PREP that has so far been reported.

### Biological Characterization of **HUP-46**

**Figure 2:**
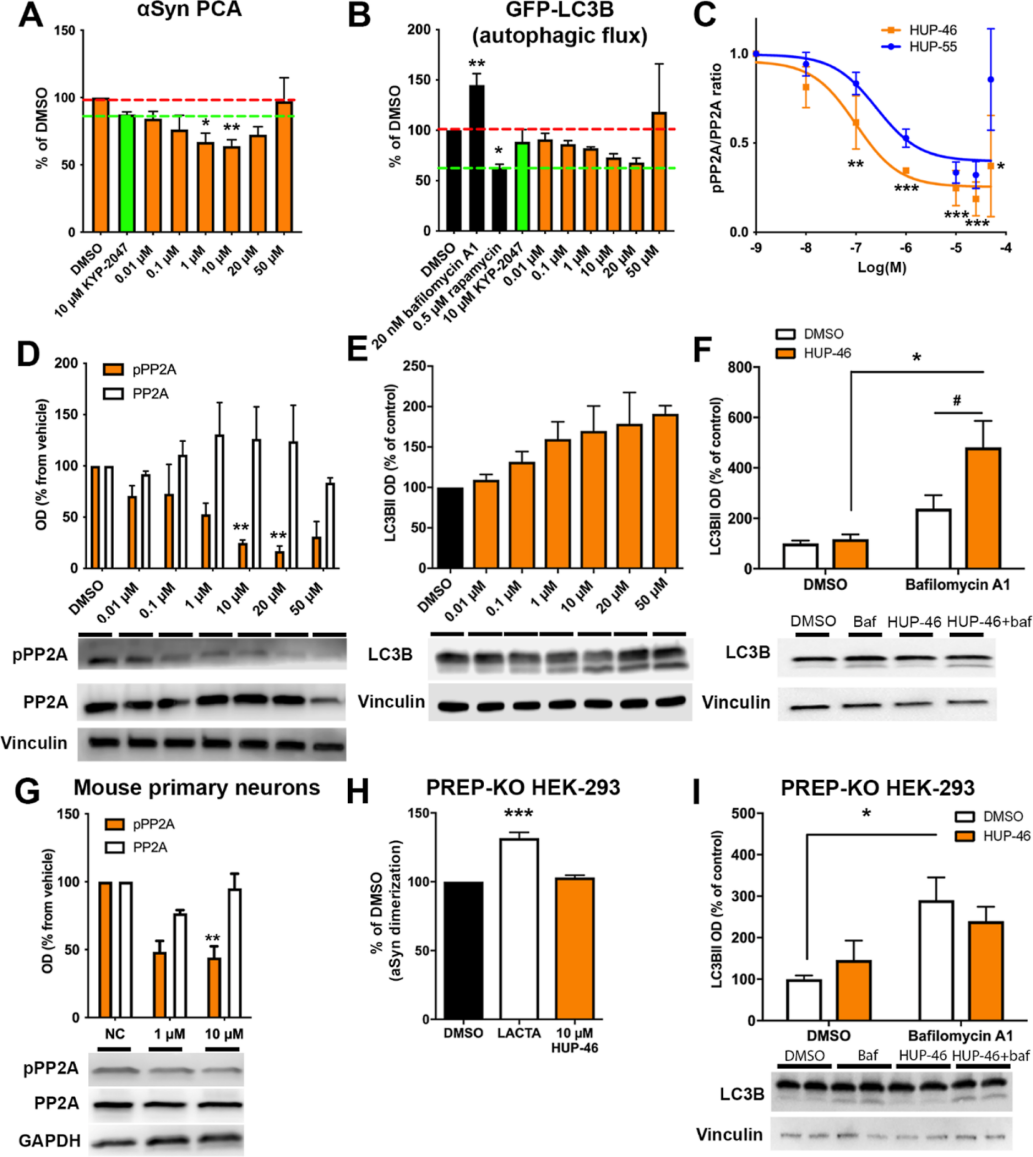
Effect of **HUP-46** on cellular PP2A and autophagy markers and specificity in PREP knockout (PREP-KO) cells. The concentration-dependent effect of **HUP-46** was tested in αSyn dimerization [(A); protein-fragment complementation assay (PCA)] and GFP-LC3B autophagic flux assays (B). 10 μM showed the maximal efficacy when considering both assays. Red line shows the DMSO control level, and green line shows the assay control level [KYP-2047 in (A); rapamycin in (B)]. The potency (EC_50_ value) for PP2A activation was determined from the inactive PP2Ac (pPP2A)/total PP2Ac ratio after 4 h incubation with **HUP-46** (C,D). The EC_50_ ratio for HUP-55 (blue line) is adapted from Kilpeläinen et al.^18^ The ratio was significantly decreased to 1 μM concentration. A dose-dependent effect was seen on LC3BII levels in HEK-293 cells after 4 h incubation (E). An autophagic flux assay showed significantly elevated LC3BII levels with 20 nM bafilomycin A1 and 10 μM **HUP-46** after 4 h incubation (F), confirming the autophagic flux activation. A significant effect on pPP2A was also seen in mouse primary neurons (G). In an αSyn PCA with 10 μM lactacystin (LACTA) (10 μM) as a positive control, no effect with 10 μM **HUP-46** was seen on αSyn dimerization in PREP-KO cells (H). Similarly, no effect with 10 μM **HUP-46** and 20 nM bafilomycin was seen on LC3BII levels in PREP-KO HEK-293 cells (I). Data are presented as mean ± SEM *, *p* < 0.05, **, *p* < 0.01; ***, *p* < 0.001; one-way ANOVA with Tukey’s posthoc test (A–D,G,H) and two-way ANOVA with Sidak’s posthoc test (F,I); *, #, *p* < 0.05, two-way ANOVA with Sidak’s posthoc test (F).

After the cellular screening assays, we wanted to assess the concentration–response effect of **HUP-46** on αSyn dimerization and autophagic flux in GFP-LC3B-RFP HEK-293 cells. In the αSyn dimerization assay, the maximal efficacy was achieved at 10 μM concentration, and, similar to HUP-55,^18^ higher concentrations did not show an additional effect (Figure 2A; *p* < 0.01 10 μM; *p* < 0.05 1 μM **HUP-46** compared to DMSO, one-way ANOVA with Tukey’s post hoc test). In the autophagic flux assay, the effect was concentration-dependent up to 20 μM, but 50 μM restored the autophagic flux to control levels (Figure 2B). This might be indicative for toxicity-induced changes in autophagic flux, as 100 μM concentration showed toxicity in neuronal and non-neuronal cells (Supporting Information Figure S52). Moreover, the restored autophagic flux may also contribute to the elevated signal in αSyn dimerization assay with 50 μM concentration (Figure 2A). PP2A activation and autophagy were then measured in nonreporter cells. A 4 h incubation in HEK-293 cells increased the levels of the total catalytic subunit of PP2A (PP2Ac; Figure 2C,D) and significantly decreased the levels of inactive PP2Ac (pPP2Ac; specificity for inactive PP2A tested by Svarcbahs et al.^3^ and Eteläinen et al.;^4^Figure 2C,D; *p* < 0.01, 10 and 20 μM **HUP-46** compared to NC, one-way ANOVA with Tukey’s post hoc test) at 10 and 20 μM concentrations. Importantly, the ratio of pPP2A/PP2A was already decreased at 0.01 μM concentration and above, indicating PP2A activation (Figure 2C; *p* < 0.05, 0.01 and 50 μM **HUP-46** compared to NC; *p* < 0.01, 0.1 μM **HUP-46** compared to NC; *p* < 0.001, 10, and 20 μM **HUP-46** compared to NC; one-way ANOVA with Tukey’s post hoc test). Based on the pPP2A/PP2A ratio, an EC_50_ value of 100 nM was calculated for **HUP-46** (Figure 2C), whereas for HUP-55 from the oxazole series, the EC_50_ value was 275 nM.^18^ In addition to potency, maximal efficacy on the pPP2A/PP2A ratio was better for **HUP-46** than for HUP-55 (Figure 2C; 0.18 vs 0.32).

In line with the results in autophagy reporter cells, LC3BII levels also increased concentration-dependently with **HUP-46** (Figure 2E). The results obtained with the GFP-LC3B-RFP cell culture were confirmed by using an autophagic flux assay where HEK-293 cells were incubated with 20 nM bafilomycin A1, which blocks the fusion between autophagosome and lysosome, combined with DMSO vehicle or 10 μM **HUP-46** for 4 h. The combination of bafilomycin A1 and **HUP-46** caused a significant increase in LC3BII levels compared to bafilomycin A1 alone, confirming that **HUP-46** induced autophagy (Figure 2F; *p* < 0.001 DMSO + **HUP-46** vs bafilomycin A1 + **HUP-46**; *p* < 0.05 bafilomycin A1 + DMSO vs bafilomycin A1 + **HUP-46**; two-way ANOVA with Sidak’s multiple comparison test).

**HUP-46** was also tested in mouse primary neurons, where it significantly decreased pPP2A levels with both 1 and 10 μM concentrations (Figure 2G; *p* < 0.01 compared to NC, one-way ANOVA with Tukey’s post-test). The impact of **HUP-46** on αSyn dimerization and autophagy is PREP-specific as there was no effect on αSyn dimerization (Figure 2H) or autophagic flux (Figure 2I) in PREP knockout (PREP-KO) HEK-293 cells. Moreover, no effect on the activities of closely related proteases [fibroblast activating protein (FAP), dipeptidyl peptidase (DPP) 2, 4 and 9] was seen with **HUP-46** (Supporting Information Figure S53).

As these compounds are being developed to target PREP in the brain, **HUP-46** was also shown to penetrate the blood–brain barrier by measuring its brain concentration in mice after i.p. injection (Supporting Information Figure S61).

### Identifying a Possible Binding Site Using Molecular Modeling

So far, we have been unable to give an explanation for the disconnected SARs for the inhibition of the proteolytic activity and modulation of the PPI-mediated effects of PREP. A previous study using molecular dynamics (MD) simulations identified some subtle differences in PREP dynamics when three different ligands were bound to the active site, and the authors hypothesized that this could be the reason behind the differences in their ability to modulate the PPI between αSyn and PREP.^29^ Although the ligands chosen for that study differ in their efficacy in modulating αSyn aggregation and autophagy,^17^ they are all potent peptide-like inhibitors of PREP, and this hypothesis does not explain how very weak inhibitors can modulate the PPI-mediated functions.

Our hypothesis was that the most likely explanation is the presence of another binding site, separate enough from the active site so that ligand binding to this other site does not prevent substrate binding. Additionally, we hypothesized that there should be similarities in ligand binding at the other binding site and the catalytic binding site. This would allow ligands like KYP-2047, which in addition to being modulators of the PPI-mediated functions are potent inhibitors, to bind to both the active site and this other site, with their affinity to each site determining their activity profile.

**Figure 3:**
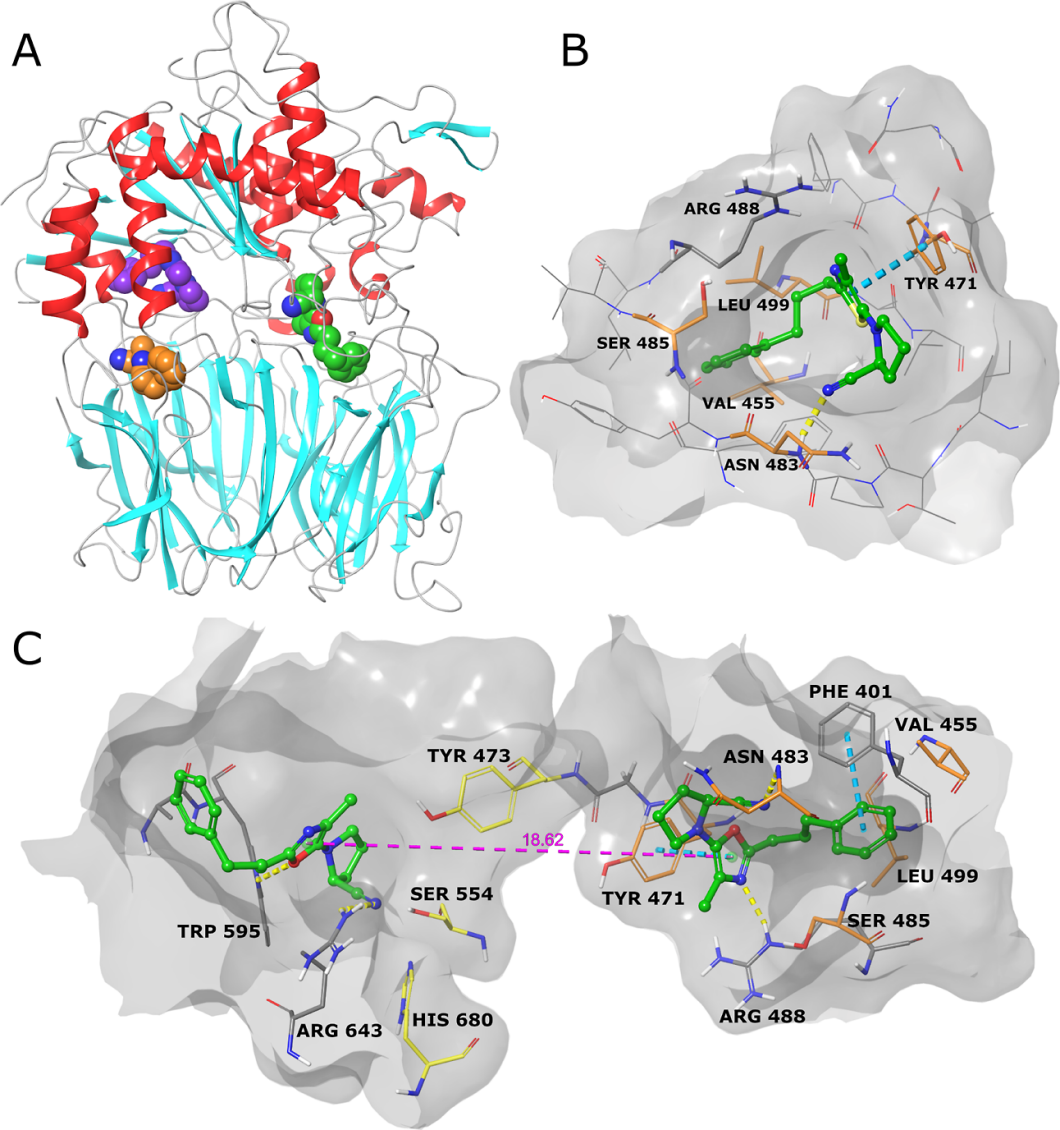
(A) Ribbon representation of the crystal structure of hPREP (PDB ID: 3DDU)^30^, with 5-aminooxazole HUP-55 docked into the active site (green) and two other possible binding sites (purple and orange). (B) Induced fit docking pose for **HUP-46** at the postulated new binding site. (C) Induced fit docking poses of HUP-55 at both the active site and the postulated new binding site, with the measured distance between the oxazole rings of 18.62 Å. The amino acid residues chosen for mutation studies are colored orange. Hydrogen bonds are shown as yellow dashed lines and π–π stacking as blue dashed lines.

Molecular modeling was used to identify possible binding sites. These studies were performed with the only available crystal structure of hPREP (PDB ID: 3DDU).^30^ Using Maestro’s SiteMap tool,^31^ two sites inside the cavity of PREP, near the hinges connecting the two subunits, were identified as potential new binding sites (orange and purple in Figure 3A). They both had a SiteScore of 1.0, which is equal to that of the active site. The 5-aminothiazoles in Table 1, the previously published 5-aminooxazoles,^18^ and selected peptide-like ligands^16,19^ were docked to the two binding sites using two methods: Glide and Induced Fit.^31^ Based on the SiteScores and visual inspection of the binding poses, the hinge site located on the catalytic subunit (purple in Figure 3A) was chosen for further inspection. Induced fit docking results to this site for selected compounds are shown in Figures 3 and S22. The distance between this potential new binding site and the active site is about 15–20 Å, as shown in Figure 3C where HUP-55 is docked to both sites. Interestingly, Tyr473 at the active site, which is important for the proteolytic activity of PREP, and Tyr471 at the other binding site, which seems to be important especially for thiazole and oxazole binding, are separated by just one glycine residue.

The binding interactions and viability of the binding site were further assessed with MD simulations using Desmond.^31^ MD simulations were run for **HUP-46**, HUP-55, and HUP-28. Three residues, Tyr471, Asn483, and Ser485, were chosen for mutation to alanine based on their predicted importance to binding, which was determined by visual inspection of the interactions during the MD simulation and the calculated frequency of their interactions (Supporting Information Figures S45–S49). Two residues, Val455 and Leu499, were also chosen for point mutation to cysteine. The rationale for the cysteines was to have the option to attach covalently binding analogues of HUP-28 and compound **7**, without significantly altering the predicted binding poses of the parent compounds (Supporting Information Figure S50). The residues chosen for point mutations are highlighted in Figure 3B,C.

### Mutations at the New Binding Pocket Do Not Affect Proteolytic Activity

**Figure 4:**
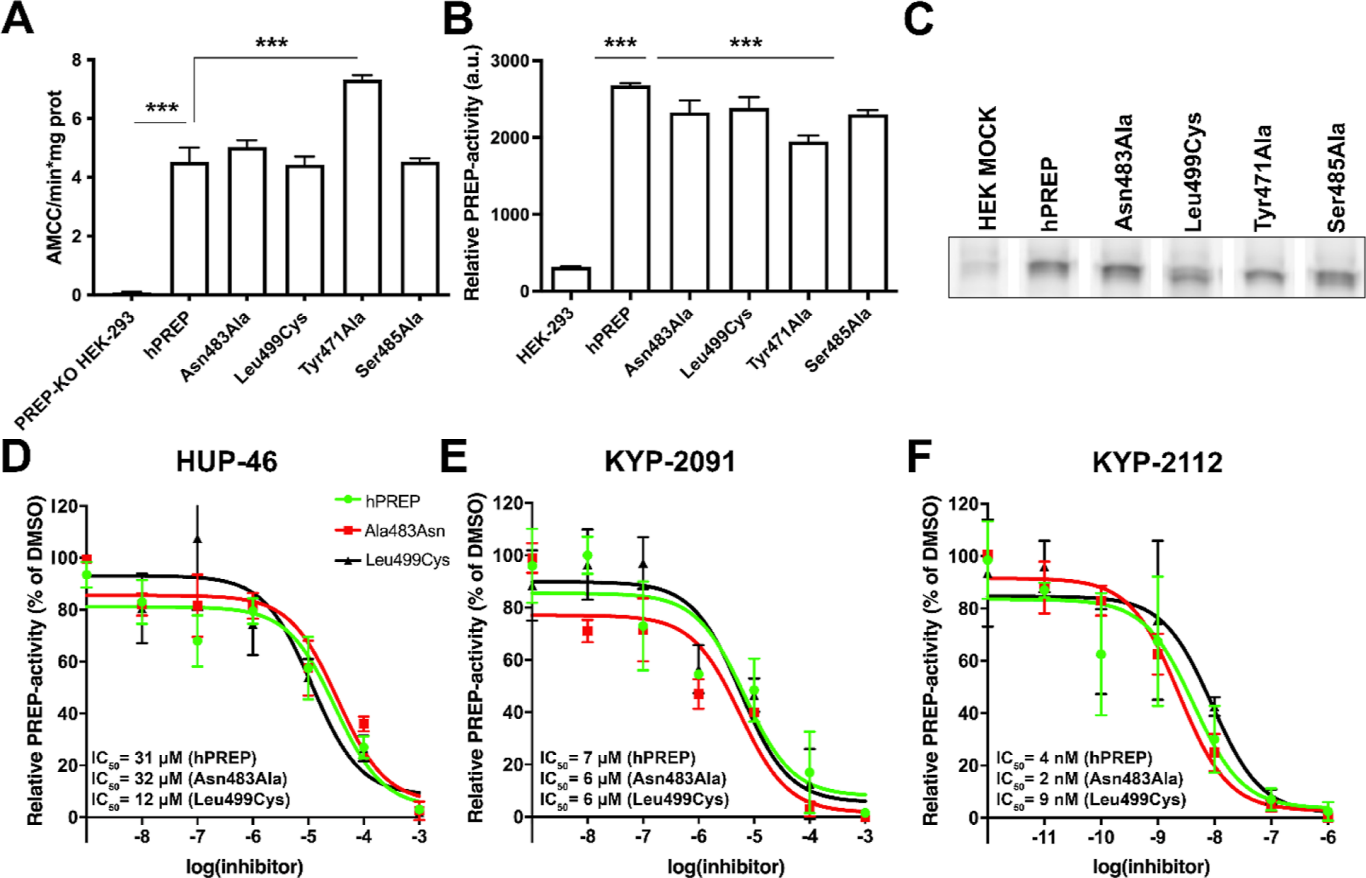
Characterization of PREP with mutations in the novel binding pocket. Tyr471Ala PREP showed increased activity when transfected to PREP knockout HEK-293 cells in the fluorogenic substrate-based activity assay (A) but had reduced activity in activity-based protein profiling assay [ABPP; (B)]. Representative bands from the ABPP assay (C). The inhibition of PREP proteolytic activity by **HUP-46**, KYP-2091, and KYP-2112 was tested in recombinant hPREP and Asn483Ala and Leu499Cys PREP mutants by using the fluorogenic substrate-based assay (D–F). ***, *p* < 0.001, one-way ANOVA with Tukey’s posthoc test.

Based on molecular modeling and preliminary biological characterization, we prepared PREP constructs with the following point mutations at the putative new binding pocket: Tyr471Ala, Asn483Ala, Ser485Ala, and Leu499Cys. PREP-KO HEK-293 cells were transfected with hPREP and the mutant constructs, and their enzymatic activity was assessed with a fluorogenic substrate-based assay (Suc-Gly-Pro-AMC) and with activity-based protein profiling (ABPP). The results show that the active site functions remain mostly unchanged in PREP-KO HEK-293 cells transfected with different PREP mutants in the fluorogenic assay (Figure 4A; PREP protein levels after transfection and full ABPP membrane with loading control are shown in Supporting Information Figure S54) and in the ABPP assay (Figure 4B). Interestingly, Tyr471Ala PREP showed elevated PREP activity in the substrate-based assay (Figure 4A; *p* < 0.001 compared to hPREP, one-way ANOVA with Tukey’s posthoc test) but decreased signal in the ABPP assay (Figure 4B,C; *p* < 0.001 compared to hPREP, one-way ANOVA with Tukey’s posthoc test). Tyr471 is the closest point to the active site, and mutations in that residue may also change the proteolytic activity. On the other hand, the ABPP assay shows the availability of the active serine (Ser554) for the ABPP probe, and this may be different than the substrate–cleavage activity.

Additionally, the sensitivity of PREP mutants to **HUP-46** and peptidic PREP ligands KYP-2091^32^ and KYP-2112^33^ was tested using mutant recombinant hPREP (Asn483Ala and Leu499Cys) in a fluorogenic assay (Figure 4E,F). KYP-2112 is a potent PREP inhibitor (IC_50_ 0.32 nM)^33^ with no PPI-related effects, and KYP-2091 is a weak inhibitor (IC_50_ 1 μM)^32^ with moderate PPI effects.^17^ When measuring IC_50_ using the fluorogenic Suc-Gly-Pro-AMC substrate in hPREP and the Asn483Ala and Leu499Cys mutants, no clear differences between hPREP and mutants were seen in any of the compounds (Figure 4D–F). Moreover, the baseline activity between mutants and hPREP was not changed in the assay. In the ABPP assay, the ability of a compound to prevent the ABPP probe from binding to the active serine of PREP was tested in hPREP and the mutants. The results obtained with each of the test compounds did not significantly differ between hPREP and the mutants (Supporting Information Figure S55A–C). Taken together, it appears that mutations in the new binding site do not have any effect on the proteolytic activity of PREP.

### HUP-46 Favors the New Binding Pocket in a Cellular Thermal Shift Assay

To characterize the binding of different ligands to the new binding pocket, we performed a cellular thermal shift assay (CETSA) with PREP-KO HEK-293 cells that were transfected with hPREP or PREP mutant constructs. KYP-2091 and KYP-2112 were used as the reference compounds for **HUP-46**.

**Figure 5:**
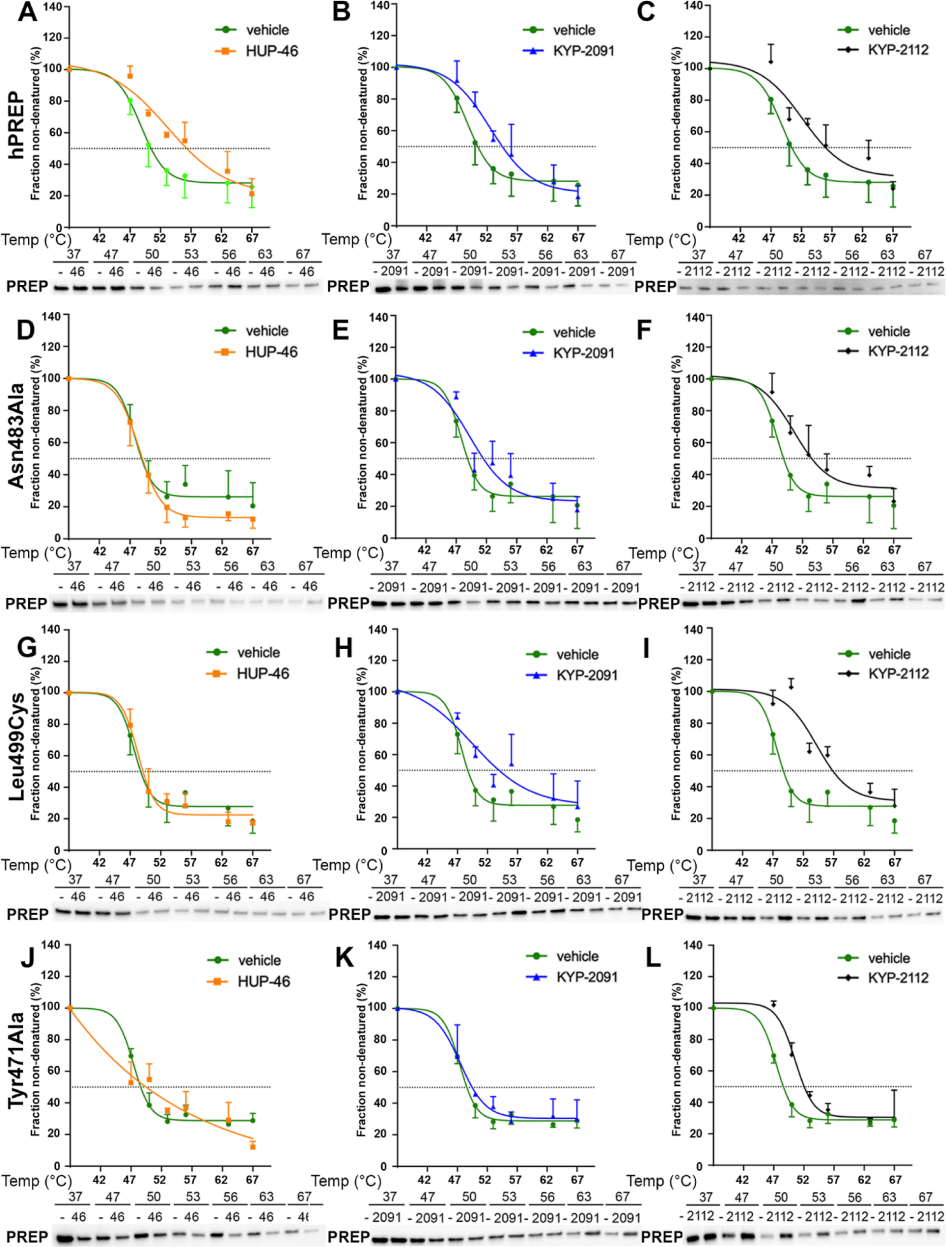
Cellular Thermal Shift Assay (CETSA) of **HUP-46**, KYP-2091, and KYP-2112 in hPREP-, Asn483Ala-, Leu499Cys-, or Tyr471Ala-transfected PREP knockout (PREP-KO) HEK-293 cells. All compounds showed binding to hPREP (A–C). **HUP-46** did not bind to Asn483Ala PREP (D), KYP-2091 showed reduced binding (E), but KYP-2112 did bind normally (F). Similar effect was seen in other mutants as well (G–L).

CETSA analysis showed that all compounds bind to hPREP (Figure 5A–C), and the aggregation temperatures (*T*_agg_) are presented in Table 2. Mutations of Asn483Ala, Leu499Cys, and Tyr471Ala completely abolished the binding of **HUP-46** (Figure 5D,G,J; Table 2) but had no effect on the binding of KYP-2112 (Figure 5F,I,L; Table 2). Compared to KYP-2112, KYP-2091 showed reduced binding to the Leu499Cys and Asn483Ala mutants and no binding to the Tyr471Ala mutant in this cell-based assay (Figure 5E,H,K). CETSA results for Ser485Ala were nonconclusive, and they were left out of this analysis. The reduced binding of KYP-2091 to the Asn483Ala and Leu499Cys mutants was verified using an isothermal titration calorimetry (ITC) assay, indicating that its binding constant (*K*_d_) was increased fourfold in both mutants. The Leu499Cys mutation had no effect on the binding of KYP-2112, and the Asn483Ala mutation even improved the *K*_d_ value of KYP-2112 (Table 3; see Supporting Information Tables S2 and S3 and Figures S56–S59 for further details).

**Table 2:** Aggregation Temperature (*T*_agg_) for **HUP-46**, KYP-2091, and KYP-2112 in PREP and PREP Mutants^a^

*T*_agg_ (°C)	hPREP	Asn483Ala	Leu499Cys	Tyr471Ala
DMSO	48.9 ± 0.4	47.8 ± 0.4	47.7 ± 0.4	47.2 ± 0.5
**HUP-46**	53.2 ± 1.5	48.5 ± 0.4	48.3 ± 0.4	n.d.
KYP-2091	52.5 ± 1.0	49.3 ± 1.2	50.6 ± 4.6	47.8 ± 0.8
KYP-2112	52.2 ± 1.3	50.6 ± 1.0	54.2 ± 0.9	50.5 ± 0.5

a n.d.: not detected.

**Table 3:** *K*_d_ Values for Binding to PREP and Two Mutants for KYP-2112 and KYP-2091, Determined Using ITC^a^

	*K*_d_ (nM)	
protein	KYP-2112	KYP-2091
wt hPREP	90 ± 28	520 ± 174
Asn483Ala	38 ± 3*	2267 ± 988**
Leu499Cys	82 ± 3	1863 ± 466*

a KYP-2091 has a higher *K*_d_ value when binding to mutated PREP compared to wild-type PREP. Similar effect was not observed with KYP-2112. *p < 0.05. **p < 0.01; Student’s *t* test.

**Figure 6:**
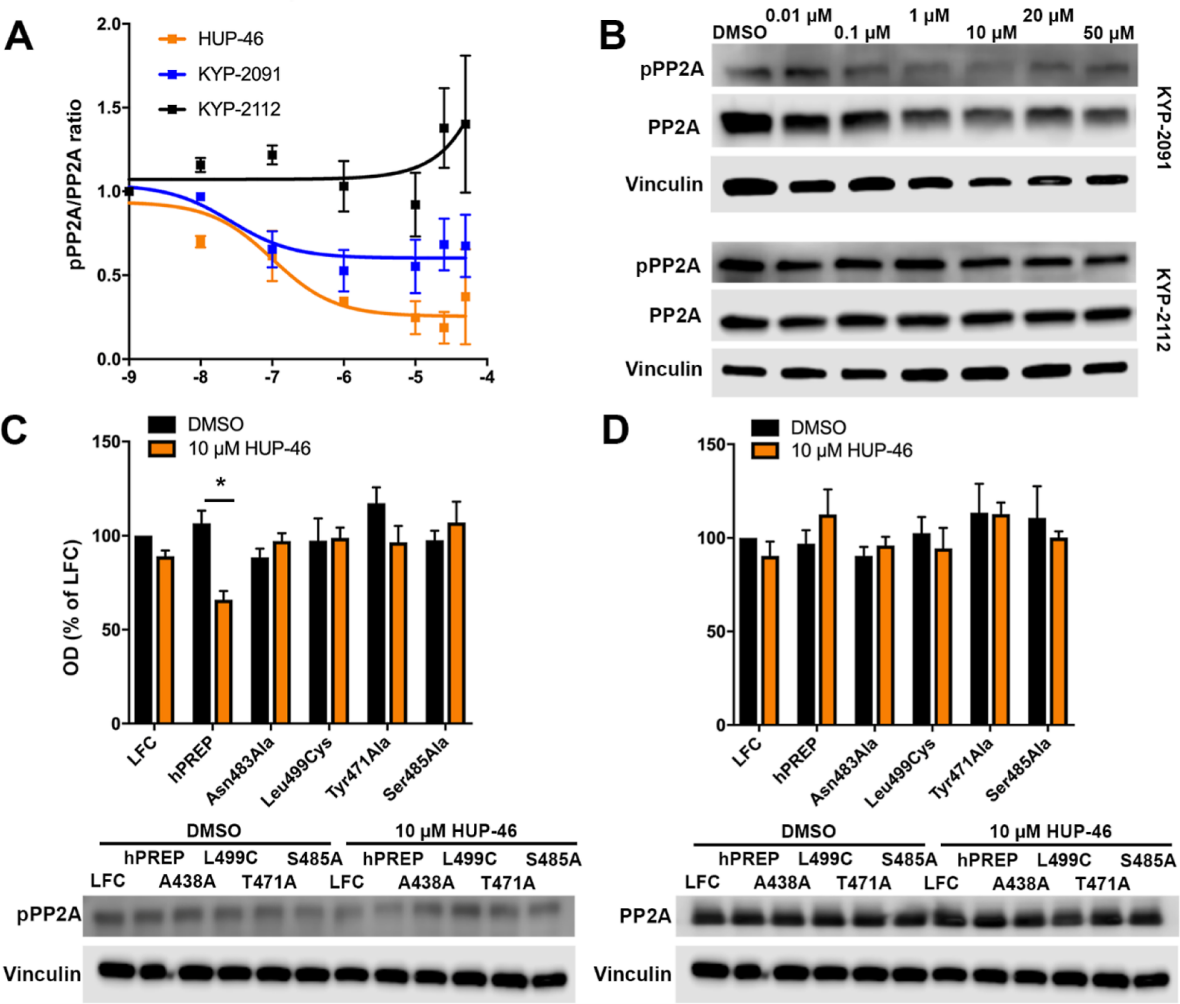
Efficacy of KYP-2091 and KYP-2112, and **HUP-46** on mutant PREP-transfected PREP knockout (PREP-KO) HEK-293 cells on PP2A activation. KYP-2112 showed no effect on PP2A activation, and KYP-2091 was less effective than **HUP-46** (A). Representative Western blot bands (B). **HUP-46** had an effect on inactive PP2A (pPP2A) in hPREP-transfected PREP-KO cells but not in mutant PREP-transfected PREP-KO cells (C). Similarly, **HUP-46** had no effect on PP2A levels in PREP mutants but increased total PP2A mildly in hPREP transfected cells (D). *, *p* < 0.05, two-way ANOVA with Sidak’s posthoc test (C).

When comparing their biological efficacy in PPI-related functions, there was a clear correlation between binding to the new binding pocket and PP2A activation when determined by the pPP2A/PP2A ratio (Figure 6A,B). KYP-2112 did not decrease the pPP2A/PP2A ratio, and KYP-2091 had a maximal effect of 0.55 (Figure 6A). Additionally, **HUP-46** did not have an effect on PP2A activation in PREP-KO HEK-293 cells expressing Asn483Ala, Leu499Cys, Tyr471Ala, or Ser485Ala PREP and showed efficacy in pPP2A only in hPREP-transfected cells (Figure 6C,D). Similar results were seen in LC3BII levels, where HUP-46 had an impact on hPREP-transfected cells but not in mutant-transfected cells (Supporting Information Figure S60).

## Conclusions

The novel 5-aminothiazoles are more potent and effective modulators of the PPI-derived functions of PREP and are advantageous over the previously reported 5-aminooxazoles as they are weaker inhibitors of the proteolytic activity of PREP, and as evidence shows, strong inhibition of the proteolytic activity is not required for the effective modulation of the PPI-mediated functions. Furthermore, 5-aminothiazoles are generally more stable than 5-aminooxazoles, allowing a wider variation of different substituents. The novel 5-aminothiazoles **HUP-46**, **7**, and **16h** are among the most effective modulators of the PPI-related functions of PREP reported to date, although they have only weak, micromolar inhibitory potency. Although **HUP-46** is the most effective of these three in biological screening assays, the other two clearly demonstrate that the electrophilic nitrile group and the lipophilic phenylpropyl group can be replaced in the structure. **HUP-46** was subjected to further cellular characterization, where it significantly reduced the ratio of pPP2A/PP2A, increased the autophagy marker LC3BII levels, and decreased αSyn dimerization in a concentration–response manner. The effect was confirmed to be PREP-specific by using PREP-KO cells. Furthermore, it should be highlighted that in addition to **HUP-46** being a more effective modulator of PP2A activity compared to the corresponding oxazole HUP-55, it is also a more potent modulator of PP2A activity. As **HUP-46** is also a brain-penetrating compound, it is a useful molecular probe for the development of therapeutic compounds for neurodegenerative diseases.

Our hypothesis explaining the disconnected SARs was that the PPI-mediated functions of PREP are not modulated through the proteolytic active site. Using molecular modeling, we identified a potential new binding site inside the cavity of PREP, which was clearly separate from the proteolytic active site. MD simulations were used to examine the most important residues for binding to this site, and based thereon, PREP constructs with different point mutations at the postulated new binding site were prepared. Mutations at the new binding site did not have significant effects on the proteolytic activity of PREP or on the inhibitory potency of the compounds. The effect of point mutations on ligand binding was determined using CETSA and ITC. **HUP-46** and KYP-2091, both weak inhibitors of PREP but effective modulators of PPI-mediated functions, were more affected by the mutations than KYP-2112, a potent inhibitor of PREP with no activity on the PPI-mediated functions. Additionally, unlike in cells expressing wild-type PREP, **HUP-46** was unable to affect PP2A, pPP2A, or LC3BII levels in cells expressing PREP with the different point mutations. We therefore conclude that there is another ligand-binding site inside the cavity of PREP, located around the described point mutations, which is more important for the modulation of the PPI-mediated functions of PREP than the proteolytic active site.

## Experimental Section

### Chemistry

#### General Information

Unless otherwise specified, all reagents and solvents were obtained from commercial suppliers and used without purification. Microwave reactions were performed with a fixed hold time in capped microwave vials using a Biotage Initiator+ (Biotage). Completion of reactions and purifications were monitored with TLC, which was performed on 60 F_254_ silica gel plates, using UV light (254 and 366 nm) and ninhydrin or iodine staining to detect products. Flash chromatography was performed manually with silica gel (230–400 μm mesh) or using a Biotage Isolera One (Biotage) with silica gel 60 (40–63 μm mesh), unless otherwise specified. ^1^H and ^13^C NMR spectra were recorded at 400 and 101 MHz, respectively, using an Ascend 400 (Bruker). CDCl_3_ was used as the NMR solvent unless otherwise specified. Chemical shifts (δ) are reported in parts per million (ppm) with TMS or solvent residual peaks as reference. Exact mass and purity of the tested compounds were analyzed with LC–MS, using a Waters Aquity UPLC system (Waters) and a Waters Synapt G2 HDMS mass spectrometer (Waters) via an ESI ion source in positive mode. The synthesis of peptidic starting materials **15a**–**l** is reported in the Supporting Information. The purity of all tested final compounds was 95% or higher, except compound **8**, which had a purity of 94%.

#### Synthesis of Compounds

##### 2,5-Dibromo-4-methylthiazole (**2**)

4-Methylthiazole (4 mL, 44 mmol) was dissolved in anhydrous DMF (40 mL) under a constant stream of Ar. CBr_4_ (30.6 g, 92 mmol) was added and allowed to dissolve completely. The solution was cooled to −10 °C, and sodium *t*-butoxide (16.9 g, 176 mmol) was added slowly. The mixture was left to stir at room temperature for 1.5 h, before pouring it into cold water and extracting with DCM. The organic phase was washed with H_2_O, dried over anhydrous Na_2_SO_4_, filtered, and evaporated to obtain the crude product as a black oil, which after flash chromatography (heptane/DCM 2:1 → 1:1) yielded **2** as a brown oil (6.48 g, 57%). ^1^H NMR: δ 2.38 (s, 3H). ^13^C NMR: δ 152.56, 134.10, 105.98, 15.75.

##### Method A: Synthesis of (*E*)-5-Bromo-4-methyl-2-(3-phenylprop-1-en-1-yl)thiazole (**3**)

Compound **2** (583 mg, 2.27 mmol) in dioxane (18 mL), H_2_O (2 mL), and Li_2_CO_3_ (335 mg, 4.54 mmol) were added to a flask containing *trans*-3-phenyl-1-propen-1-ylboronic acid (0.368 mg, 2.27 mmol) and Pd(PPh_3_)_4_ (226 mg, 0.20 mmol) under Ar. The mixture was stirred at 100 °C for 24 h before diluting with H_2_O and extracting with DCM. The organic phase was dried over anhydrous Na_2_SO_4_, filtered, and evaporated to provide the crude product as a brown oil, which after flash chromatography (heptane/EtOAc 49:1 → 4:1) yielded compound **3** as an orange oil (483 mg, 72%). ^1^H NMR: δ 7.38–7.30 (m, 2H), 7.30–7.20 (m, 3H), 6.64 (dt, *J* = 15.8, 6.6 Hz, 1H), 6.52 (dt, *J* = 15.8, 1.4 Hz, 1H), 3.65–3.48 (m, 2H), 2.38 (s, 3H). ^13^C NMR: δ 165.91, 151.89, 138.34, 136.55, 128.84, 128.70, 126.62, 124.71, 102.88, 39.01, 15.62.

##### (*E*)-5-Bromo-4-methyl-2-styrylthiazole (**4**)

This was synthesized according to method A using *trans*-2-phenylvinylboronic acid (1.11 g, 6.89 mmol). The crude product was obtained, which after flash chromatography (heptane/EtOAc 24:1 → 9:1) yielded compound **4** (235 mg, 12%). ^1^H NMR: δ 7.47–7.41 (m, 2H), 7.34–7.25 (m, 3H), 7.22 (d, *J* = 16.2 Hz, 1H), 7.09 (d, *J* = 16.2 Hz, 1H), 2.34 (s, 3H). ^13^C NMR: δ 166.04, 152.59, 135.64, 134.58, 129.24, 129.05, 127.26, 121.27, 103.65, 15.83.

##### Method B: Synthesis of (*E*)-5-Bromo-4-methyl-2-(3-phenylprop-1-en-1-yl)thiazole (**5**)

A solution of *N*,*O*-bis(trifluoroacetyl)hydroxylamine (2.18 g, 9.71 mmol) in dioxane (20 mL) was added to a solution of compound **3** (1.90 g, 6.47 mmol) and NH_2_OH (50% in H_2_O, 2.16 mL, 32.4 mmol) in dioxane (20 mL) under Ar. The mixture was stirred at reflux for 22 h before diluting with saturated NaHCO_3_ and extracting with EtOAc. The organic phase was washed with H_2_O and brine, dried over anhydrous Na_2_SO_4_, filtered, and evaporated to provide the crude product, which after flash chromatography (heptane/EtOAc 49:1 → 4:1) yielded compound **5** as a yellow oil (1.06 g, 55%). The product contained ca. 20% unreacted starting material. ^1^H NMR: δ 7.41–7.13 (m, 5H), 2.98–2.86 (m, 2H), 2.76–2.65 (m, 2H), 2.36 (s, 3H), 2.13–1.99 (m, 2H). ^13^C NMR: δ 170.22, 151.14, 141.37, 128.61, 128.58, 126.18, 102.60, 35.21, 33.43, 31.44, 15.74.

##### 5-Bromo-4-methyl-2-phenethylthiazole (**6**)

This was synthesized according to method B using compound **4** (234 mg, 0.84 mmol). The crude product was obtained, which after flash chromatography (heptane/EtOAc 24:1 → 6:1) yielded compound **6** (114 mg, 48%), which was used in the next step without further characterization.

##### Method C: Synthesis of 4-Methyl-2-(3-phenylpropyl)-5-(pyrrolidin-1-yl)thiazole (**7**)

Compound **5** (100 mg, 0.44 mmol), pyrrolidine (0.11 mL, 1.33 mmol), *t*BuONa (85 mg, 0.88 mmol), Rh(cod)_2_BF_4_ (3.6 mg, 0.01 mmol), and 1,3-diisopropylimidazolium chloride (3.3 mg, 0.02 mmol) were dissolved in anhydrous dimethoxyethane (2 mL) under Ar. The mixture was heated to 80 °C for 19 h before cooling to room temperature, diluting with EtOAc, and filtering through silica. The filtrate was evaporated to obtain the crude product, which after flash chromatography (heptane/EtOAc 49:1 → 4:1) yielded compound **7** (167 mg, 38%). If alkene remained and could not be separated from the final product, it was reduced to the correct product using an H-cube with standard conditions for reduction. ^1^H NMR: δ 7.25–7.16 (m, 2H), 7.16–7.05 (m, 3H), 3.04–2.93 (m, 4H), 2.85–2.75 (m, 2H), 2.67–2.58 (m, 2H), 2.22 (s, 3H), 2.05–1.93 (m, 2H), 1.93–1.81 (m, 4H). ^13^C: δ 160.23, 145.04, 141.75, 138.10, 128.50, 128.35, 125.87, 55.53, 35.29, 33.77, 31.79, 25.05, 14.88. HRMS (ESI-QTOF) *m*/*z*: [M + H]^+^ calcd for C_17_H_23_N_2_S, 287.1582; found, 287.1581.

##### 4-Methyl-2-(3-phenylpropyl)-5-(piperidin-1-yl)thiazole (**8**)

This was synthesized according to method C using piperidine (83 mg, 0.98 mmol). The crude product was obtained, which after flash chromatography (heptane/EtOAc 9:1) yielded compound **8** (54 mg, 55%). ^1^H NMR: δ 7.25–7.05 (m, 5H), 2.89–2.79 (m, 2H), 2.72–2.57 (m, 6H), 2.19 (s, 3H), 2.05–1.93 (m, 2H), 1.66–1.56 (m, 4H), 1.50–1.38 (m, 2H). ^13^C NMR: δ 163.18, 147.68, 141.84, 141.06, 128.62, 128.48, 126.00, 56.63, 35.43, 34.19, 31.85, 26.29, 23.85, 14.53. HRMS (ESI-QTOF) *m*/*z*: [M + H]^+^ calcd for C_18_H_24_N_2_S, 301.1739; found, 301.1740.

##### (*S*)-(1-(4-Methyl-2-(3-phenylpropyl)thiazol-5-yl)pyrrolidin-2-yl)methanol (**10**)

This was synthesized according to method C using l-prolinol (367 mg, 3.6 mmol). The crude product was obtained, which after flash chromatography (heptane/EtOAc 9:1 → EtOAc) yielded compound **10** (21 mg, 5%). ^1^H NMR: δ 7.31–7.25 (m, 2H), 7.21–7.15 (m, 3H), 3.54 (dd, *J* = 11.2, 3.9 Hz, 1H), 3.50–3.42 (m, 1H), 3.42–3.35 (m, 1H), 3.15–3.08 (m, 1H), 2.93–2.86 (m, 2H), 2.86–2.78 (m, 1H), 2.74–2.68 (m, 2H), 2.28 (s, 3H), 2.12–1.87 (m, 6H). ^13^C NMR: δ 164.61, 144.03, 143.80, 141.69, 128.60, 128.49, 126.04, 68.52, 63.09, 58.28, 35.39, 34.20, 31.69, 27.77, 24.64, 14.78. HRMS (ESI-QTOF) *m*/*z*: [M + H]^+^ calcd for C_18_H_24_N_2_OS, 317.1688; found, 317.1689.

##### Method D: Synthesis of (*S*)-1-(4-Methyl-2-(3-phenylpropyl)thiazol-5-yl)pyrrolidine-2-carboxamide (**11**)

Compound **5** (659 mg, 2.23 mmol), l-prolinamide (609 mg, 5.34 mmol), and Cs_2_CO_3_ (2.54 g, 7.79 mmol) were dissolved in anhydrous DMF (11 mL) under Ar in an oven-dried MW vial. The mixture was heated to 180 °C for 30 min in a MW reactor before diluting with H_2_O and extracting with EtOAc. The organic phase was dried over anhydrous Na_2_SO_4_, filtered, and evaporated to provide the crude product as a brown oil, which after flash chromatography (EtOAc/MeOH 49:1 → 4:1) yielded compound **11** as a brown sap (296 mg, 40%). ^1^H NMR: δ 7.35–7.12 (m, 5H), 6.73 (s, 1H), 5.94 (s, 1H), 3.66 (dd, *J* = 9.3, 5.1 Hz, 1H), 3.58–3.48 (m, 1H), 2.94–2.83 (m, 3H), 2.70 (t, *J* = 7.6 Hz, 2H), 2.43–2.36 (m, 1H), 2.33 (s, 3H), 2.19–1.86 (m, 5H). ^13^C NMR: δ 176.35, 163.67, 143.96, 141.92, 141.57, 128.58, 128.49, 126.05, 70.43, 57.95, 35.32, 33.94, 31.60, 31.38, 25.06, 15.01.

##### (*R*)-5-(3-Fluoropyrrolidin-1-yl)-4-methyl-2-(3-phenylpropyl)thiazole (**9**)

This was synthesized according to method D using (*R*)-3-fluoropyrrolidine hydrochloride (74 mg, 0.59 mmol). The crude product was obtained, which after flash chromatography (heptane/EtOAc 9:1 → 4:1) yielded compound **9** (22 mg, 30%). ^1^H NMR: δ 7.28–7.06 (m, 5H), 5.30–5.08 (m, 1H), 3.38–3.14 (m, 3H), 3.08–2.96 (m, 1H), 2.87–2.77 (m, 2H), 2.68–2.58 (m, 2H), 2.22 (s, 3H), 2.21–2.05 (m, 2H), 2.05–1.93 (m, 2H). ^13^C NMR: δ 161.93, 143.88, 141.76, 140.34, 128.61, 128.49, 126.03, 93.45 (d, *J* = 176.4 Hz), 62.00 (d, *J* = 23.1 Hz), 53.77, 35.37, 33.94, 33.43 (d, *J* = 22.0 Hz), 31.81, 14.82. HRMS (ESI-QTOF) *m*/*z*: [M + H]^+^ calcd for C_17_H_21_FN_2_S, 305.1488; found, 305.1489.

##### 4-Methyl-2-phenethyl-5-(pyrrolidin-1-yl)thiazole (**12**)

This was synthesized according to method D using compound **6** (114 mg, 0.41 mmol) and pyrrolidine (0.08 mL, 0.97 mmol). The crude product was obtained, which after flash chromatography (heptane/EtOAc 8:1 → 3:1) yielded compound **12** (25 mg, 23%). ^1^H NMR: δ 7.31–7.05 (m, 5H), 3.11–3.04 (m, 2H), 3.04–2.94 (m, 6H), 2.25 (s, 3H), 1.93–1.82 (m, 4H). ^13^C NMR: δ 159.46, 145.33, 140.95, 138.17, 128.60, 128.56, 126.32, 55.64, 36.44, 36.15, 25.19, 15.02. HRMS (ESI-QTOF) *m*/*z*: [M + H]^+^ calcd for C_16_H_20_N_2_S, 273.1425; found, 273.1428.

##### (*S*)-1-(4-Methyl-2-(3-phenylpropyl)thiazol-5-yl)pyrrolidine-2-carbonitrile (HUP-46)

TFAA (0.11 mL, 0.79 mmol) in anhydrous DCM (5.5 mL) was added slowly to a solution of compound **11** (218 mg, 0.88 mmol) and Et_3_N (0.22 mL, 1.6 mmol) in anhydrous DCM (2.5 mL) under Ar at 0 °C. The solution was stirred at room temperature for 2 h before quenching with H_2_O. The organic phase was washed with a 10% aqueous solution of citric acid, a saturated solution of NaHCO_3_, and brine, dried over anhydrous Na_2_SO_4_, filtered, and evaporated to provide the crude product as a brown oil, which after flash chromatography (heptane/EtOAc 9:1 → 1:4) yielded **HUP-46** as an orange oil (103 mg, 50%). ^1^H NMR: δ 7.35–7.26 (m, 2H), 7.26–7.18 (m, 3H), 4.02 (dd, *J* = 7.7, 3.8 Hz, 1H), 3.29 (ddd, *J* = 9.2, 8.1, 5.4 Hz, 1H), 3.16 (ddd, *J* = 9.3, 7.8, 6.3 Hz, 1H), 2.99–2.89 (m, 2H), 2.80–2.70 (m, 2H), 2.44–2.26 (m, 2H), 2.34 (s, 3H), 2.26–2.03 (m, 4H). ^13^C NMR: δ 165.38, 145.22, 141.60, 139.66, 128.61, 128.50, 126.06, 119.04, 55.86, 55.00, 35.35, 34.10, 31.61, 31.23, 23.76, 14.80. HRMS (ESI-QTOF) *m*/*z*: [M + H]^+^ calcd for C_18_H_22_N_3_S, 312.1534; found, 312.1534.

##### (*S*)-5-(2-(1*H*-Tetrazol-5-yl)pyrrolidin-1-yl)-4-methyl-2-(3-phenylpropyl)thiazole (**13**)

ZnBr_2_ (91 mg, 0.40 mmol) and NaN_3_ (29 mg, 0.45 mmol) were added to a suspension of compound **HUP-46** (126 mg, 0.40 mmol) in H_2_O (2 mL), and the resulting suspension was stirred vigorously at reflux for 20 h. The mixture was cooled to room temperature, and 4 M HCl (0.6 mL) was added. The aqueous phase was extracted with EtOAc, and the combined organic phases evaporated. The resulting residue was dissolved in 0.25 M NaOH (8 mL) and filtered. The filtrate was acidified with 4 M HCl, saturated with NaCl, and extracted with EtOAc. The organic phase was dried over anhydrous Na_2_SO_4_, filtered, and evaporated to provide the crude product as an orange oil, which after flash chromatography (EtOAc/MeOH 19:1 → 4:1) yielded compound **13** as a yellow oil (26 mg, 18%). ^1^H NMR (methanol-*d*_4_): δ 7.31–7.06 (m, 6H), 4.65–4.54 (m, 1H), 3.63–3.50 (m, 1H), 3.13–3.02 (m, 1H), 2.86–2.76 (m, 2H), 2.63 (t, *J* = 7.6 Hz, 2H), 2.58–2.46 (m, 1H), 2.36–2.07 (m, 4H), 2.02 (s, 3H), 2.00–1.93 (m, 2H). ^13^C NMR (methanol-*d*_4_): δ 167.10, 159.77, 144.54, 143.65, 142.68, 129.48, 129.41, 126.99, 61.74, 58.06, 35.96, 34.28, 33.40, 32.81, 25.37, 14.09. HRMS (ESI-QTOF) *m*/*z*: [M + H]^+^ calcd for C_18_H_23_N_6_S, 355.1705; found, 355.1707.

##### Method E: Synthesis of 2-(3-(4-Iodophenyl)propyl)-4-methyl-5-(pyrrolidin-1-yl)thiazole (**16c**)

Lawesson’s reagent (110 mg, 0.27 mmol) was added to compound **15c** (94 mg, 0.23 mmol) in anhydrous pyridine (1 mL) in a MW vial. The mixture was heated to 150 °C for 30 min in a MW before diluting with EtOAc and washing with H_2_O and brine. The organic phase was dried over anhydrous Na_2_SO_4_, filtered, and evaporated to provide the crude product as a yellow sap, which after flash chromatography, first with a regular silica column (heptane/EtOAc 22:3 → EtOAc), followed by an amine-functionalized column (heptane/EtOAc 19:1 → 4:1), yielded **16c** as a yellow oil (54 mg, 57%). ^1^H NMR: δ 7.61–7.45 (m, 2H), 6.95–6.80 (m, 2H), 3.07–2.91 (m, 4H), 2.85–2.74 (m, 2H), 2.57 (t, *J* = 7.7 Hz, 2H), 2.22 (s, 3H), 2.01–1.90 (m, 2H), 1.90–1.81 (m, 4H). ^13^C NMR: δ 159.85, 145.24, 141.49, 138.20, 137.49, 130.76, 91.03, 55.64, 34.84, 33.71, 31.62, 25.18, 15.01. HRMS (ESI-QTOF) *m*/*z*: [M + H]^+^ calcd for C_17_H_22_N_2_SI, 413.0548; found, 413.0545.

##### 2-(3-(3,4-Dimethoxyphenyl)propyl)-4-methyl-5-(pyrrolidin-1-yl)thiazole (**16a**)

This was synthesized according to method E using **15a** (87 mg, 0.25 mmol) with a reaction time of 15 min. The crude product was obtained, which after flash chromatography, first with a regular silica column, followed by an amine-functionalized column (heptane/EtOAc 9:1 → EtOAc), yielded **16a** as a pale yellow oil (21 mg, 24%). ^1^H NMR: δ 6.82–6.76 (m, 1H), 6.76–6.70 (m, 2H), 3.86 (d, *J* = 6.6 Hz, 6H), 3.10–3.01 (m, 4H), 2.91–2.82 (m, 2H), 2.69–2.61 (m, 2H), 2.30 (s, 3H), 2.09–1.98 (m, 2H), 1.98–1.88 (m, 4H). ^13^C NMR: δ 160.32, 148.93, 147.33, 145.14, 138.22, 134.51, 120.42, 111.94, 111.35, 56.06, 55.94, 55.65, 34.99, 33.82, 32.07, 25.17, 15.00. HRMS (ESI-QTOF) *m*/*z*: [M + H]^+^ calcd for C_19_H_27_N_2_O_2_S, 347.1793; found, 347.1794.

##### 4-(3-(4-Methyl-5-(pyrrolidin-1-yl)thiazol-2-yl)propyl)benzonitrile (**16b**)

This was synthesized according to method E using **15b** (65 mg, 0.21 mmol). The crude product was obtained, which after flash chromatography using an amine-functionalized column (heptane/EtOAc 9:1 → EtOAc) yielded **16b** as a pale yellow oil (30 mg, 46%). ^1^H NMR: δ 7.49 (d, *J* = 8.3 Hz, 1H), 7.22 (d, *J* = 8.3 Hz, 2H), 3.02–2.95 (m, 4H), 2.84–2.77 (m, 2H), 2.73–2.64 (m, 2H), 2.22 (s, 3H), 2.04–1.93 (m, 2H), 1.90–1.81 (m, 4H). ^13^C NMR: δ 159.23, 147.53, 145.34, 138.12, 132.29, 129.40, 119.18, 109.89, 55.59, 35.39, 33.58, 31.23, 25.17, 14.99. HRMS (ESI-QTOF) *m*/*z*: [M + H]^+^ calcd for C_18_H_22_N_3_S, 312.1534; found, 312.1535.

##### 4-Methyl-2-(3-(pyridin-2-yl)propyl)-5-(pyrrolidin-1-yl)thiazole (**16d**)

This was synthesized according to method E using **15d** (162 mg, 0.56 mmol). The crude product was obtained, which after flash chromatography using an amine-functionalized column (heptane/EtOAc 9:1 → EtOAc) yielded **16d** as a pale yellow oil (76 mg, 47%). ^1^H NMR: δ 8.44 (ddd, *J* = 4.9, 1.9, 1.0 Hz, 1H), 7.49 (td, *J* = 7.6, 1.9 Hz, 1H), 7.08 (dt, *J* = 7.8, 1.1 Hz, 1H), 7.01 (ddd, *J* = 7.5, 4.9, 1.2 Hz, 1H), 3.02–2.90 (m, 4H), 2.88–2.74 (m, 4H), 2.22 (s, 3H), 2.16–2.05 (m, 2H), 1.95–1.80 (m, 4H). ^13^C NMR: δ 161.36, 159.96, 149.24, 145.08, 138.04, 136.24, 122.85, 121.03, 55.47, 37.56, 33.72, 30.04, 25.00, 14.83. HRMS (ESI-QTOF) *m*/*z*: [M + H]^+^ calcd for C_16_H_22_N_3_S, 288.1534; found, 288.1532.

##### 4-Methyl-2-(3-(pyridin-3-yl)propyl)-5-(pyrrolidin-1-yl)thiazole (**16e**)

This was synthesized according to method E using **15e** (67 mg, 0.23 mmol). The crude product was obtained, which after flash chromatography using an amine-functionalized column (heptane/EtOAc 9:1 → EtOAc) yielded **16e** as a pale yellow oil (24 mg, 36%). ^1^H NMR: δ 8.40–8.38 (m, 1H), 8.37 (dd, *J* = 4.8, 1.7 Hz, 1H), 7.48–7.41 (m, 1H), 7.13 (ddd, *J* = 7.8, 4.8, 0.9 Hz, 1H), 3.06–2.93 (m, 4H), 2.84–2.77 (m, 2H), 2.67–2.59 (m, 2H), 2.22 (s, 3H), 2.04–1.94 (m, 2H), 1.90–1.83 (m, 4H). ^13^C NMR: δ 159.46, 150.12, 147.58, 145.29, 138.19, 137.02, 135.96, 123.39, 55.60, 33.63, 32.39, 31.45, 25.16, 14.99. HRMS (ESI-QTOF) *m*/*z*: [M + H]^+^ calcd for C_16_H_22_N_3_S, 288.1534; found, 288.1532.

##### 4-Methyl-2-(2-(pyridin-3-yl)ethyl)-5-(pyrrolidin-1-yl)thiazole (**16f**)

This was synthesized according to method E using **15f** (87 mg, 0.25 mmol). The crude product was obtained, which after flash chromatography using an amine-functionalized column (heptane/EtOAc 9:1 → EtOAc) yielded **16f** as a yellow oil (57 mg 43%). ^1^H NMR: δ 8.42–8.40 (m, 1H), 8.38 (dd, *J* = 4.8, 1.6 Hz, 1H), 7.51–7.40 (m, 1H), 7.14 (ddd, *J* = 7.8, 4.8, 0.9 Hz, 1H), 3.11–3.04 (m, 2H), 3.03–2.94 (m, 6H), 2.23 (s, 3H), 1.91–1.83 (m, 4H). ^13^C NMR: δ 158.10, 150.11, 147.87, 145.51, 138.11, 136.12, 135.95, 123.45, 55.56, 35.53, 33.29, 25.17, 15.01. HRMS (ESI-QTOF) *m*/*z*: [M + H]^+^ calcd for C_15_H_20_N_3_S, 274.1378; found, 274.1379.

##### 2-(2-(1*H*-Indol-3-yl)ethyl)-4-methyl-5-(pyrrolidin-1-yl)thiazole (**16g**)

This was synthesized according to method E using **15g** (100 mg, 0.32 mmol). The crude product was obtained, which after flash chromatography using an amine-functionalized column (heptane/EtOAc 9:1 → EtOAc) yielded **16g** as a pale yellow oil (40 mg, 40%). ^1^H NMR: δ 8.08 (s, 1H), 7.65–7.57 (m, 1H), 7.34 (dt, *J* = 8.1, 1.0 Hz, 1H), 7.18 (ddd, *J* = 8.2, 7.0, 1.3 Hz, 1H), 7.11 (ddd, *J* = 8.0, 7.0, 1.1 Hz, 1H), 7.00 (d, *J* = 2.3 Hz, 1H), 3.28–3.16 (m, 4H), 3.09–3.02 (m, 4H), 2.33 (s, 3H), 1.97–1.89 (m, 4H). ^13^C NMR: δ 160.27, 145.33, 138.20, 136.40, 127.48, 122.10, 121.66, 119.38, 118.96, 115.39, 111.22, 55.66, 35.05, 26.03, 25.18, 15.04. HRMS (ESI-QTOF) *m*/*z*: [M + H]^+^ calcd for C_18_H_22_N_3_S, 312.1535; found, 312.1536.

##### 2-(2-(1*H*-Benzo[*d*]imidazole-1-yl)ethyl)-4-methyl-5-(pyrrolidin-1-yl)thiazole (**16h**)

This was synthesized according to method E using **15h** (100 mg, 0.32 mmol). The crude product was obtained as a dark red sap, which after flash chromatography, using an amine-functionalized column (heptane/EtOAc 1:1 → EtOAc), yielded **16h** as a brown sap (10 mg, 10%). ^1^H NMR: δ 7.84 (s, 1H), 7.82–7.76 (m, 1H), 7.43–7.36 (m, 1H), 7.34–7.23 (m, 2H), 4.59 (t, *J* = 7.0 Hz, 2H), 3.35 (t, *J* = 7.0 Hz, 2H), 3.09–2.97 (m, 4H), 2.32 (s, 3H), 1.97–1.86 (m, 4H). ^13^C NMR: δ 153.72, 146.40, 143.94, 143.30, 138.21, 133.69, 123.05, 122.27, 120.58, 109.59, 55.47, 44.51, 34.23, 25.23, 15.10. HRMS (ESI-QTOF) *m*/*z*: [M + H]^+^ calcd for C_18_H_22_N_4_S, 313.1487; found, 313.1486.

##### 2-(2-(1*H*-Benzo[*d*]imidazole-2-yl)ethyl)-4-methyl-5-(pyrrolidin-1-yl)thiazole (**16i**)

This was synthesized according to method E using **15i** (64 mg, 0.20 mmol). The crude product was obtained, which after flash chromatography, using an amine-functionalized column (heptane/EtOAc 9:1 → EtOAc), yielded **16i** as a yellow solid (33 mg 52%). ^1^H NMR: δ 11.74 (s, 1H), 7.70 (s, 1H), 7.40 (s, 1H), 7.23–7.18 (m, 2H), 3.43–3.31 (m, 4H), 3.12–3.05 (m, 4H), 2.39 (s, 3H), 1.98–1.88 (m, 4H). ^13^C NMR: δ 158.44, 154.57, 146.05, 137.33, 122.35, 121.81, 119.04, 110.80, 55.47, 31.22, 28.52, 25.27, 15.12. HRMS (ESI-QTOF) *m*/*z*: [M + H]^+^ calcd for C_17_H_21_N_4_S, 313.1487; found, 313.1487.

##### 4-Isopropyl-2-(3-phenylpropyl)-5-(pyrrolidin-1-yl)thiazole (**16j**)

This was synthesized according to method E using **15j** (163 mg, 0.52 mmol). The crude product was obtained, which after flash chromatography (heptane/EtOAc 9:1 → EtOAc) yielded **16j** as a pale yellow oil (62 mg, 38%). ^1^H NMR: δ 7.31–7.24 (m, 2H), 7.22–7.15 (m, 3H), 3.14 (hept, *J* = 6.9 Hz, 1H), 3.04–2.99 (m, 4H), 2.93–2.87 (m, 2H), 2.77–2.68 (m, 2H), 2.11–2.00 (m, 2H), 1.97–1.89 (m, 4H), 1.25 (d, *J* = 6.9 Hz, 6H). ^13^C NMR: δ 162.2, 150.7, 143.9, 142.0, 128.7, 128.5, 126.0, 56.8, 35.5, 34.2, 32.0, 28.0, 25.2, 22.7. HRMS (ESI-QTOF) *m*/*z*: [M + H]^+^ calcd for C_19_H_27_N_2_S, 315.1895; found, 315.1895.

##### 2-(2-(1*H*-Indol-3-yl)ethyl)-4-isopropyl-5-(pyrrolidin-1-yl)thiazole (16k)

This was synthesized according to method E using **15k** (100 mg, 0.32 mmol). The crude product was obtained, which after flash chromatography (heptane/EtOAc 9:1 → EtOAc) yielded **16k** as a white solid (29 mg 27%). ^1^H NMR: δ 8.08 (s, 1H), 7.63–7.54 (m, 1H), 7.35 (d, *J* = 8.1 Hz, 1H), 7.18 (ddd, *J* = 8.2, 7.0, 1.2 Hz, 1H), 7.10 (ddd, *J* = 8.0, 7.0, 1.1 Hz, 1H), 7.01–6.94 (m, 1H), 3.35–3.12 (m, 4H), 3.04–2.98 (m, 4H), 1.96–1.90 (m, 4H), 1.28 (d, *J* = 7.0 Hz, 6H). ^13^C NMR: δ 162.20, 150.68, 144.16, 136.34, 127.59, 122.05, 121.67, 119.34, 118.97, 115.52, 111.17, 56.83, 35.25, 28.08, 26.01, 25.15, 22.71. HRMS (ESI-QTOF) *m*/*z*: [M + H]^+^ calcd for C_20_H_26_N_3_S, 340.1848; found, 340.1849.

##### 2-(3-Phenylpropyl)-5-(pyrrolidin-1-yl)thiazole (**16l**)

This was synthesized according to method E using **15l** (109 mg, 0.40 mmol). The crude product was obtained as a yellow sap, which after flash chromatography, first with a regular silica column (heptane/EtOAc 4:1 → heptane/EtOAc 13:7), followed by an amine-functionalized column (heptane/EtOAc 19:1 → heptane/EtOAc 4:1), yielded **16l** as a pale yellow oil (24 mg, 22%). ^1^H NMR: δ 7.32–7.23 (m, 2H), 7.23–7.13 (m, 3H), 6.47 (s, 1H), 3.26–3.14 (m, 4H), 2.94–2.81 (m, 2H), 2.73–2.66 (m, 2H), 2.11–1.93 (m, 6H). ^13^C NMR: δ 154.66, 150.92, 141.90, 128.60, 128.44, 125.93, 116.31, 51.85, 35.23, 33.01, 31.70, 25.68. HRMS (ESI-QTOF) *m*/*z*: [M + H]^+^ calcd for C_16_H_20_N_2_S, 273.1425; found, 273.1422.

##### 4-Methyl-2-(2-(1-methyl-1*H*-indol-3-yl)ethyl)-5-(pyrrolidin-1-yl)thiazole (**17**)

NaH (60%, 7.7 mg, 0.19 mmol) was added to a solution of compound **16g** (30 mg, 0.096 mmol) in anhydrous DMF (2 mL) at 0 °C. The reaction mixture was stirred at 0 °C for 30 min. Iodomethane (15 μL, 0.1 mmol) was added, and stirring was continued at room temperature for 4 d. The mixture was diluted with EtOAc, washed with water and brine, dried over anhydrous Na_2_SO_4_, filtered, and evaporated to provide the crude product, which after flash chromatography (heptane/EtOAc 9:1 → EtOAc) yielded **17** as a colorless oil (20 mg, 65%). ^1^H NMR: δ 7.61 (dd, *J* = 7.8, 0.9 Hz, 1H), 7.35–7.21 (m, 2H), 7.11 (ddd, *J* = 8.0, 6.8, 1.1 Hz, 1H), 6.88 (s, 1H), 3.74 (s, 3H), 3.28–3.17 (m, 4H), 3.10–3.03 (m, 4H), 2.34 (s, 3H), 2.02–1.85 (m, 4H). ^13^C NMR: δ 160.31, 145.28, 138.27, 137.10, 127.85, 126.44, 121.66, 119.06, 118.81, 113.90, 109.27, 55.67, 35.34, 32.72, 26.00, 25.17, 15.05. HRMS (ESI-QTOF) *m*/*z*: [M + H]^+^ calcd for C_19_H_23_N_3_S, 326.1691; found, 326.1689.

### Biology

#### Cell Cultures

Mouse neuronal Neuro2A (N2A), human embryonic kidney (HEK-293), human neuroblastoma (SH-SY5Y), and PREP knockout (PREP-KO) HEK-293 cell lines^34^ were used in this study. Apart from PREP-KO HEK-293 cells, the cells were obtained from ATCC (Manassas, VA, USA). N2A cells were cultured in Dulbecco’s modified Eagle’s medium (DMEM-Glutamax; #31966021; Thermo Fisher Scientific) with additional 10% (v/v) fetal bovine serum (FBS; #16000-044, Thermo Fisher Scientific) and 1% l-glutamine–penicillin–streptomycin solution (15140122; Thermo Fisher Scientific). HEK-293 cells were cultured in DMEM (#D6429, Sigma) with additional 10% FBS and 1% l-glutamine–penicillin–streptomycin solution (15140122; Thermo Fisher Scientific). PREP-KO HEK-293 cells were cultured similar to HEK-293 cells, but they had 20% FBS supplement. SH-SY5Y cells were cultured in DMEM–Glutamax with 15% (v/v) FBS, 1% nonessential amino acids (NEAA; #11140050; Thermo Fisher Scientific), and 50 μg/mL gentamycin (15750-045; Thermo Fisher Scientific). During culturing, the cells were kept in a humified incubator at +37 °C with 5% CO_2_ and used in passages 3–15.

#### Cell Transfections with PREP Plasmids

All transient cell transfections were made by using the standard transfection protocol of lipofectamine 3000 (L3000001, Thermo Fisher). For cellular thermal shifting assay (CETSA) experiment, PREP-KO HEK-293 cells were seeded to T25 flasks with the density of 1 × 10^6^ cells. One day after the subculture, the medium was removed, and the cells were transfected with hPREP and PREP mutant constructs (Asn483Ala, Leu499Cys, Tyr470Ala, and Ser485Ala; 6 μg of DNA). For WB, PREP-KO HEK-293 cells were plated on six-well plates (400,000 cells per well). On the next day, cells were transfected with PREP and PREP mutant constructs (2.5 μg of DNA). After 24 h transfection, cell media was exchanged to one containing HUP-36 with the concentration of 10 μM.

#### PREP Activity Assay

To determine IC_50_ values against PREP, we used purified recombinant porcine PREP. PREP enzyme was purified according to the protocol described by Venäläinen et al.^26^ In the microplate assay procedure, 10 μL of the enzyme dilution was preincubated with 65 μL of 0.1 M sodium–potassium phosphate buffer (pH 7.0) containing the compounds at different concentrations at 30 °C for 30 min. The reaction was initiated by adding 25 μL of 4 mM Suc-Gly-Pro7-amido-4-methylcoumarin dissolved in 0.1 M sodium–potassium phosphate buffer (pH 7.0), and the mixture was incubated at 30 °C for 60 min. The reaction was terminated by adding 100 μL of 1 M sodium acetate buffer (pH 4.2). Formation of 7-amido-4-methylcoumarin was determined fluorometrically with a microplate fluorescence reader (excitation at 360 nm and emission at 460 nm). The final concentration of the compounds in the assay mixture varied from 100 μM to 1 nM, and the final concentration of the enzyme was approximately 2 nM. The inhibitory activities (percent of control) were plotted against the log concentration of the compound, and the IC_50_ value was determined by nonlinear regression utilizing GraphPad Prism 3.0 software.

In cell cultures, the PREP activity was measured, as in the study of Myöhänen et al.^11^ The cells were homogenized with lysis buffer (50 mM KH_2_PO_4_, 1.5 mM MgCl_2_, 10 mM NaCl, 1 mM EDTA; pH 7.4). Cell homogenates were centrifuged at 16,000*g* for 10 min at +4 °C. The PREP activity was measured from supernatants using Suc-Gly-Pro7-amido-4-methylcoumarin substrate as above. The protein concentration was measured by BCA, and the specific activity was correlated to the protein amount.

#### α-Synuclein Dimerization Assay

To study the effect of compounds on the early phases of αSyn aggregation, αSyn dimerization was assessed by using a protein fragment complementation assay (PCA) that is slightly modified from that by Savolainen et al.^2^ and used by us (Kilpeläinen et al.^18^ and in Pätsi et al).^19^ Briefly, N2A cells were seeded on 96-well plates (Isoplate white wall, PerkinElmer Life Sciences) at the density of 13,000 cells/well and transfected with 25 ng of both αSyn-Gluc1 and αSyn-Gluc2 or 50 ng mock-plasmid as a control by using Lipofectamine 3000 (L3000001; Thermo Fischer Scientific) as the transfection reagent; 48 h post-transfection, cells were incubated for 4 h with the study compounds (10 μM). 0.1% DMSO served as the vehicle control, and proteasomal inhibitor lactacystin (L-1147; AG Scientific, San Diego, CA) at 10 μM served as a positive control for αSyn dimerization. The PCA signal was assessed by injecting 25 μL of native coelenterazine (Nanolight Technology) in phenol red-free DMEM per well. The emitted luminescence was read using a Varioskan LUX multimode microplate reader (Thermo Fisher Scientific). For each experimental condition, four replicate wells were used, and at least three separate experiments for each treatment.

#### Autophagic Flux Assay

To assess the effect of compounds on autophagy, autophagic flux was determined by using HEK-293 cells with stable GFP-LC3B-RFP construct expression. The assay was performed as described by Svarcbahs et al.^3^ and by Kilpeläinen et al.^17^ Briefly, the cells were seeded at a density of 30,000 cells/well on black, clear-bottomed 96-well plates (Costar, Corning) and treated for 24 h with the study compounds 24 h postplating (10 μM; 0.1% DMSO as the vehicle control). 0.5 μM rapamycin, an mTOR inhibitor (BML-A275; Enzo Life Sciences), was used as a positive control for autophagy induction and 20 nM bafilomycin 1A (ML1661) as an autophagy inhibitor. Twenty-four hours after treatment, cells were washed once with warm PBS, and GFP signal was read with Victor2 multilabel counter (PerkinElmer; excitation/emission 485 nm/535 nm). For each experimental condition, four replicate wells were used in each experiment, and at least three independent experiments were performed.

#### ROS Detection Assay

The impact of compounds on ROS production under oxidative stress (OS) was assessed as we have done earlier in the study of Eteläinen et al.^5^ In short, SH-SY5Y cells were plated on a clear bottom black-walled 96-well plate (30,000 cells per well) and incubated overnight. OS was induced treating the cells with a culture medium including 100 μM H_2_O_2_ (H1009; Merck) and 10 mM FeCl_2_ (44939-50G) with or without concurrent treatment compounds for 3 h (10 μM). The cells in the control wells received only fresh cell growth medium during OS induction. Stress-induced ROS production was studied using the DCFDA cellular ROS detection assay kit (ab113851, Abcam) according to the protocol provided with it. ROS proportional fluorescence signal was measured with Victor2 multilabel counter (PerkinElmer; excitation/emission 485 nm/535 nm).

#### Western Blot

WB analysis was used to study protein markers from **HUP-46**/KYP-2047-incubated cell lysates. For WB, the cells were incubated for 4 h with PREP ligands (in autophagic flux assay, together with 20 nM bafilomycin) and thereafter were lysed to RIPA buffer with protease and phosphatase inhibitors (HALT, #78429 and #78420, Thermo Fisher Scientific). Protein concentration was determined by using BCA assay kit (#J63283.QA, Thermo Fisher Scientific). The standard SDS-PAGE protocol was used, and 30 μg of protein per sample was loaded on 4–20% (#4568094, Bio-Rad, CA, USA) stain-free precast gels. The gels were transferred to PVDF membranes (Trans-blot Turbo Midi 0.2 μm, #1704157, Bio-Rad) using a Trans-blot Turbo Transfer System (Bio-Rad). The membranes were blocked with 5% skim milk in TTBS, which was followed by the addition of the primary antibody diluted in 5% skim milk in TTBS and overnight incubation on a swinger at +4 °C. The following primary antibodies were used: Rb LC3B (1:1000, L7543, Sigma-Aldrich), Rb PP2A phospho-T307 (1:500, PA5-36874, Thermo Fisher Scientific), Rb PP2AC (α + β); Clone Y119 (1:2000, ab32141, Abcam), Rb PREP (1:1000, ab58988, Abcam), Ms beta-actin (1:2000, loading control, ab8227, Abcam) Rb Vinculin (1:2000, loading control, ab129002, Abcam).

The following day, the membranes were washed, followed by a 2 h incubation at room temperature with Gt-anti-Rb (#31460, Invitrogen, 1:2000). After incubation, the membranes were washed and incubated with SuperSignal West Pico (#34577) or Femto (#34095) Chemiluminescent Substrate (Thermo Fisher Scientific) for 5 min, and the images were captured with the ChemiDoc XRS+ Gel Imaging System (Bio-Rad) controlled by ImageLab software (version 6.01, Bio-Rad).

To verify that bands were in the linear range of detection, increasing exposure time and automatic detection of saturated pixels in ImageLab software (version 6.01, Bio-Rad) were used. Thereafter, images were converted to 8-bit grayscale format, and the OD (arbitrary units, a.u.) of the bands was measured with ImageJ (histogram area analysis; version 1.53c; NIH). The OD obtained from each band was normalized against the corresponding vinculin band, which was used as the loading control.

#### Cellular Thermal Shift Assay

After the 24 h transfection with PREP mutants (Asn483Ala, Leu499Cys, Tyr470Ala, and Ser485Ala), the cells were exposed to **HUP-46**, KYP-2091, or KYP-2112 (10 μM) in the medium for 2 h. After the exposure, the cells were collected in PBS and aliquoted into seven PCR tubes (100,000 cells/tube). The cells were prewarmed at 37 °C for 3 min, then heated to 37, 47, 50, 53, 56, 63, or 67 °C for 3 min, and subsequently cooled at 25 °C for 3 min using a PCR Mastercycler (T100 Thermal Cycler, Bio-Rad). After the heating, the cells were disrupted with two freeze–thaw cycles by submerging the tubes into liquid nitrogen and subsequently thawed by incubation at 25 °C for 3 min. The aggregated proteins were removed by centrifugation (at 20,000*g* for 20 min at 4 °C), and the soluble fractions were diluted with Laemmli buffer (Bio-Rad, Hercules, CA, USA) and analyzed by Western blot as described above. The nondenaturated protein fractions (%) were calculated by comparing the intensities of temperature-treated cell samples to the corresponding cell samples from 37 °C.

## Supporting Information Available

Synthesis of compounds **2**–**17**, UPLC traces for the tested compounds, molecular modeling experimental data and supplementary results, and biological assay experimental data and supplementary results (PDF)
Molecular formula strings and the associated biological data (CSV)
Docking model of HUP-28 at the new binding site of PREP (PDB)
Docking model of HUP-46 at the active site of PREP (PDB)
Docking model of HUP-46 at the new binding site of PREP (PDB)
Docking model of HUP-55 at the active site of PREP (PDB)
Docking model of HUP-55 at the new binding site of PREP (PDB)

## Supplementary Material

jm3c01993_si_002.csv

jm3c01993_si_003.pdb

jm3c01993_si_004.pdb

jm3c01993_si_005.pdb

jm3c01993_si_006.pdb

jm3c01993_si_007.pdb

## Abbreviations

αSyn: alpha-synuclein
ABPP: activity-based protein profiling
AMC: 7-amino-4-methyl coumarin
CETSA: cellular thermal shift assay
DPP: dipeptidyl peptidase
FAP: fibroblast activating protein
HBTU: hexafluorophosphate benzotriazole tetramethyl uranium
ITC: isothermal titration calorimetry
KO: knockout
LACTA: lactacystin
LC3B: microtubule-associated proteins 1A/1B light chain 3B
MW: microwave
NDD: neurodegenerative disease
PCA: protein-fragment complementation assay
PP2A: protein phosphatase 2A
PPI: protein–protein interaction
PREP: prolyl oligopeptidase
