# Supplementary material for: 5-Aminothiazoles Reveal a New Ligand-Binding Site on Prolyl Oligopeptidase Which is Important for Modulation of Its Protein–Protein Interaction-Derived Functions
Source: J Med Chem. 2024 Mar 28;67(7):5421–36. doi: 10.1021/acs.jmedchem.3c01993 (PMC11394002; doi:10.1021/acs.jmedchem.3c01993)
Supplement: Supplementary file 1 — jm3c01993_si_001.pdf [file jm3c01993_si_001.pdf]

SUPPORTING INFORMATION

5-Aminothiazoles reveal a new ligand binding site on prolyl oligopeptidase which is important for modulation of its protein-protein interaction derived functions

Henri T. Pätsi,[1] Tommi P. Kilpeläinen,[2] Mikael Jumppanen,[1] Johanna Uhari-Väänänen,[2] Pieter Van Wielendaele,[3] Francesca De Lorenzo,[2] Hengjing Cui,[4] Samuli Auno,[2] Janne Saharinen,[1] Erin Seppälä,[5] Nina Sipari,[6] Juha Savinainen,[5] Ingrid De Meester,[3] Anne-Marie Lambeir,[3] Maija Lahtela-Kakkonen,[4] Timo T. Myöhänen,[2,4,7] Erik A. A. Wallén[1,*]

[1] Drug Research Program, Division of Pharmaceutical Chemistry and Technology, Faculty of Pharmacy, University of Helsinki, P.O. Box 56, 00014 Helsinki, Finland

[2] Drug Research Program, Division of Pharmacology and Pharmacotherapy, Faculty of Pharmacy, University of Helsinki, P.O. Box 56, 00014 Helsinki, Finland

[3] Laboratory of Medical Biochemistry, Department of Pharmaceutical Sciences, Faculty of Pharmaceutical, Biomedical and Veterinary Sciences, University of Antwerp, 2610 Wilrijk, Belgium

[4] School of Pharmacy, Faculty of Health Sciences, University of Eastern Finland, Yliopistonranta 1C, 70211 Kuopio, Finland

[5] School of Medicine / Biomedicine, Faculty of Health Sciences, University of Eastern Finland, Yliopistonranta 8, Kuopio 70211, Finland

[6] Viikki Metabolomics Unit, Faculty of Biological and Environmental Sciences, University of Helsinki, Viikinkaari 5 E, 00014 Helsinki, Finland

[7] Division of Pharmacology, Faculty of Medicine, University of Helsinki, P.O.Box 63, 00014 Helsinki, Finland

* Corresponding author: Dr. Erik A. A. Wallén

E-mail: erik.wallen@helsinki.fi

Contents

Chemistry.....S3
General Information.....S3
Synthesis of Compounds.....S3
UPLC-MS Traces and NMR Spectra of Final Compounds.....S7
Molecular Modelling Experimental Data.....S34
Crystal Structure Choice and Preparation.....S34
Identifying potential binding sites.....S34
Molecular Docking.....S34
Molecular Dynamics.....S34
Molecular Modelling Supplementary Results.....S36
Docking to the active site.....S36
Docking to the new binding site.....S36
Conservation of the new binding site.....S37
Molecular dynamics simulation interactions.....S38
Covalent docking to the new binding site.....S40
Biological Experimental Data.....S41
Reagents.....S41
DNA Constructs.....S41
Mouse primary cortical neuron cultures.....S41
Cell Viability Assay.....S41
Close Relative Enzyme Specificity Assay.....S42
Activity-Based Protein Profiling.....S43
Recombinant Human PREP Mutant Protein Production and Purification.....S43
Isothermal Titration Calorimetry.....S44
LC-MS detection of HUP-46 in the mouse brain.....S45
Statistical Analysis.....S45
Biological Supplementary Results.....S46
Cell Viability.....S47
Close relative enzyme specificity assay.....S47
ITC.....S49
Brain Penetration.....S52
References.....S53

Chemistry

General Information

Unless otherwise specified, all reagents and solvents were obtained from commercial suppliers and used without purification. Microwave reactions were performed with fixed hold time in capped microwave vials using a Biotage Initiator+ (Biotage). Completion of reactions and purifications were monitored with TLC, which was performed on 60 F_{254} silica gel plates, using UV light (254 and 366 nm) and ninhydrin or iodine staining to detect products. Flash chromatography was performed manually with silica gel (230-400 µm mesh) or using a Biotage Isolera One (Biotage) with silica gel 60 (40-63 µm mesh), unless otherwise specified. ^{1}H and ^{13}C NMR spectra were recorded at 400 MHz and 101 MHz, respectively, using an Ascend 400 (Bruker). $CDCl_3$ was used as the NMR solvent unless otherwise specified. Chemical shifts (δ) are reported in parts per million (ppm) with TMS or solvent residual peaks as reference. Exact mass and purity of the tested compounds were analyzed with LC-MS, using a Waters Aquity UPLC system (Waters) and a Waters Synapt G2 HDMS mass spectrometer (Waters) via an ESI ion source in positive mode. The purity of all tested final compounds was 95 % or higher, except compound **8**, which had a purity of 94 %.

Synthesis of Compounds

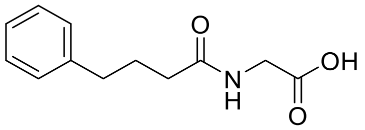

(4-Phenylbutanoyl)glycine (14l). 4-Phenylbutyric acid (300 mg, 1.8 mmol) was heated to 70 °C. $SOCl_2$ (0.20 ml, 2.7 mmol) was added dropwise. The mixtu<re was stirred at 70 °C for 1 h followed by the evaporation of the remaining $SOCl_2$ to provide the acid chloride intermediate (quantitative), which was used without further purification. The acid chloride in Et_2O (1 ml) was added to a solution of glycine (151 mg, 2.0 mmol) in Et_2O (5 ml) and Na_2CO_3 (5 ml, 10 % (m/V)). The mixture was stirred vigorously for 22 h before separating the phases. The aqueous phase was washed with Et_2O, acidified with 1 M HCl, and extracted with EtOAc. The combined organic phases were dried over anhydrous Na_2SO_4, filtered, and evaporated to provide the crude product as an orange solid (319 mg 79 %), which was used without further purification. Unreacted starting material, which is not reported in the NMR spectra, was also identified at a 7:3 ratio compared to the product. ^{1}H NMR (Methanol-d_4) δ 7.33 – 7.24 (m, 2H), 7.24 – 7.13 (m, 3H), 3.91 (s, 2H), 2.71 – 2.60 (m, 2H), 2.35 – 2.23 (m, 2H), 2.00 – 1.85 (m, 2H). ^{13}C NMR (Methanol-d_4) δ 176.35, 173.05, 143.02, 129.51, 129.36, 126.95, 126.90, 41.73, 36.14 (2 peaks), 28.65.

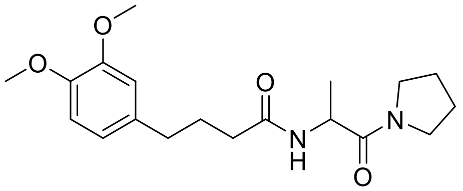

Method A: Synthesis of 4-(3,4-Dimethoxyphenyl)-*N*-(1-oxo-1-(pyrrolidin-1-yl)propan-2-yl)butanamide (15a). A mixture of 4-(3,4-Dimethoxyphenyl)butanoic acid (151 mg, 0.67 mmol), HBTU (276 mg, 0.73 mmol), DIPEA (0.29 ml, 1.68 mmol), and L-alanine pyrrolidide hydrochloride (100 mg, 0.56 mmol) in anhydrous DMF (4 ml) was stirred at room temperature for 16 h. The reaction mixture was diluted with diethyl ether and the organic phase was washed with water. The aqueous phase was extracted with diethyl ether and EtOAc and the combined organic phase was dried dried over anhydrous Na_2SO_4 filtered, and evaporated to provide the crude product, which

after flash chromatography (hexane/EtOAc 9:1 → EtOAc) yielded **15a** as a white solid (87 mg, 45 %). Unreacted starting material, which is not reported in the NMR spectra, was also identified at a 1:2 ratio compared to the product. ^{1}H NMR δ 6.85 – 6.64 (m, 3H), 4.82 – 4.67 (m, 1H), 3.86 (d, *J* = 6.1 Hz, 6H), 3.64 (dt, *J* = 10.1, 6.6 Hz, 1H), 3.56 – 3.39 (m, 3H), 2.63 – 2.56 (m, 2H), 2.37 (t, *J* = 7.5 Hz, 1H), 2.22 (dd, *J* = 8.3, 6.8 Hz, 2H), 2.04 – 1.83 (m, 5H), 1.33 (d, *J* = 6.8 Hz, 3H). ^{13}C NMR δ 172.27, 171.24, 148.98, 147.37, 134.34, 120.45, 111.87, 111.35, 56.06, 55.95, 46.85, 46.57, 46.24, 35.99, 35.01, 27.49, 26.16, 24.25, 18.53.

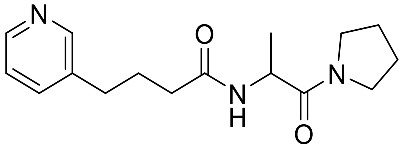

***N*-(1-Oxo-1-(pyrrolidin-1-yl)propan-2-yl)-4-(pyridin-3-yl)butanamide (15e).** Synthesized according to method A using 4-(pyridin-3-yl)butanoic acid (100 mg, 0.61 mmol). The crude product was obtained, which after flash chromatography (EtOAc → EtOAc/MeOH 4:1) yielded **15e** (67 mg, 41 %). ^{1}H NMR δ 8.43 – 8.31 (m, 2H), 7.51 – 7.41 (m, 1H), 7.15 (ddd, *J* = 7.8, 4.9, 0.9 Hz, 1H), 6.70 (d, *J* = 7.6 Hz, 1H), 4.65 (p, *J* = 6.9 Hz, 1H), 3.56 (dt, *J* = 10.1, 6.6 Hz, 1H), 3.48 – 3.29 (m, 3H), 2.63 – 2.53 (m, 2H), 2.22 – 2.11 (m, 2H), 1.97 – 1.73 (m, 6H), 1.25 (d, *J* = 6.8 Hz, 3H). ^{13}C NMR δ 171.61, 171.00, 149.65, 147.20, 137.11, 136.32, 123.53, 46.79, 46.44, 46.12, 35.53, 32.34, 26.78, 26.07, 24.15, 18.37.

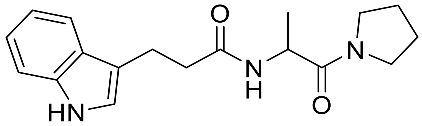

3-(1*H*-Indol-3-yl)-*N*-(1-oxo-1-(pyrrolidin-1-yl)propan-2-yl)propanamide (15g). Synthesized according to method A using 3-indolepropionic acid (127 mg, 0.67 mmol). The crude product was obtained, which after flash chromatography (hexane/EtOAc 9:1 → EtOAc) yielded **15g** (100 mg 57 %). ^{1}H NMR δ 8.31 (s, 1H), 7.61 – 7.56 (m, 1H), 7.33 (dt, *J* = 8.0, 1.0 Hz, 1H), 7.16 (ddd, *J* = 8.2, 7.0, 1.2 Hz, 1H), 7.09 (ddd, *J* = 8.0, 7.0, 1.1 Hz, 1H), 7.01 – 6.97 (m, 1H), 6.57 (d, *J* = 7.6 Hz, 1H), 4.73 – 4.64 (m, 1H), 3.54 (dt, *J* = 10.1, 6.6 Hz, 1H), 3.49 – 3.32 (m, 3H), 3.13 – 3.06 (m, 2H), 2.64 – 2.55 (m, 2H), 1.97 – 1.88 (m, 2H), 1.87 – 1.79 (m, 2H), 1.24 (d, *J* = 6.8 Hz, 3H). ^{13}C NMR δ 172.14, 171.03, 136.44, 127.34, 122.00, 121.72, 119.29, 118.86, 115.04, 111.25, 46.86, 46.46, 46.15, 37.36, 26.09, 24.19, 21.31, 18.38. HRMS (ESI-QTOF) *m/z*: [M + H]$^{+}$ Calcd for $C_{18}H_{24}N_{3}O_{2}$ 314.1869; Found 314.1870.

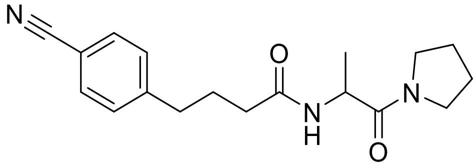

Method B: 4-(4-Cyanophenyl)-N-(1-oxo-1-(pyrrolidin-1-yl)propan-2-yl)butanamide (15b). A mixture of 4-(4-cyanophenyl)butanoic acid (30 mg, 0.16 mmol), HBTU (379 mg, 0.20 mmol), DIPEA (0.08 ml, 0.47 mmol) and L-alanine pyrrolidide hydrochloride (28 mg, 0.16 mmol) in anhydrous DCM (2 ml) was stirred at room temperature for 16 h. The solvent was evaporated to obtain the crude product, which after flash chromatography (EtOAc → EtOAc/MeOH 4:1) yielded **15b** (quantitative). DIPEA and the HBTU urea byproduct were also identified, which are not reported in the NMR spectra. ^{1}H NMR δ 7.62 – 7.54 (m, 2H), 7.33 – 7.26 (m, 2H), 6.62 (d, *J* = 7.5 Hz, 1H), 4.69 (p, *J* = 6.9 Hz, 1H), 3.62 (dt, *J* = 10.2, 6.6 Hz, 1H), 3.56 – 3.38 (m, 3H), 2.75 – 2.67 (m, 2H), 2.27 – 2.19 (m, 2H), 2.04 – 1.82 (m, 6H), 1.33 (d, *J* = 6.8 Hz, 3H). ^{13}C NMR δ 171.71, 170.98, 147.43, 132.32, 129.43, 119.18, 109.90, 46.99, 46.53, 46.25, 35.62, 35.42, 26.67, 26.12, 24.18, 18.31. HRMS (ESI-QTOF) *m/z*: [M + H]$^{+}$ Calcd for $C_{18}H_{24}N_{3}O_{2}$ 314.1869; Found 314.1863.

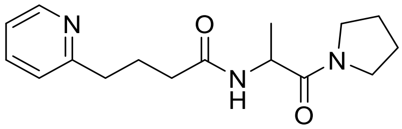

N-(1-Oxo-1-(pyrrolidin-1-yl)propan-2-yl)-4-(pyridin-2-yl)butanamide (15d). Synthesized according to method B using 4-(pyridin-2-yl)butanoic acid (93 mg, 0.16 mmol). The crude product was obtained, which after flash chromatography (EtOAc → EtOAc/MeOH 4:1) yielded **15d** (quantitative). Unreacted starting material, which is not reported in the NMR spectra, was also identified at a 1:5 ratio compared to the product. ^{1}H NMR δ 8.46 – 8.35 (m, 1H), 7.63 – 7.55 (m, 1H), 7.17 – 7.13 (m, 1H), 7.10 (ddd, J = 7.6, 5.0, 1.2 Hz, 1H), 6.92 (d, J = 7.4 Hz, 1H), 4.62 (p, J = 6.9 Hz, 1H), 3.56 (dt, J = 10.2, 6.6 Hz, 1H), 3.45 – 3.29 (m, 3H), 2.77 (t, J = 7.5 Hz, 2H), 2.17 (td, J = 7.2, 3.0 Hz, 2H), 2.03 – 1.75 (m, 6H), 1.25 (d, J = 6.9 Hz, 3H). ^{13}C NMR δ 172.00, 171.07, 160.70, 148.21, 137.54, 123.46, 121.62, 46.85, 46.38, 46.10, 36.56, 35.26, 25.98, 25.58, 24.05, 18.04.

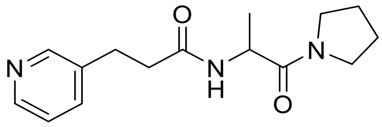

N-(1-Oxo-1-(pyrrolidin-1-yl)propan-2-yl)-3-(pyridin-3-yl)propanamide (15f). Synthesized according to method B using 3-pyridinepropionic acid (93 mg, 0.16 mmol). The crude product was obtained, which after flash chromatography (hexane/EtOAc 9:1 → EtOAc) yielded **15f** (147 mg, 96 %). ^{1}H NMR δ 8.42 – 8.39 (m, 1H), 8.37 (dd, J = 4.9, 1.7 Hz, 1H), 7.56 – 7.46 (m, 1H), 7.16 (ddd, J = 7.8, 4.9, 0.9 Hz, 1H), 6.55 (d, J = 7.5 Hz, 1H), 4.62 (p, J = 6.9 Hz, 1H), 3.52 (dt, J = 10.1, 6.6 Hz, 1H), 3.47 – 3.29 (m, 3H), 2.90 (td, J = 7.6, 2.9 Hz, 2H), 2.48 – 2.41 (m, 2H), 1.96 – 1.86 (m, 2H), 1.86 – 1.75 (m, 2H), 1.21 (d, J = 6.8 Hz, 3H). ^{13}C NMR δ 170.87, 170.59, 149.58, 147.43, 136.61, 136.55, 123.68, 46.98, 46.51, 46.22, 37.58, 28.69, 26.15, 24.22, 18.43. HRMS (ESI-QTOF) m/z: $[M + H]^+$ Calcd for $C_{15}H_{22}N_3O_2$ 276.1712; Found 276.1712.

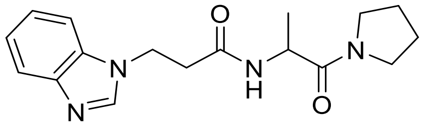

3-(1H-Benzo[d]imidazol-1-yl)-N-(1-oxo-1-(pyrrolidin-1-yl)propan-2-yl)propanamide (15h). Synthesized according to method B using 3-benzimidazol-1-yl-propionic acid (200 mg, 1.05 mmol). The crude product was obtained, which after flash chromatography, using an amine functionalized column (EtOAc → EtOAc/MeOH 9:1), yielded **15h** as a white solid (316 mg, 95 %). ^{1}H NMR δ 7.96 (s, 1H), 7.82 – 7.74 (m, 1H), 7.45 – 7.38 (m, 1H), 7.28 (tt, J = 7.3, 5.7 Hz, 2H), 6.91 (d, J = 7.5 Hz, 1H), 4.65 (p, J = 6.9 Hz, 1H), 4.54 (td, J = 6.4, 3.1 Hz, 2H), 3.54 (dt, J = 10.1, 6.7 Hz, 1H), 3.44 – 3.28 (m, 3H), 2.75 (t, J = 6.5 Hz, 2H), 2.03 – 1.91 (m, 2H), 1.91 – 1.76 (m, 2H), 1.21 (d, J = 6.8 Hz, 3H). ^{13}C NMR δ 170.54, 168.65, 143.89, 143.62, 133.56, 123.01, 122.21, 120.47, 109.54, 47.02, 46.45, 46.16, 40.75, 36.20, 26.08, 24.14, 18.25.

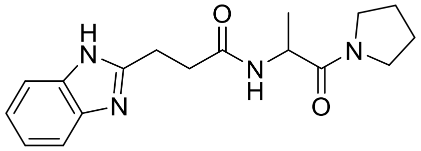

3-(1H-Benzo[d]imidazol-2-yl)-N-(1-oxo-1-(pyrrolidin-1-yl)propan-2-yl)propanamide (15i). Synthesized according to method B using 2-benzimidazolepropionic acid (106 mg, 0.16 mmol). The crude product was obtained, which after flash chromatography (hexane/EtOAc 9:1 → EtOAc) yielded **15i** (67 mg, 38 %). Unreacted starting material, which is not reported in the NMR spectra, was also identified at a 2:5 ratio compared to the product. ^{1}H NMR δ 12.02 (s, 1H), 7.45 (dd, J = 6.1, 3.2 Hz, 2H), 7.14 (dd, J = 6.1, 3.1 Hz, 2H), 5.22 (s, 1H), 4.55 (p, J = 6.9 Hz, 1H), 3.64 – 3.50 (m, 1H),

3.48 – 3.29 (m, 3H), 3.27 – 3.17 (m, 2H), 2.70 – 2.62 (m, 2H), 1.94 – 1.84 (m, 2H), 1.84 – 1.73 (m, 2H), 1.25 (d, *J* = 6.9 Hz, 3H). ^{13}C NMR δ 171.92, 171.27, 153.83, 136.84, 123.06, 114.62, 47.70, 46.49, 46.44, 33.53, 26.13, 24.60, 24.11, 17.39. HRMS (ESI-QTOF) *m/z*: [M + H]$^+$ Calcd for $C_{17}H_{23}N_4O_2$ 315.1821; Found 315.1822.

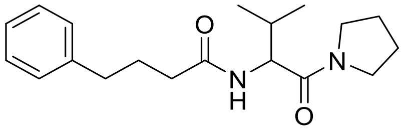

N-(3-Methyl-1-oxo-1-(pyrrolidin-1-yl)butan-2-yl)-4-phenylbutanamide (15j). Synthesized according to method B using 4-phenylbutanoic acid (96 mg, 0.59 mmol) with (2*S*)-2-amino-3-methyl-1-(pyrrolidin-1-yl)butan-1-one (100 mg, 0.59 mmol) replacing the amine. The crude product was obtained, which after flash chromatography (EtOAc → EtOAc/MeOH 4:1) yielded **15j** (163 mg, 96 %). ^{1}H NMR δ 7.25 – 7.15 (m, 2H), 7.15 – 7.05 (m, 3H), 6.30 (d, *J* = 9.0 Hz, 1H), 4.52 (dd, *J* = 9.0, 7.1 Hz, 1H), 3.66 (dt, *J* = 10.2, 6.5 Hz, 1H), 3.46 – 3.29 (m, 3H), 2.63 – 2.53 (m, 2H), 2.19 – 2.13 (m, 2H), 2.03 – 1.70 (m, 7H), 0.87 (dd, *J* = 9.0, 6.7 Hz, 6H). ^{13}C NMR δ 172.86, 170.53, 141.62, 128.57, 128.46, 126.01, 55.84, 46.93, 45.97, 35.97, 35.36, 31.34, 27.36, 26.07, 24.27, 19.60, 18.04. HRMS (ESI-QTOF) *m/z*: [M + H]$^+$ Calcd for $C_{19}H_{29}N_2O_2$ 317.2229; Found 317.2229.

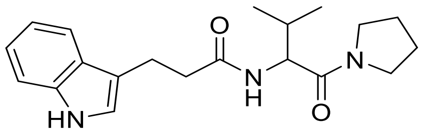

3-(1*H*-Indol-3-yl)-*N*-(3-methyl-1-oxo-1-(pyrrolidin-1-yl)butan-2-yl)propanamide (15k). Synthesized according to method B using 3-indolepropionic acid (56 mg, 0.23 mmol) with (2*S*)-2-amino-3-methyl-1-(pyrrolidin-1-yl)butan-1-one (100 mg, 0.59 mmol) replacing the amine. The crude product was obtained, which after flash chromatography (hexane/EtOAc 9:1 → EtOAc) yielded **15k** (quantitative). ^{1}H NMR δ 8.15 (s, 1H), 7.52 (dp, *J* = 7.8, 0.8 Hz, 1H), 7.29 – 7.23 (m, 1H), 7.10 (ddd, *J* = 8.2, 7.0, 1.2 Hz, 1H), 7.02 (ddd, *J* = 8.0, 7.0, 1.1 Hz, 1H), 6.93 (d, *J* = 2.3 Hz, 1H), 6.20 (d, *J* = 9.0 Hz, 1H), 4.50 (dd, *J* = 9.0, 7.0 Hz, 1H), 3.62 (dt, *J* = 10.2, 6.5 Hz, 1H), 3.44 – 3.26 (m, 3H), 3.11 – 2.98 (m, 2H), 2.60 – 2.51 (m, 2H), 1.98 – 1.68 (m, 5H), 0.82 (d, *J* = 6.8 Hz, 3H), 0.72 (d, *J* = 6.7 Hz, 3H). ^{13}C NMR δ 172.71, 170.50, 136.48, 127.31, 122.05, 121.72, 119.34, 118.87, 115.03, 111.25, 55.85, 46.93, 45.97, 37.33, 31.37, 26.08, 24.29, 21.39, 19.54, 17.87.

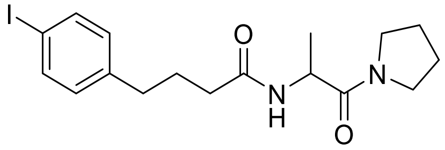

4-(*p*-Iodophenyl)-*N*-(1-oxo-1-(pyrrolidin-1-yl)propan-2-yl)butanamide (15c). 4-(*p*-Iodophenyl)butyric acid (0.76 mg, 0.26 mmol) was heated to 70 °C. $SOCl_2$ (0.03 ml, 0.41 mmol) was added dropwise. The mixture was stirred at 70 °C for 1 h followed by the evaporation of the remaining $SOCl_2$ to provide the acid chloride intermediate (80 mg, 0.26 mmol, 99 %), which was used without further purification. The acid chloride in DCM (1 mL) was added to a solution of L-alanine pyrrolidide hydrochloride (46 mg, 0.26 mmol) and Et_3N (0.07 ml, 0.52 mmol) in DCM (3 mL). The mixture was stirred at room temperature for 19 h. The organic phase was washed with H_2O, dried over anhydrous Na_2SO_4, filtered, and evaporated to provide the crude product as a colourless sap, which after flash chromatography (EtOAc → EtOAc/MeOH 9:1) yielded **15c** as a colourless sap (94 mg, 88 %). ^{1}H NMR δ 7.60 – 7.44 (m, 2H), 6.95 – 6.77 (m, 2H), 6.44 (d, *J* = 7.5 Hz, 1H), 4.63 (p, *J* = 6.9 Hz, 1H), 3.62 – 3.22 (m, 4H), 2.51 (t, *J* = 7.6 Hz, 2H), 2.16 – 2.08 (m, 2H), 1.98 – 1.75 (m, 6H), 1.25 (d, *J* = 6.8 Hz, 3H). ^{13}C NMR δ 171.71, 170.99, 141.31, 137.53, 130.77, 91.11, 46.87, 46.50, 46.18, 35.82, 34.84, 26.99, 26.17, 24.25, 18.61.

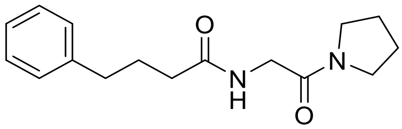

N-(2-Oxo-2-(pyrrolidin-1-yl)ethyl)-4-phenylbutanamide (15l). Pivaloyl chloride (0.16 ml, 1.30 mmol) was added to a solution of **14l** (288 mg, 1.30 mmol) and Et_3N (0.20 ml, 1.43 mmol) in anhydrous DCM (15 ml) at 0 °C. The mixture was stirred at 0 °C for 1 h. Et_3N (0.20 ml, 1.43 mmol) and pyrrolidine (0.10 ml, 1.43 mmol) were added and the mixture was left to stir at room temperature for 3 h. The organic phase was washed with a 20 % aqueous solution of citric acid, a saturated solution of $NaHCO_3$, and brine, dried over anhydrous Na_2SO_4, filtered, and evaporated to provide the crude product as an orange oil, which after flash chromatography (EtOAc/MeOH 99:1 → 19:1) yielded **15l** as a white solid (109 mg, 31 %). Poor yield probably due to poor conversion in previous reaction. ^{1}H NMR δ 7.26 – 7.15 (m, 2H), 7.15 – 7.06 (m, 3H), 6.50 (s, 1H), 3.90 (d, J = 4.1 Hz, 2H), 3.42 (t, J = 6.9 Hz, 2H), 3.31 (t, J = 6.8 Hz, 2H), 2.59 (t, J = 7.6 Hz, 2H), 2.26 – 2.14 (m, 2H), 1.99 – 1.86 (m, 4H), 1.86 – 1.75 (m, 2H). ^{13}C NMR δ 172.84, 166.55, 141.61, 128.59, 128.46, 126.01, 46.07, 45.53, 42.17, 35.77, 35.35, 27.26, 26.01, 24.24.

UPLC-MS Traces and NMR Spectra of Final Compounds

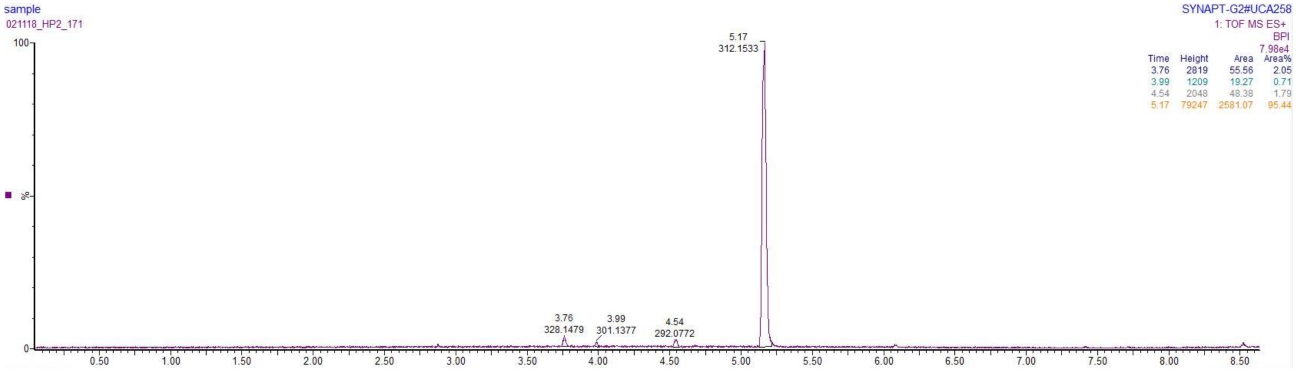

Figure S1. UPLC-MS trace from compound **HUP-46**.

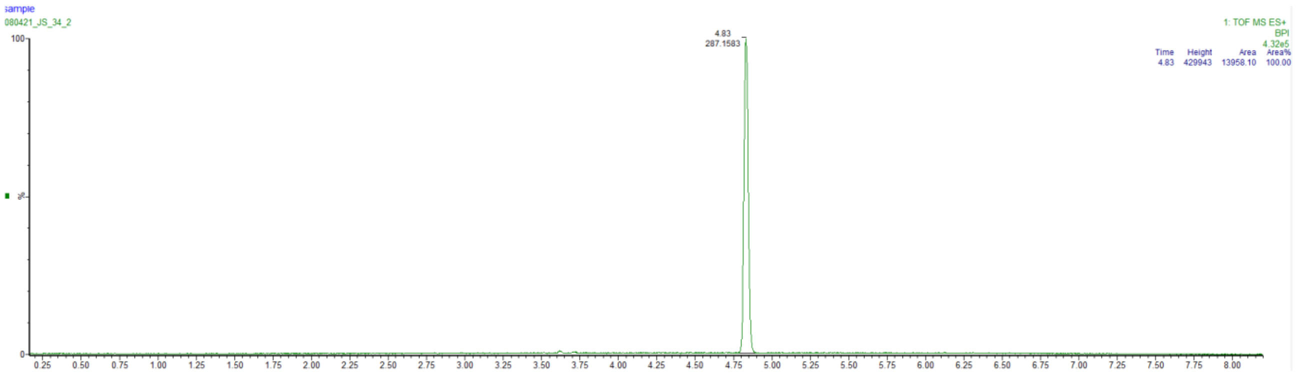

Figure S2. UPLC-MS trace from compound **7**.

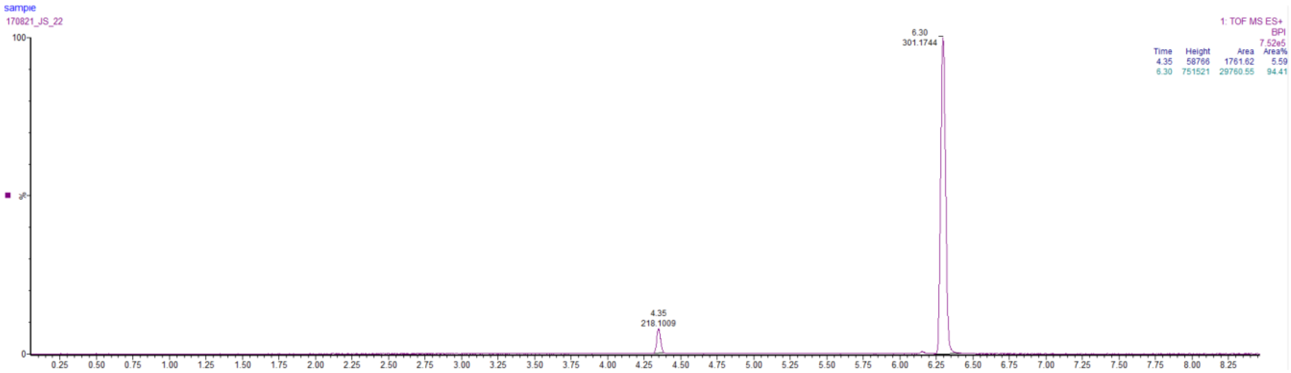

Figure S3. UPLC-MS trace from compound **8**.

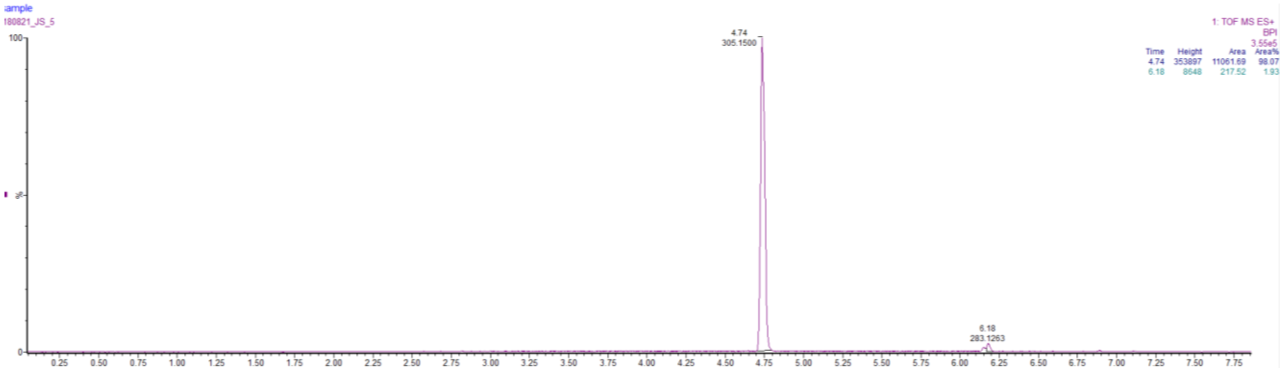

Figure S4. UPLC-MS trace from compound **9**.

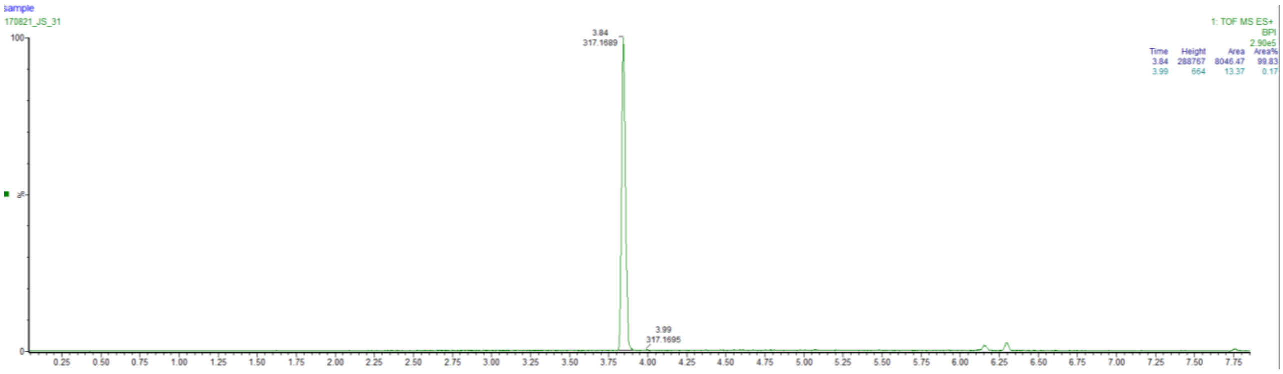

Figure S5. UPLC-MS trace from compound **10**.

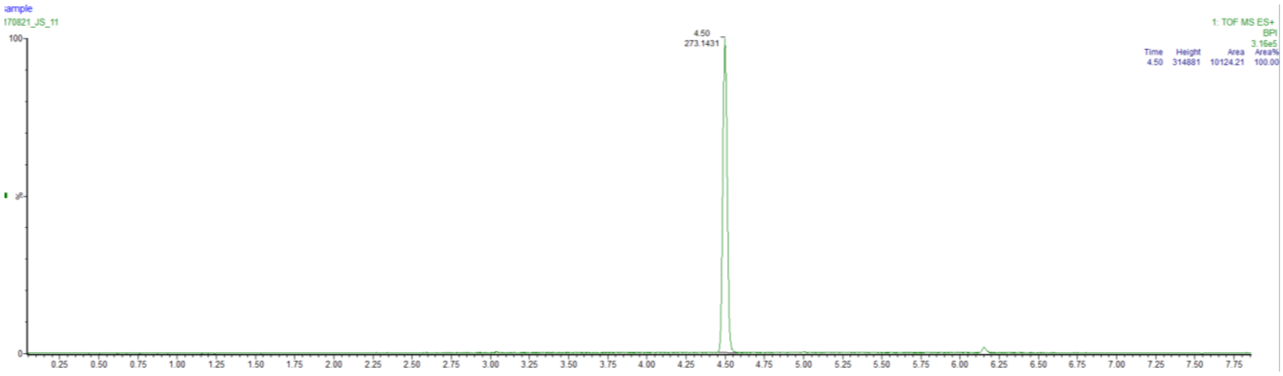

Figure S6. UPLC-MS trace from compound **12**.

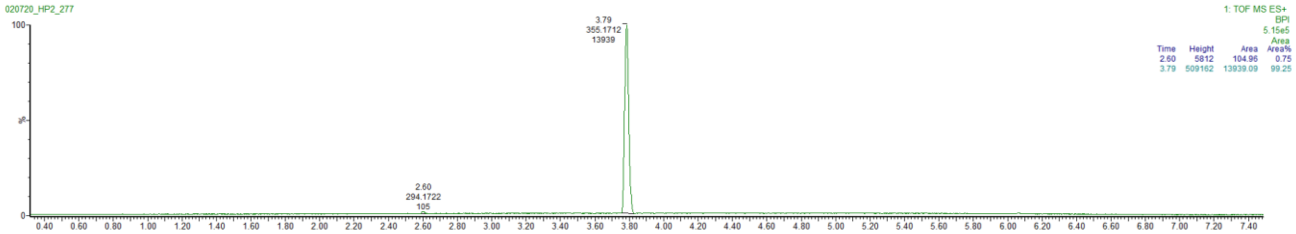

Figure S7. UPLC-MS trace from compound **13**.

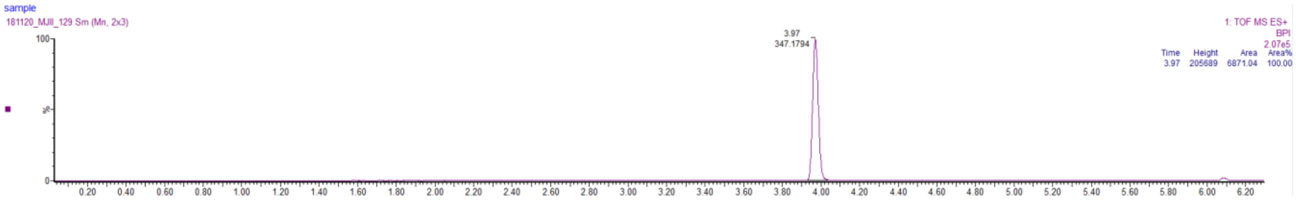

Figure S8. UPLC-MS trace from compound **16a**.

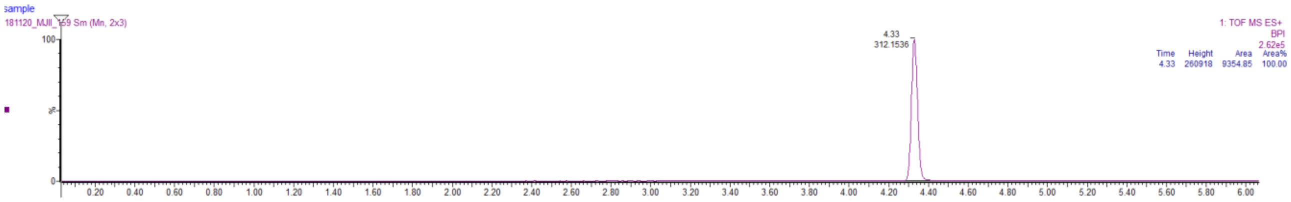

Figure S9. UPLC-MS trace from compound **16b**.

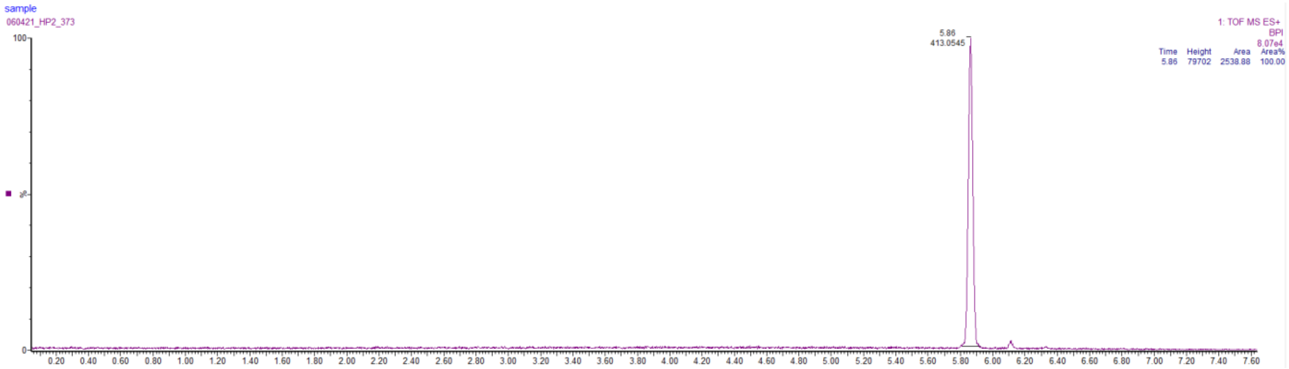

Figure S10. UPLC-MS trace from compound **16c**.

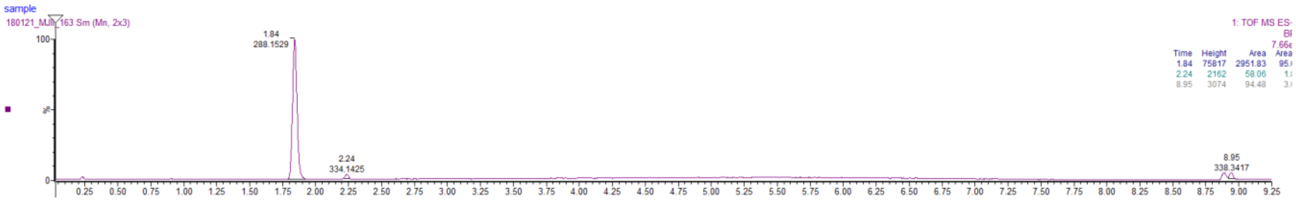

Figure S11. UPLC-MS trace from compound **16d**.

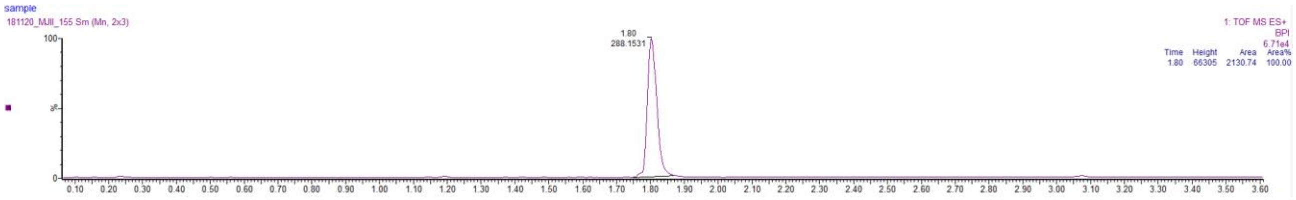

Figure S12. UPLC-MS trace from compound **16e**.

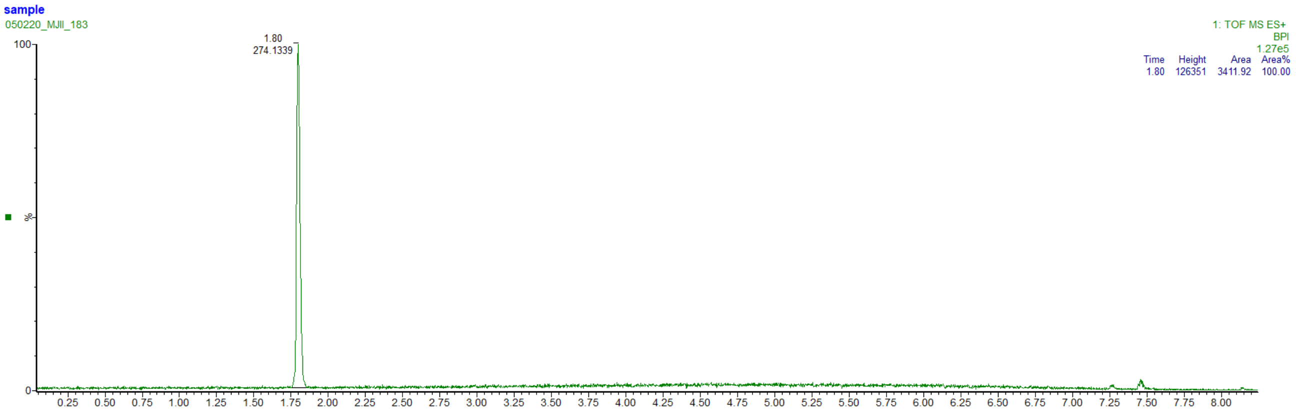

Figure S13. UPLC-MS trace from compound **16f**.

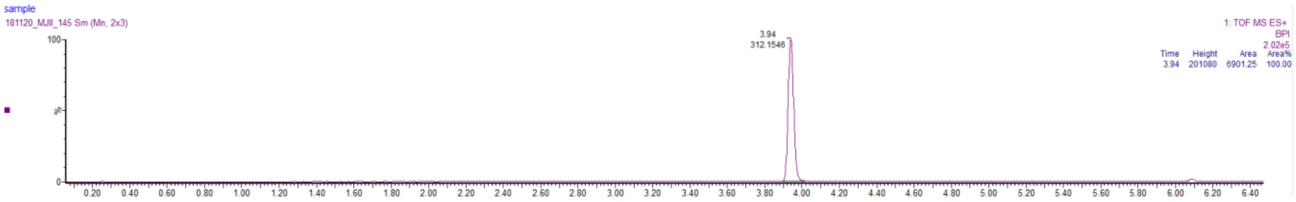

Figure S14. UPLC-MS trace from compound **16g**.

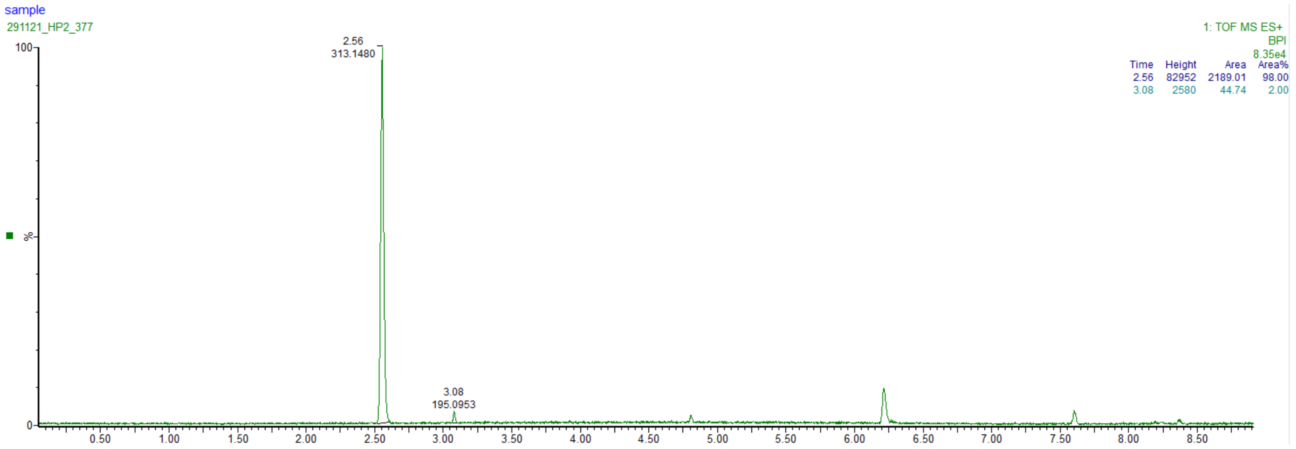

Figure S15. UPLC-MS trace from compound **16h**.

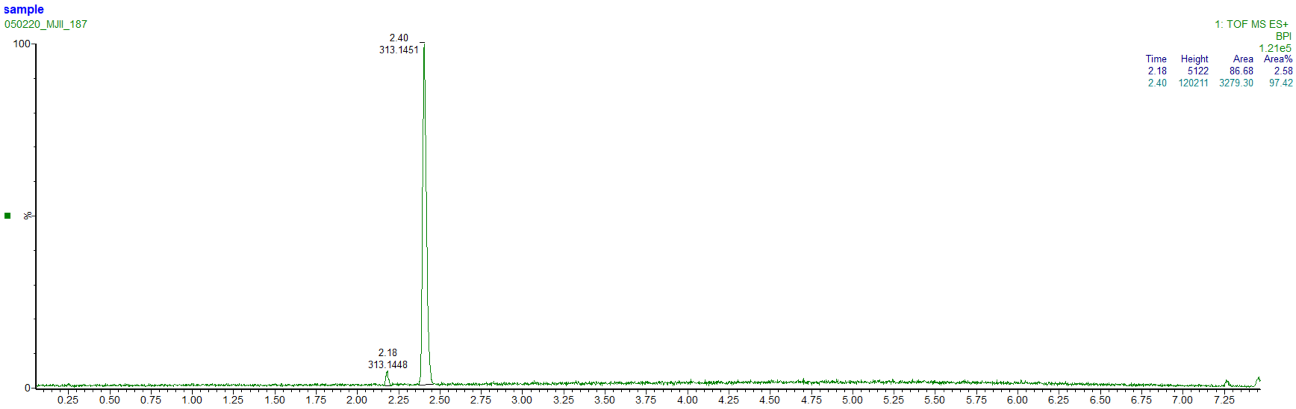

Figure S16. UPLC-MS trace from compound **16i**.

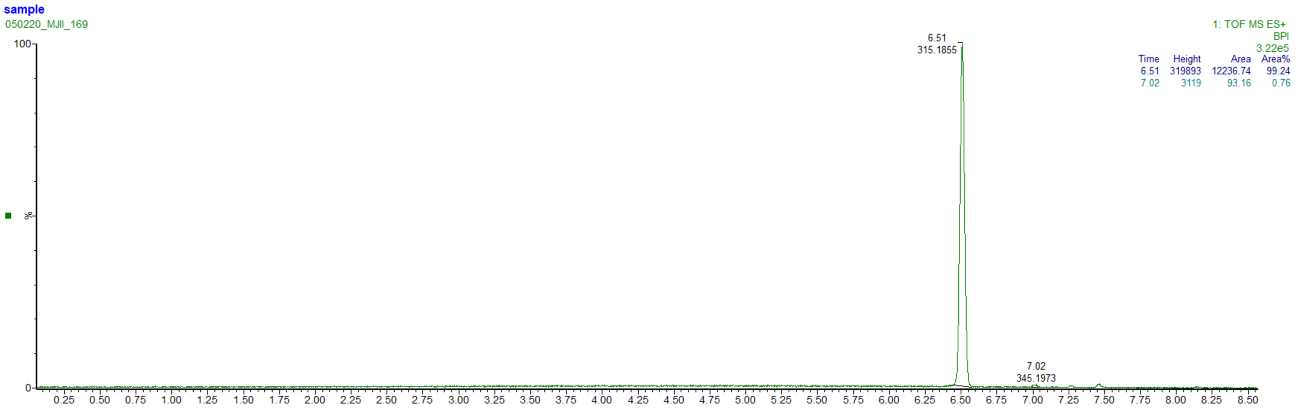

Figure S17. UPLC-MS trace from compound **16j**.

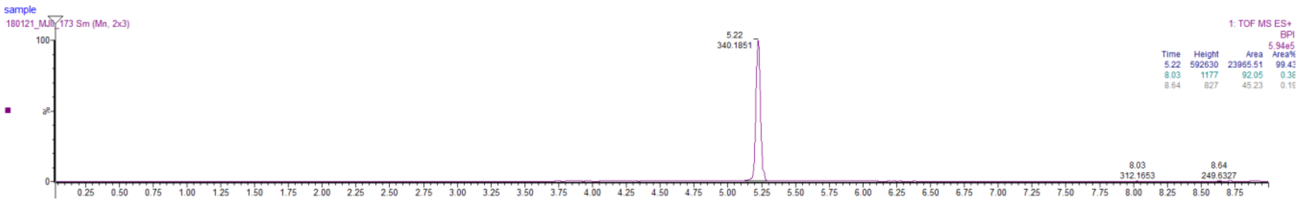

Figure S18. UPLC-MS trace from compound **16k**.

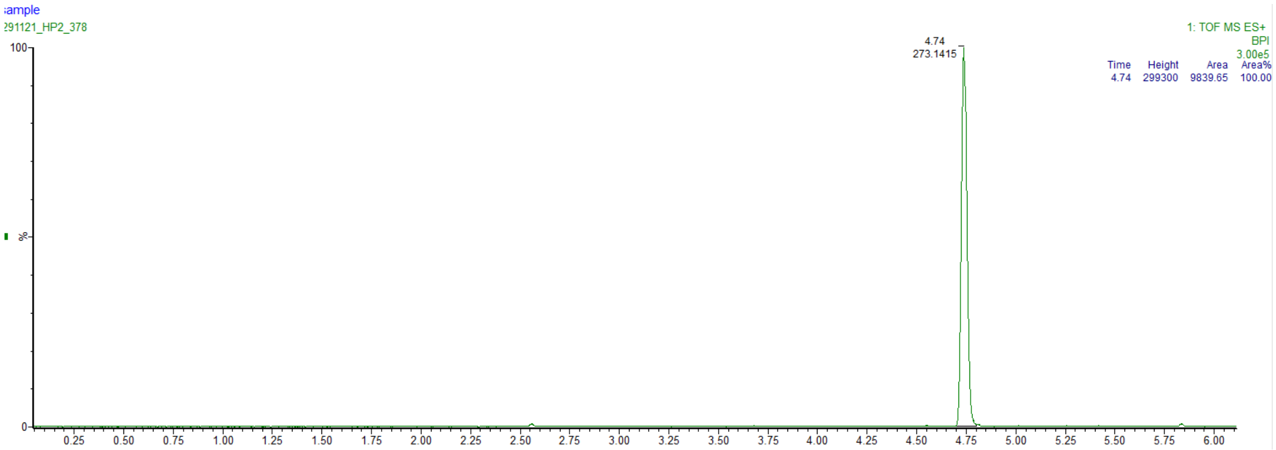

Figure S19. UPLC-MS trace from compound **16l**.

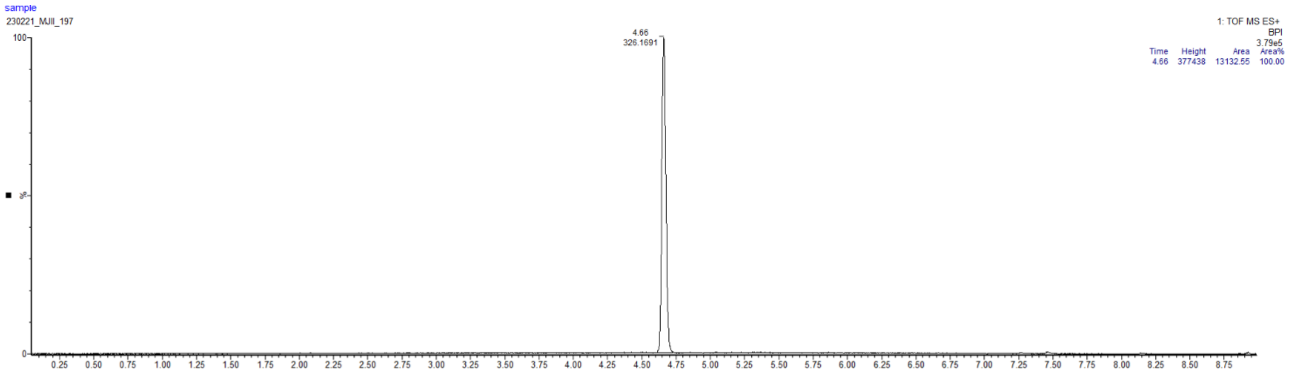

Figure S20. UPLC-MS trace from compound **17**.

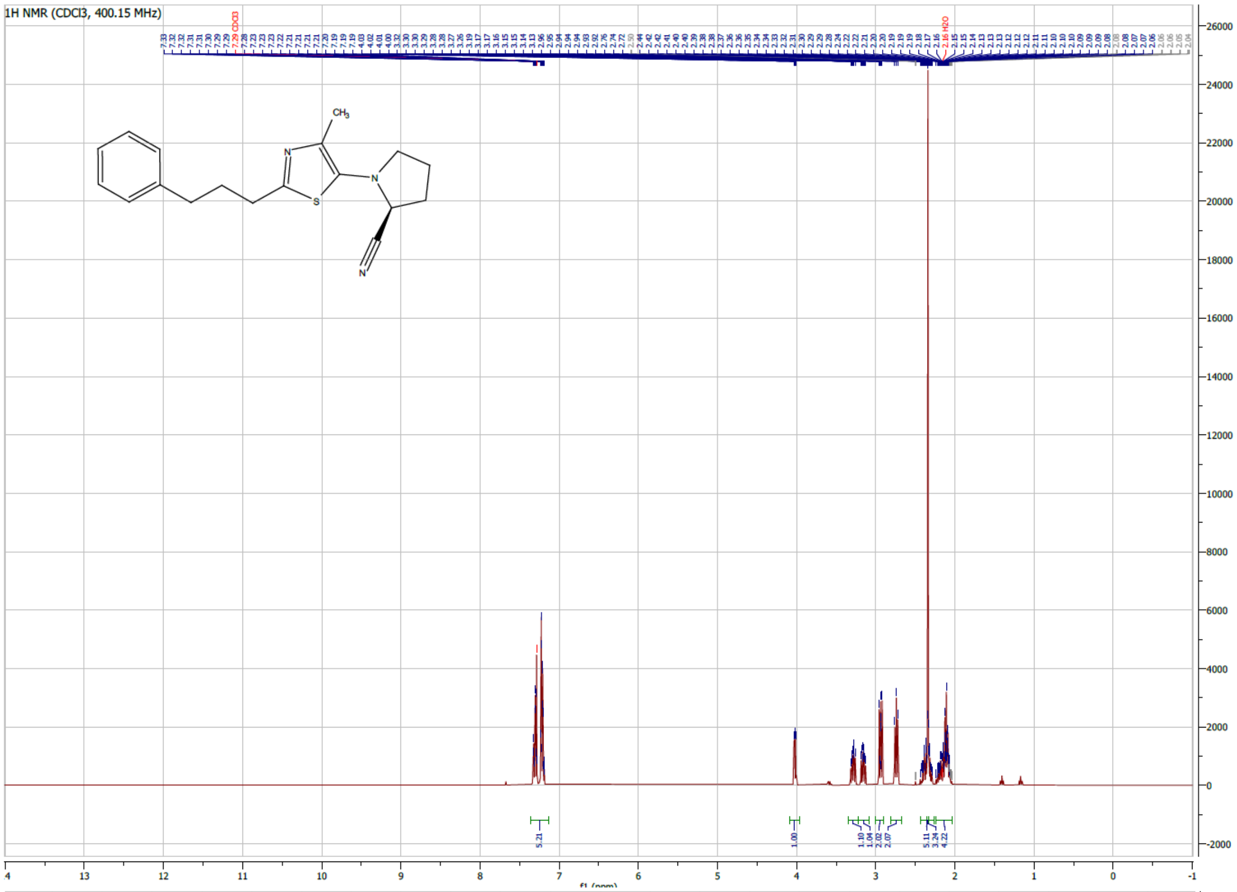

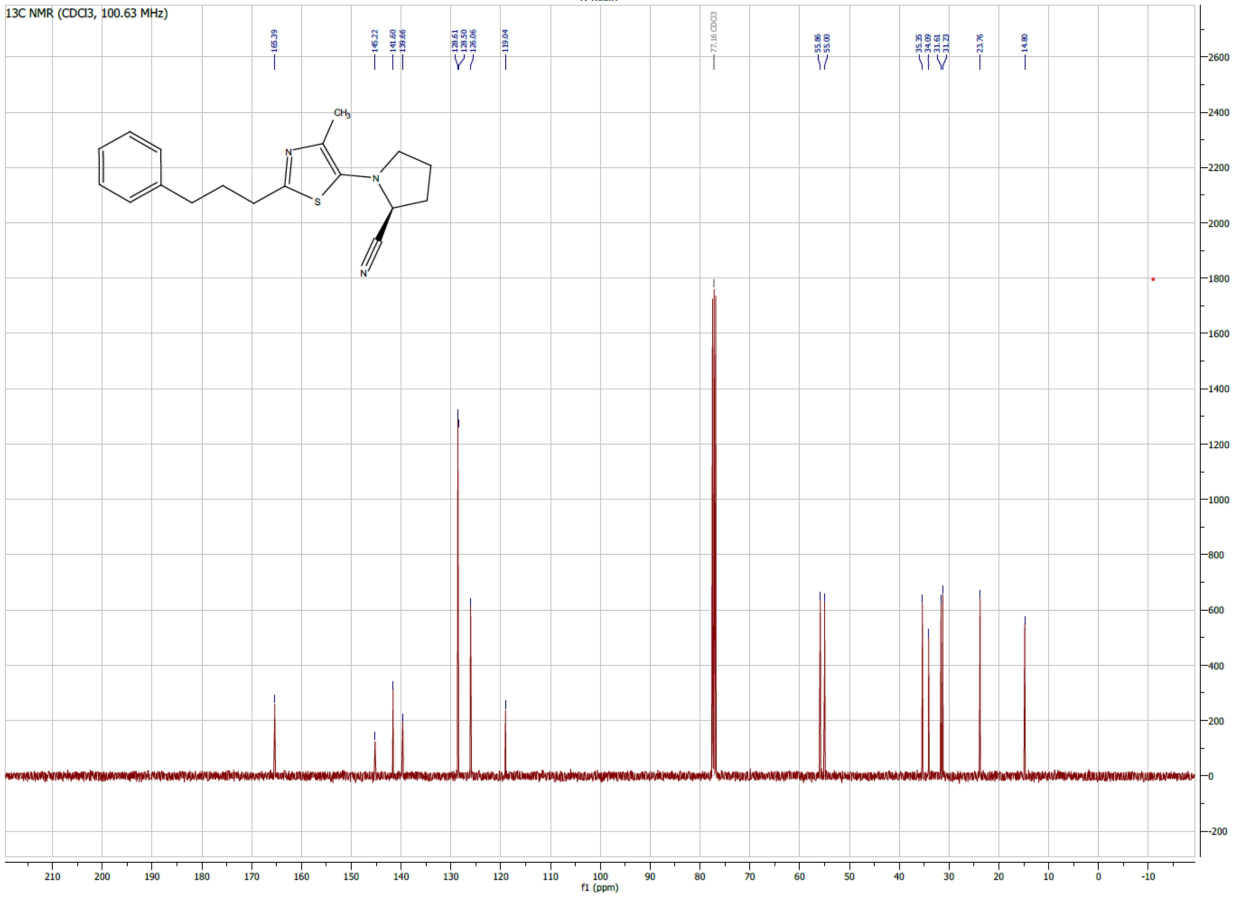

Figure S21. ^{1}H and ^{13}C NMR spectra of HUP-46.

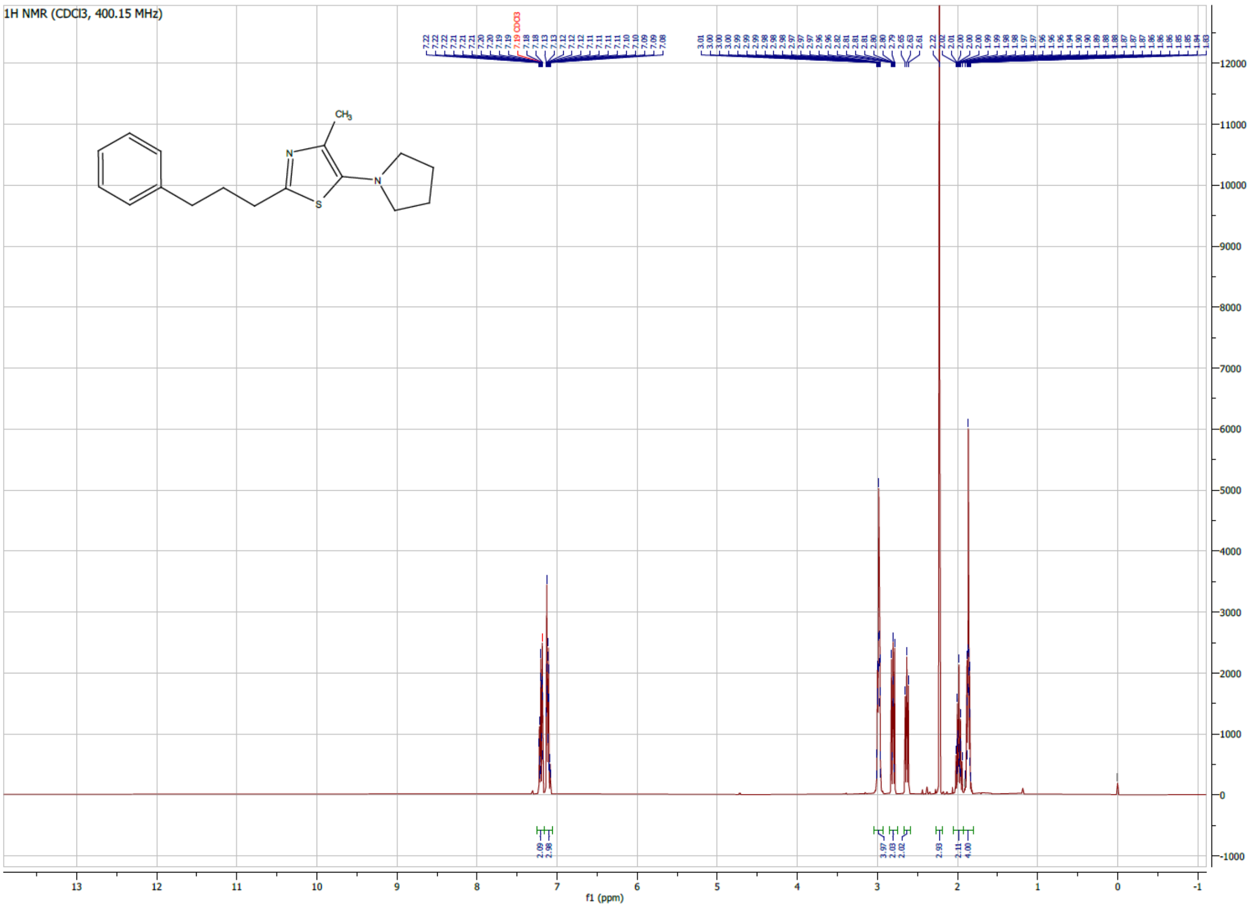

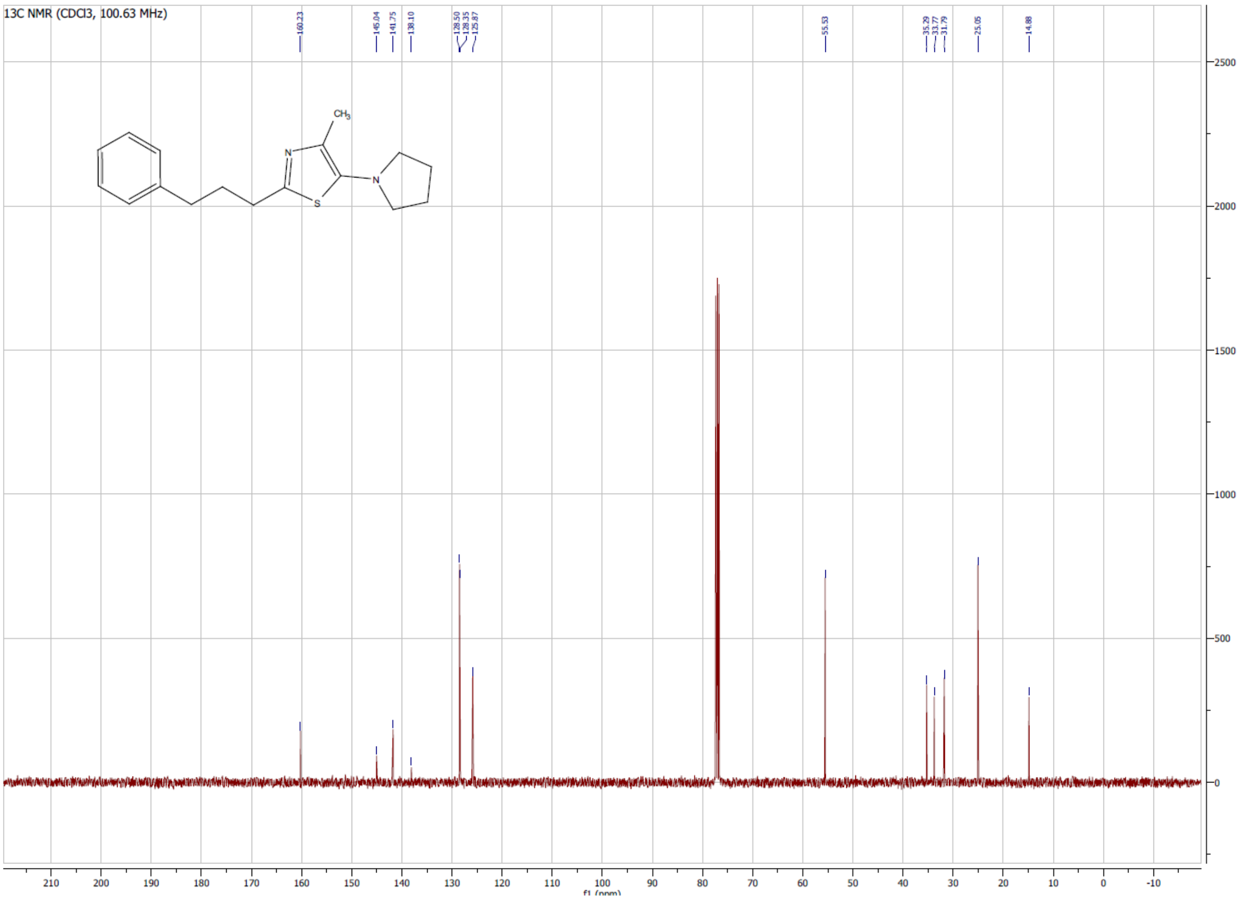

Figure S22. ^{1}H and ^{13}C NMR spectra of compound **7**.

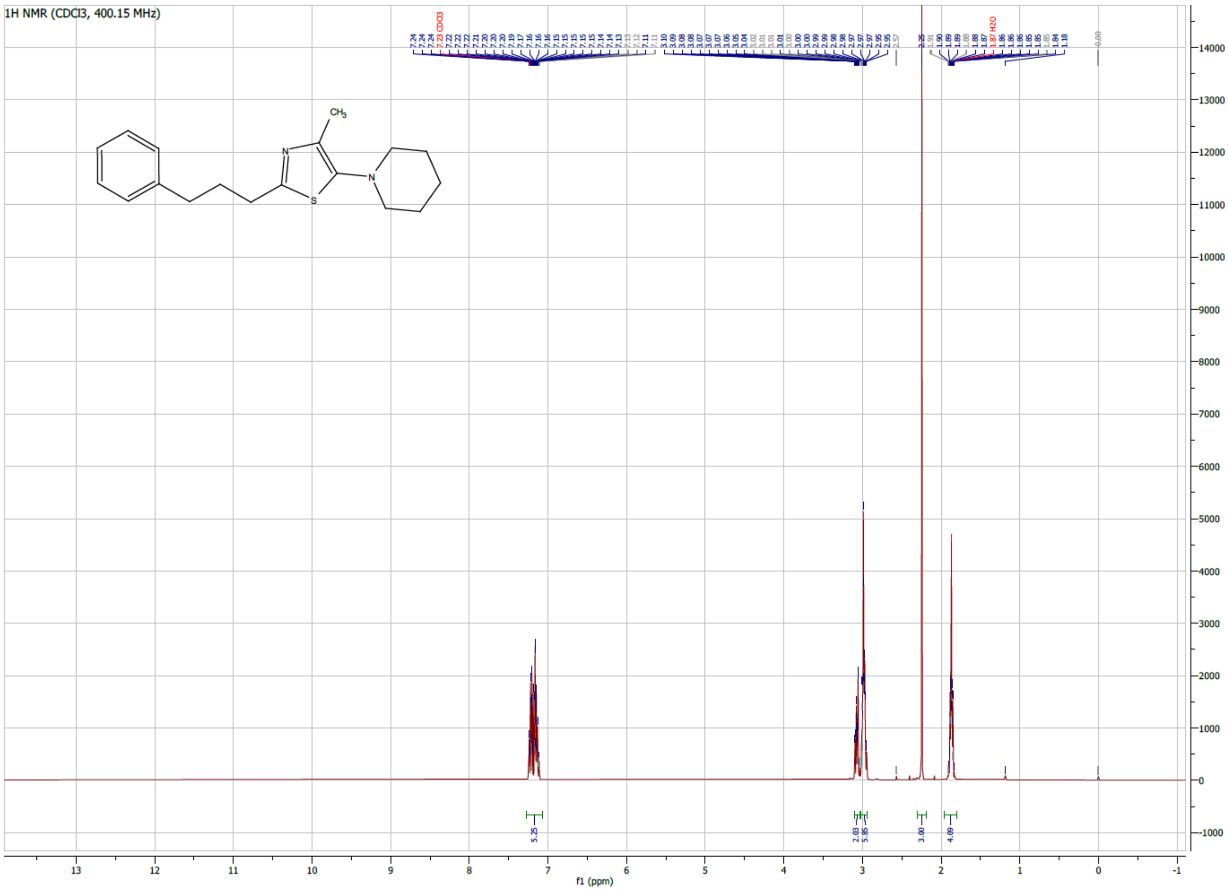

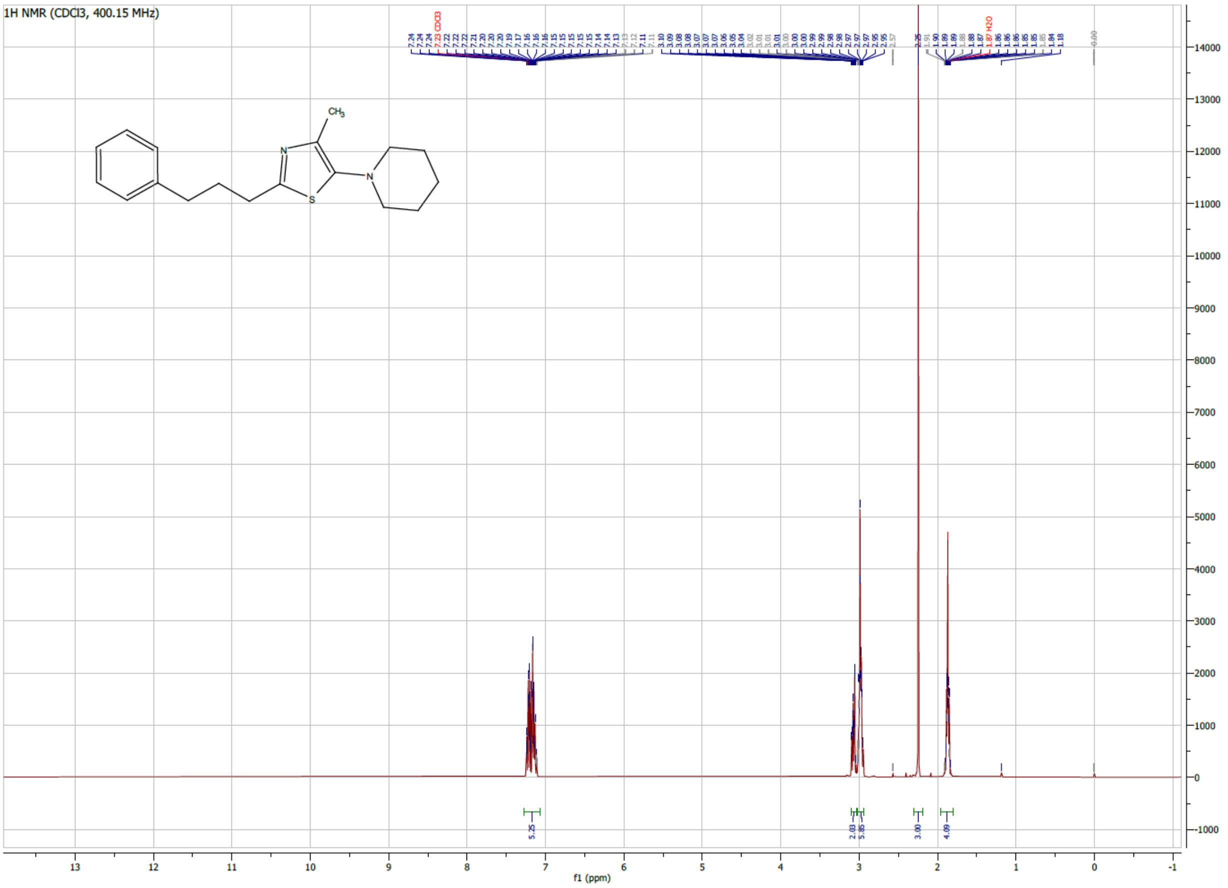

Figure S23. ^{1}H and ^{13}C NMR spectra of compound **8**.

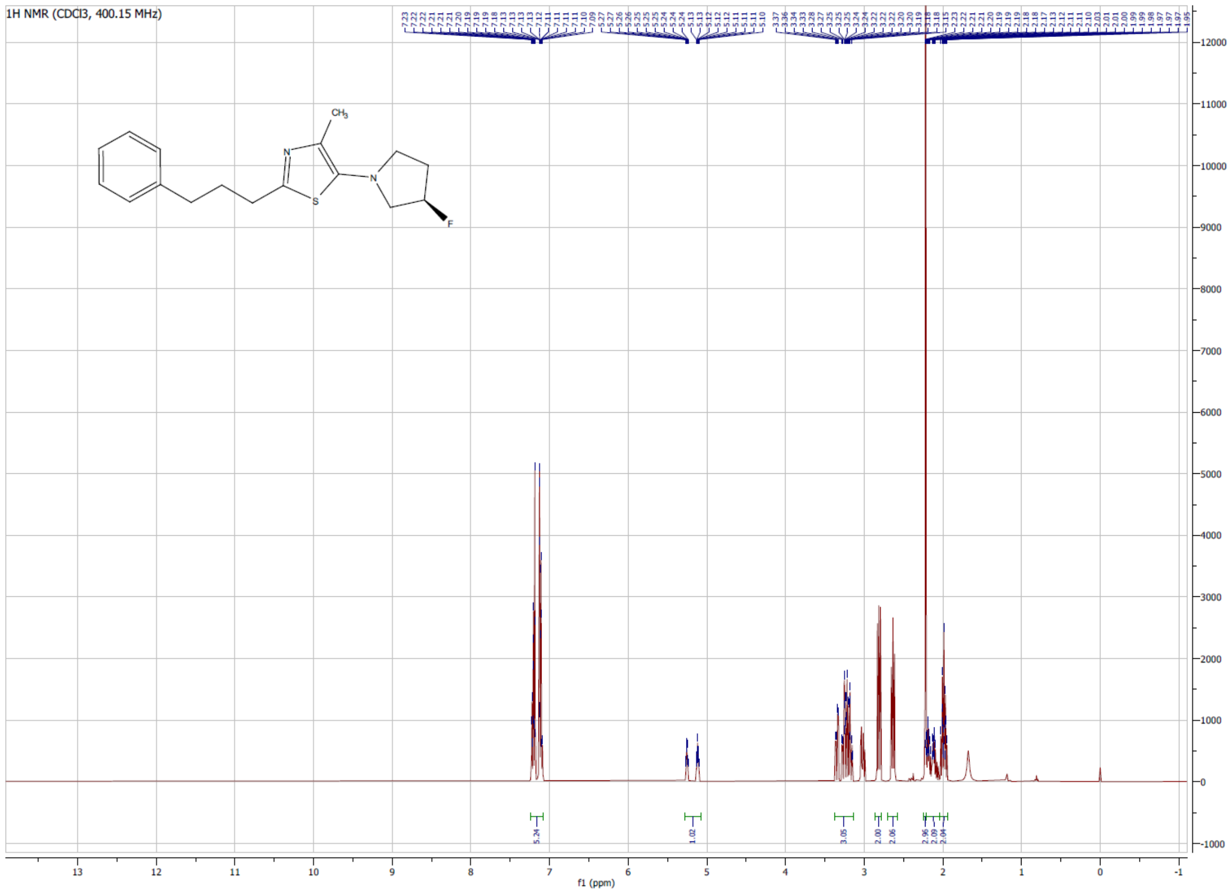

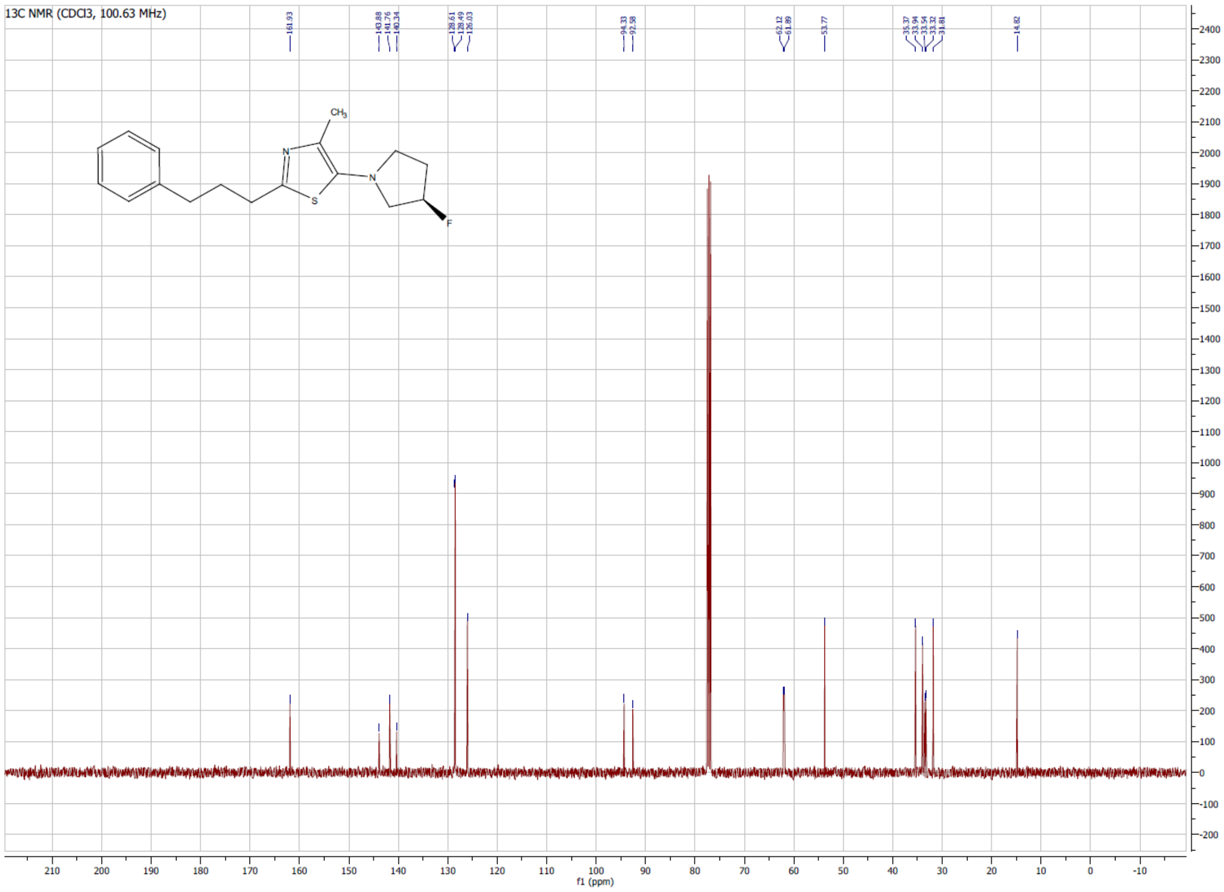

Figure S24. ^{1}H and ^{13}C NMR spectra of compound **9**.

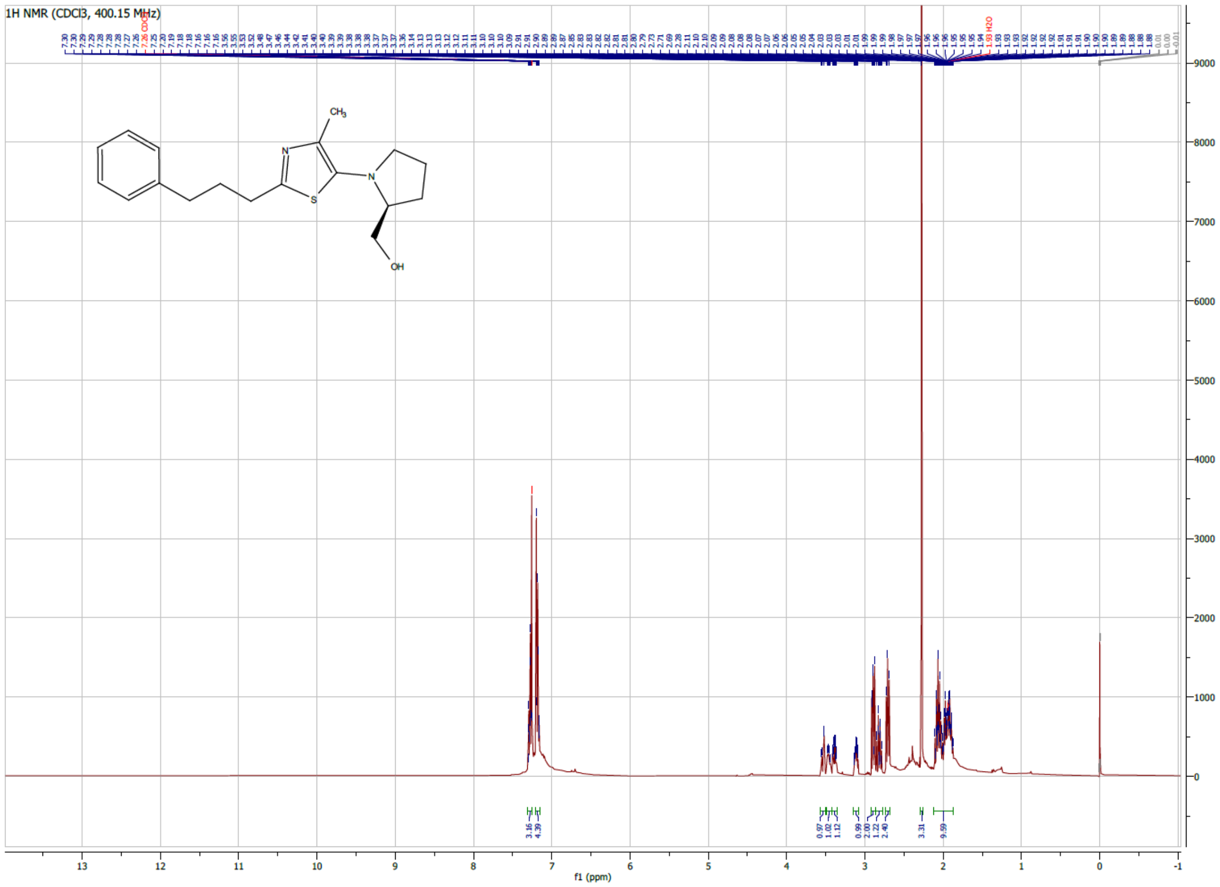

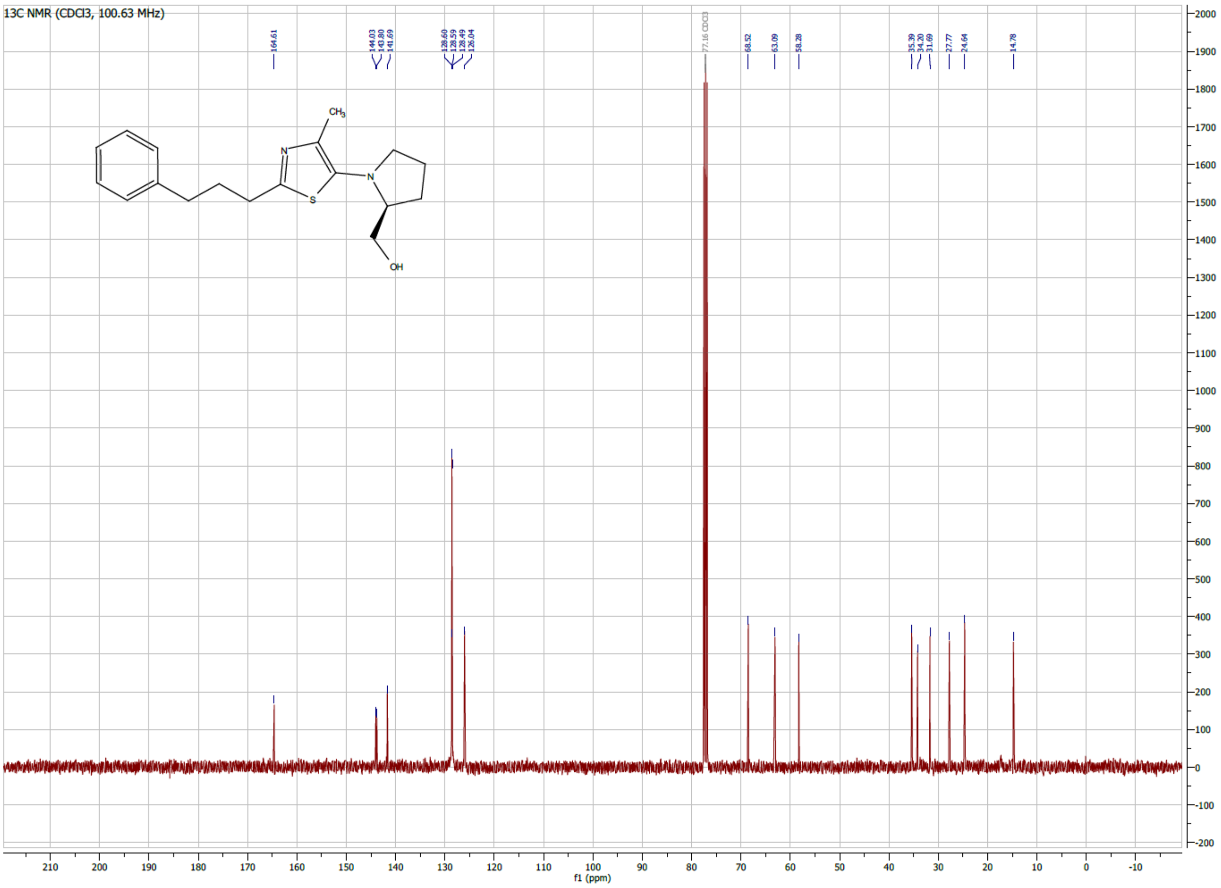

Figure S25. ^{1}H and ^{13}C NMR spectra of compound **10**.

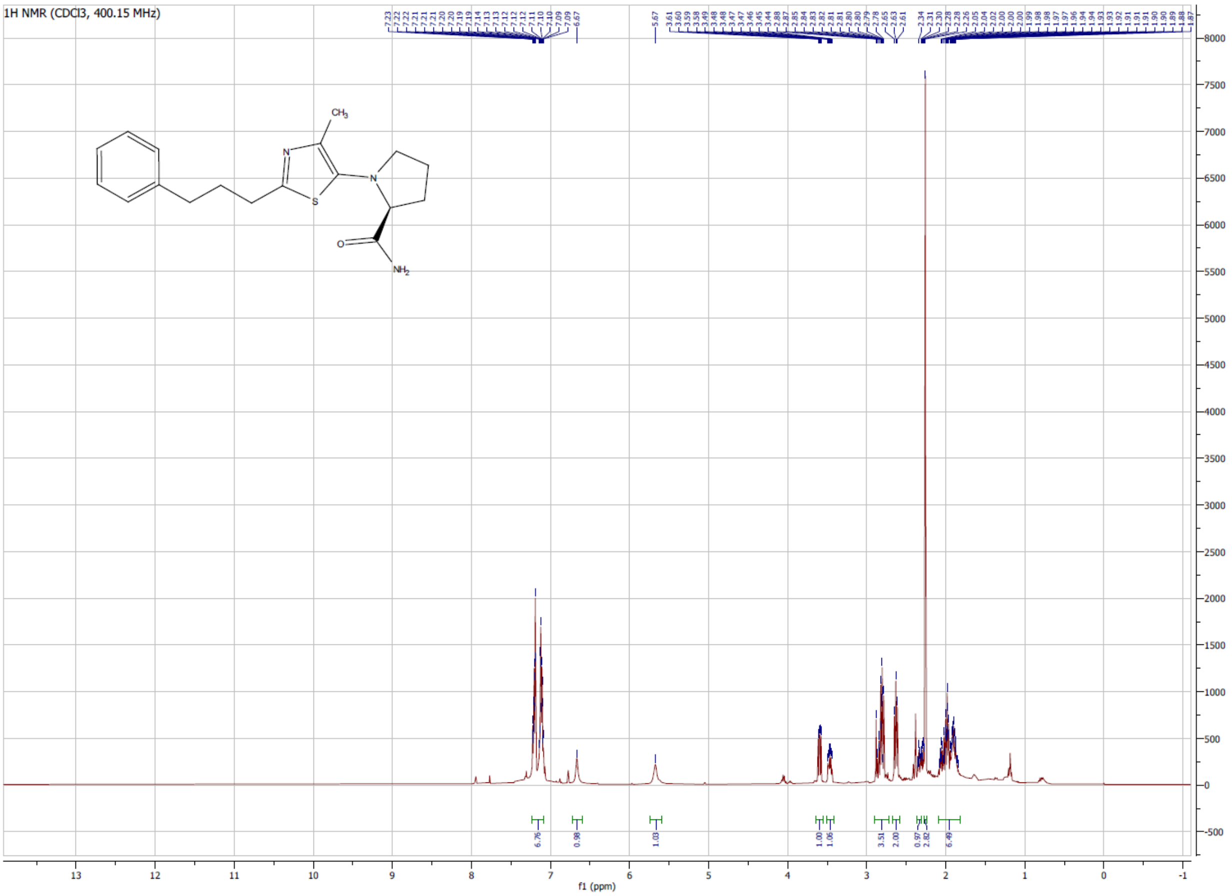

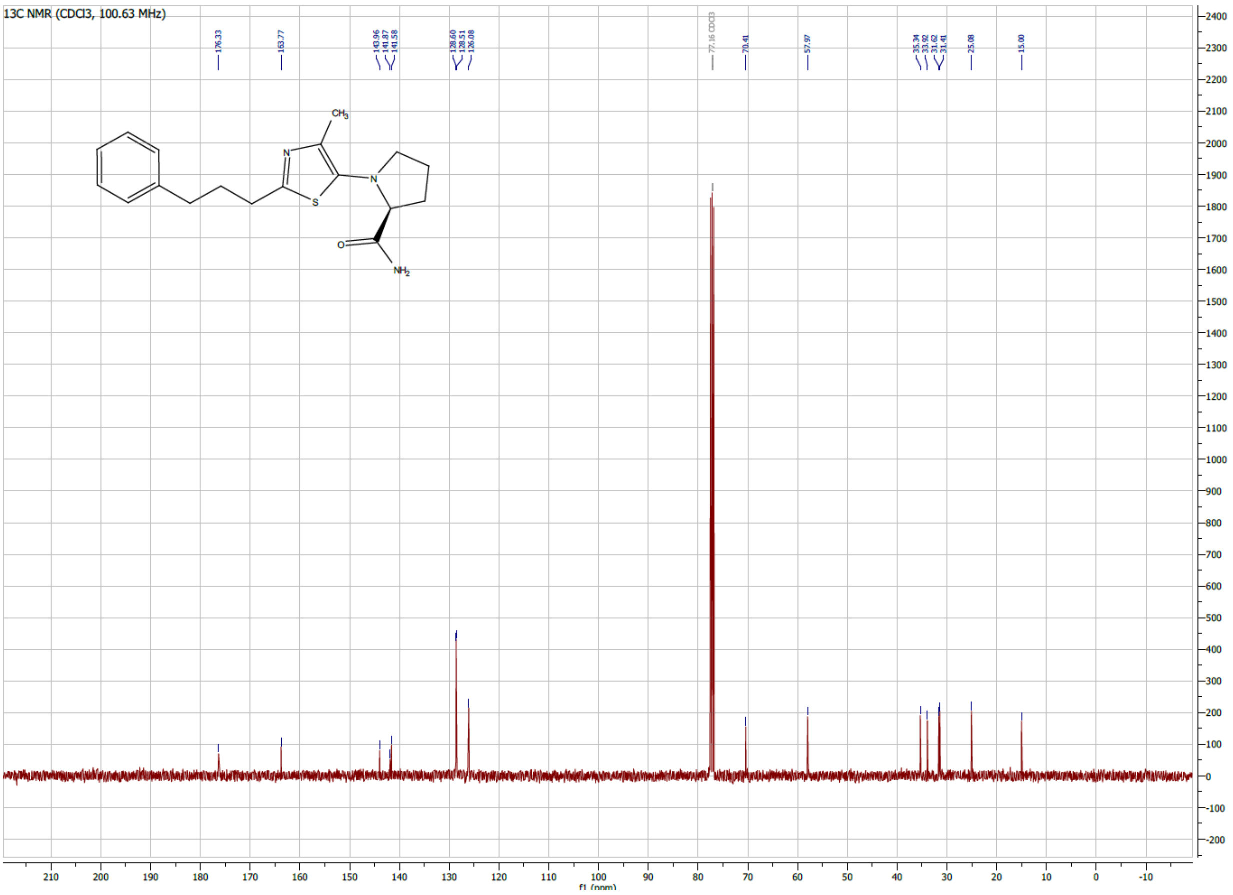

Figure S26. ^{1}H and ^{13}C NMR spectra of compound **11**.

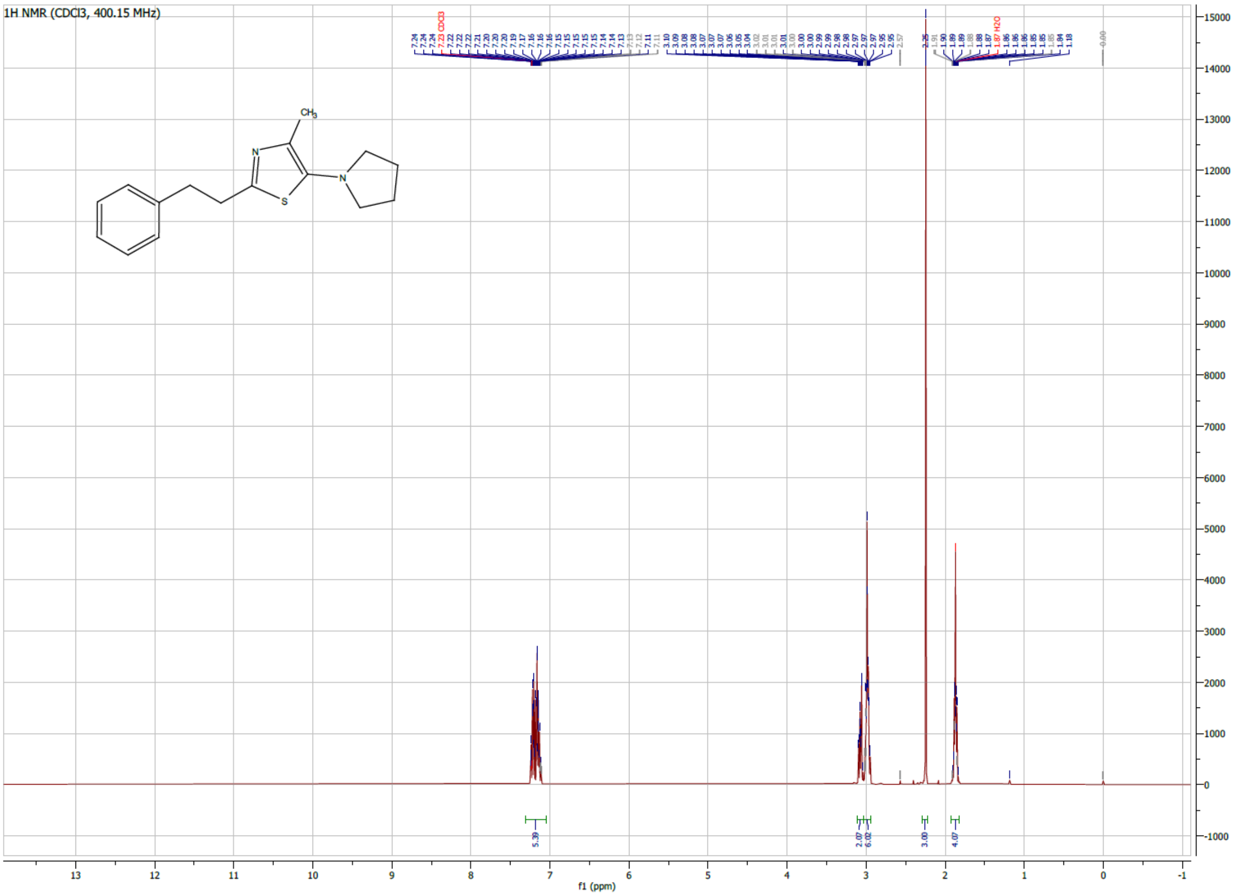

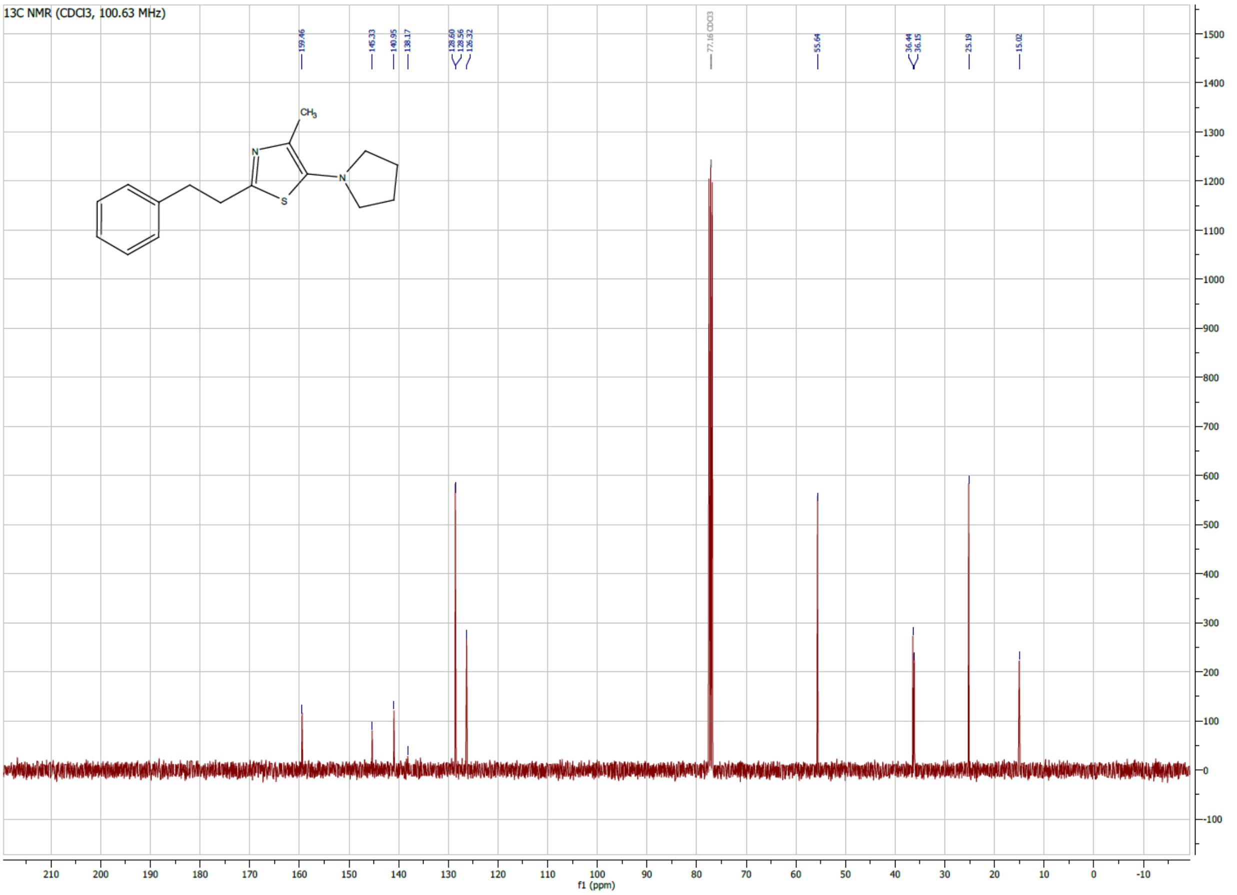

Figure S27. ^{1}H and ^{13}C NMR spectra of compound **12**.

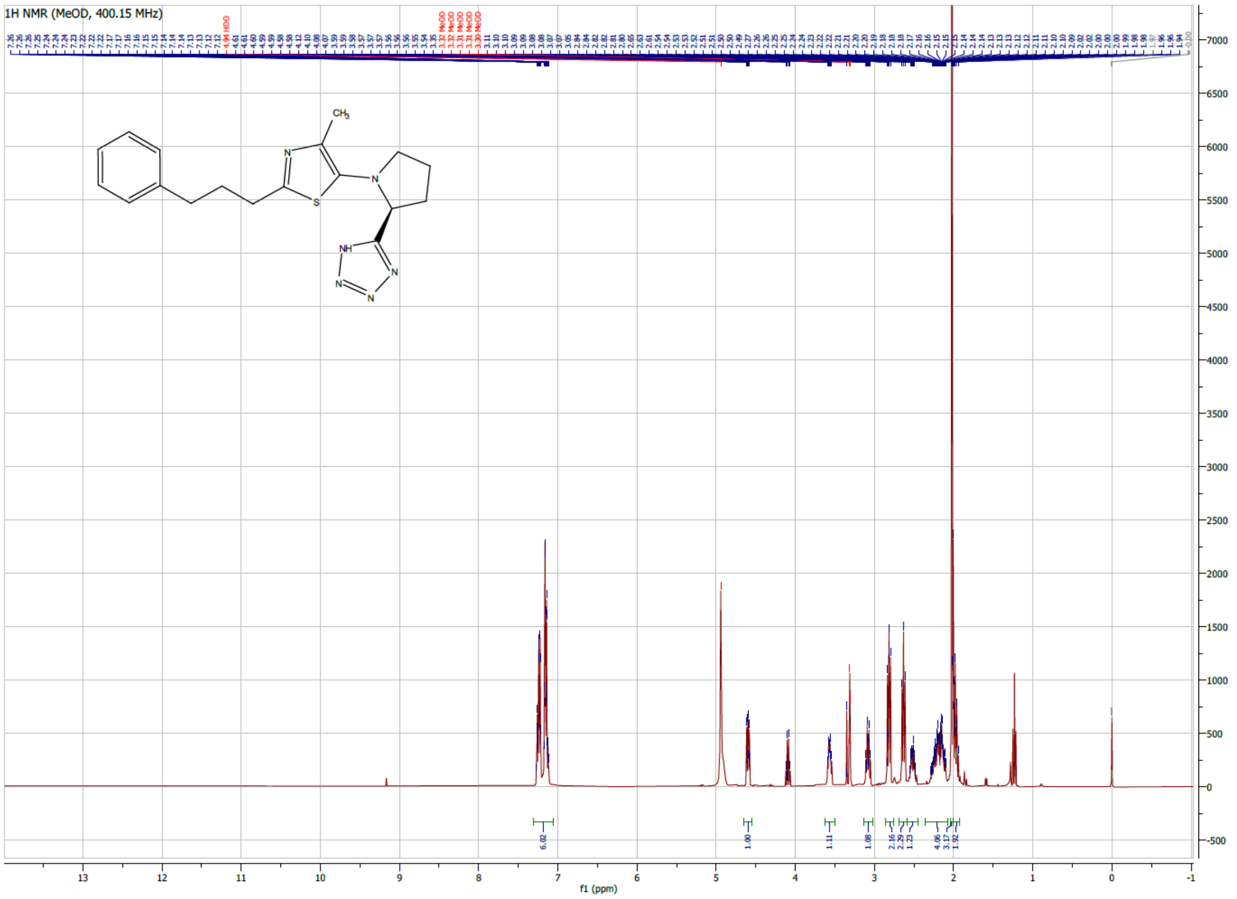

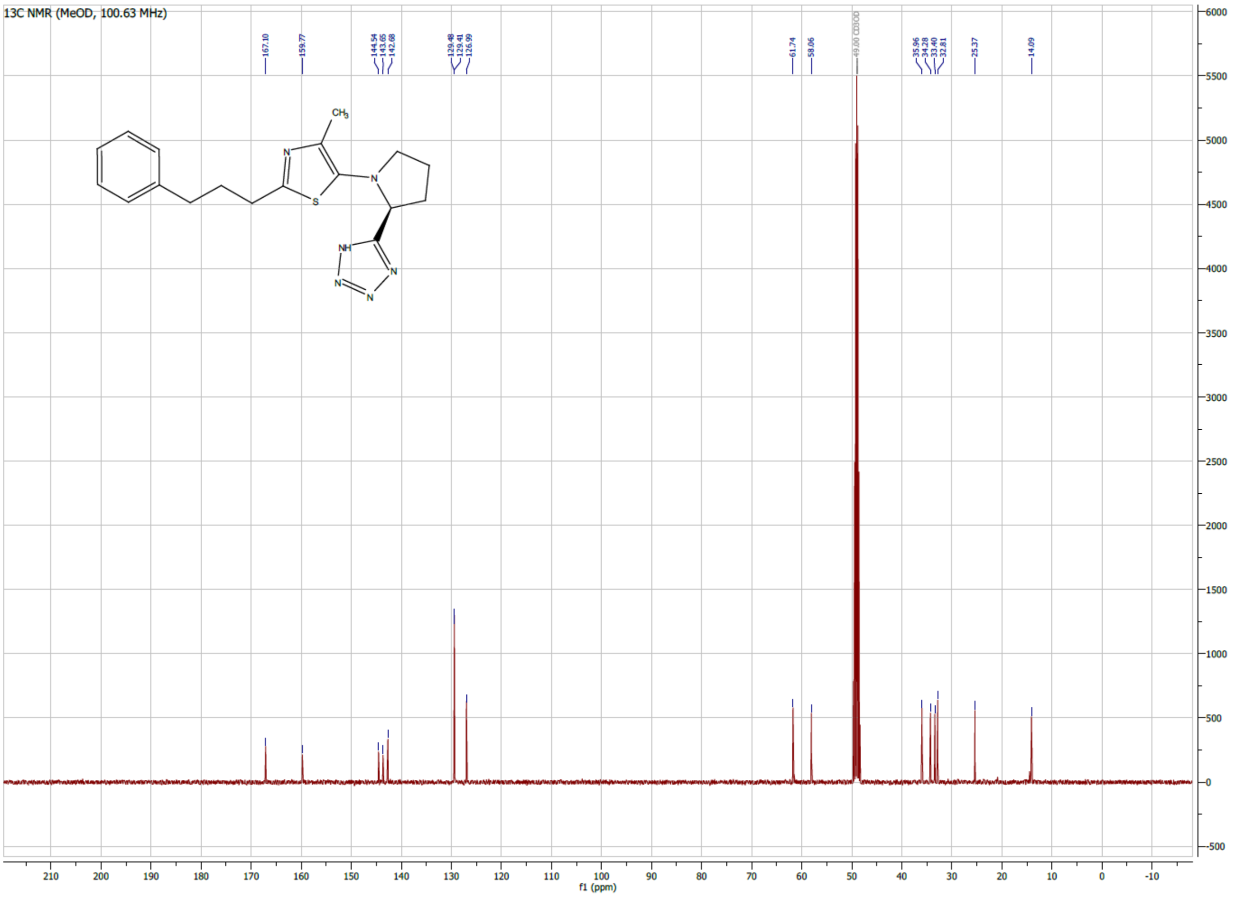

Figure S28. ^{1}H and ^{13}C NMR spectra of compound **13**.

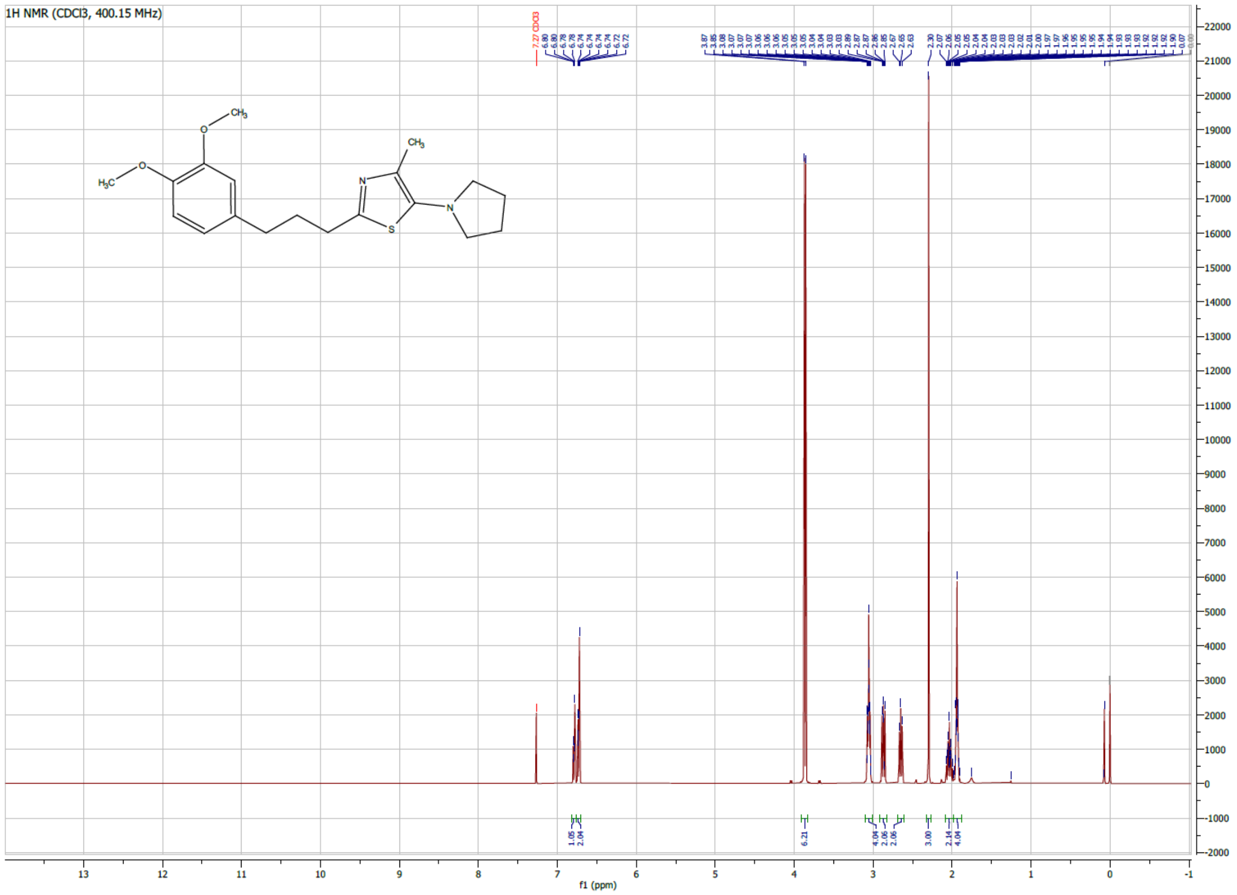

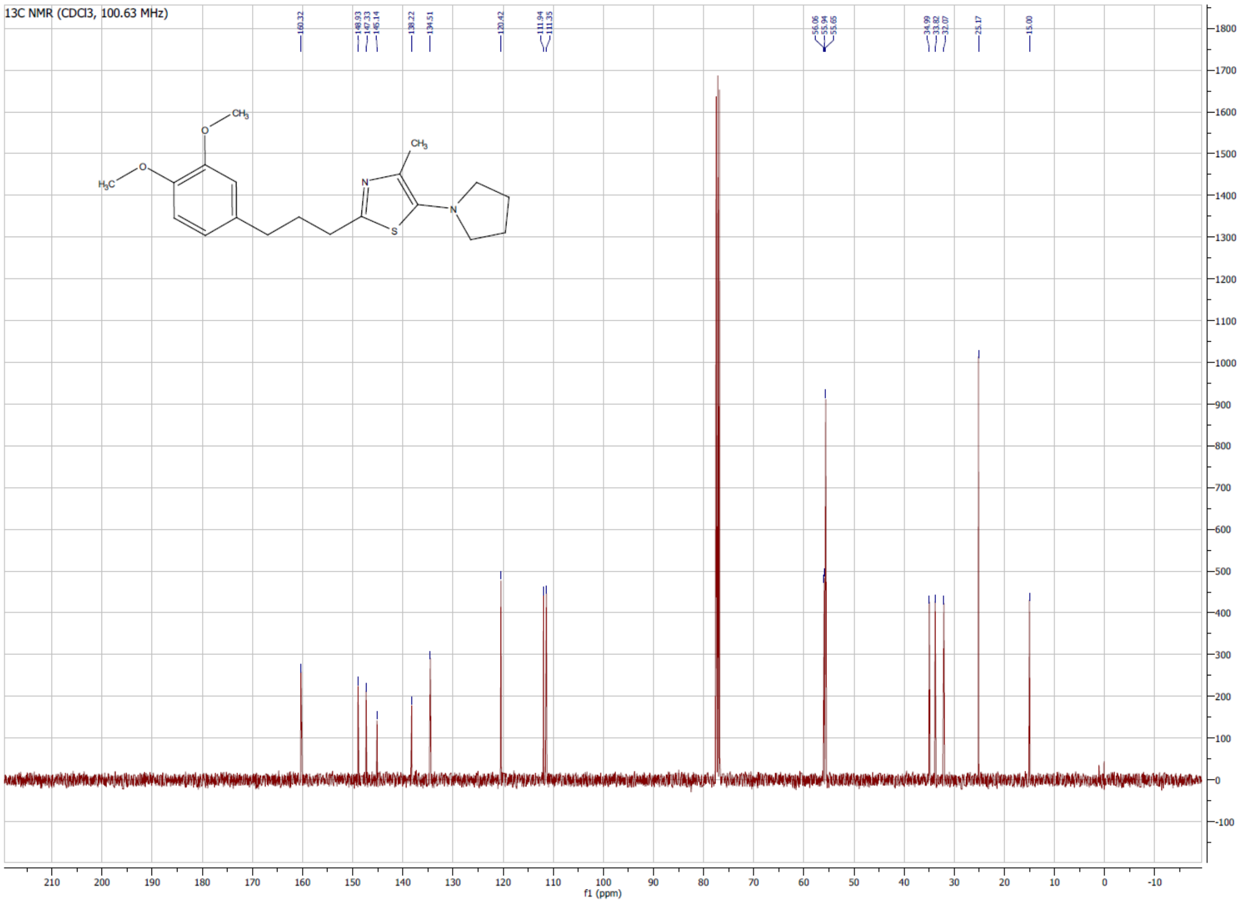

Figure S29. ^{1}H and ^{13}C NMR spectra of compound **16a**.

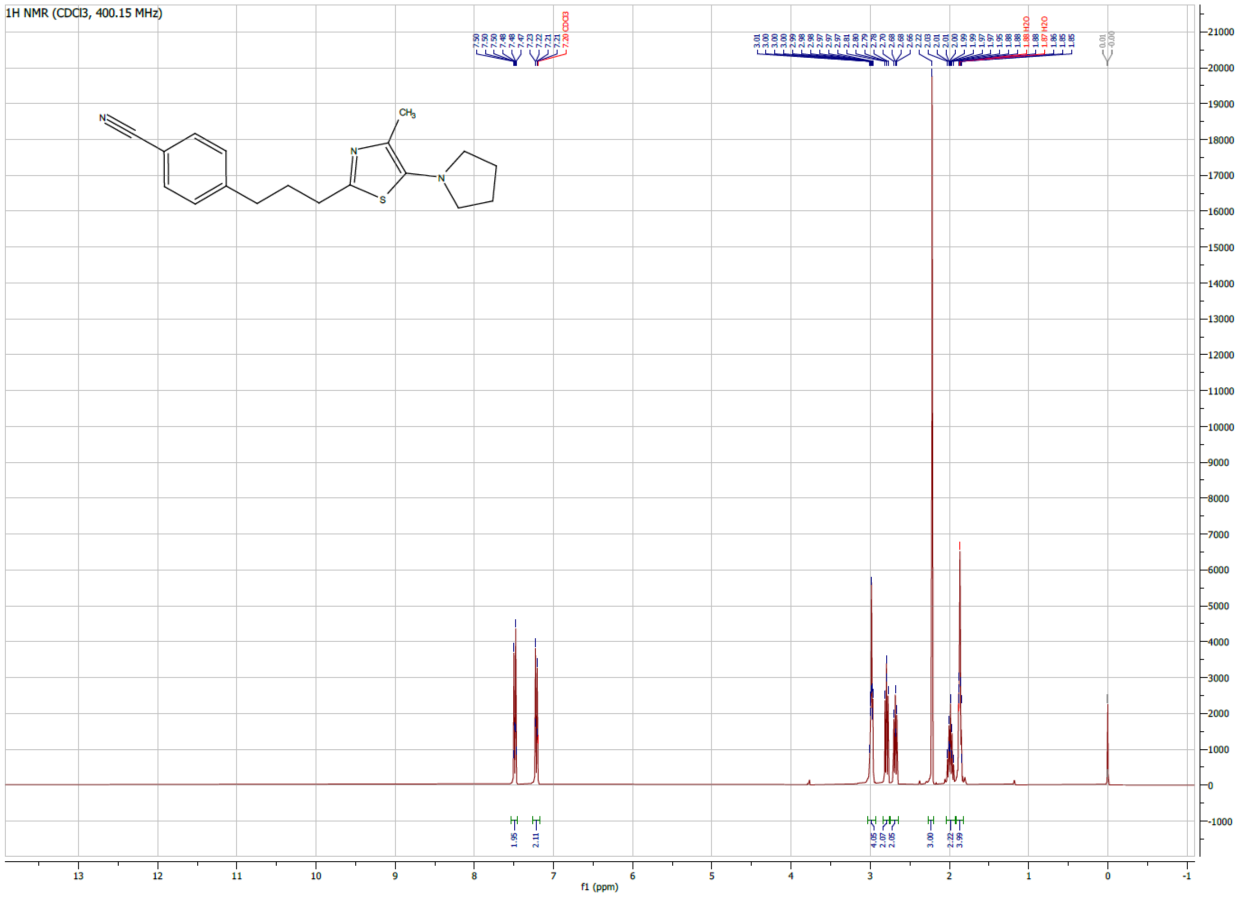

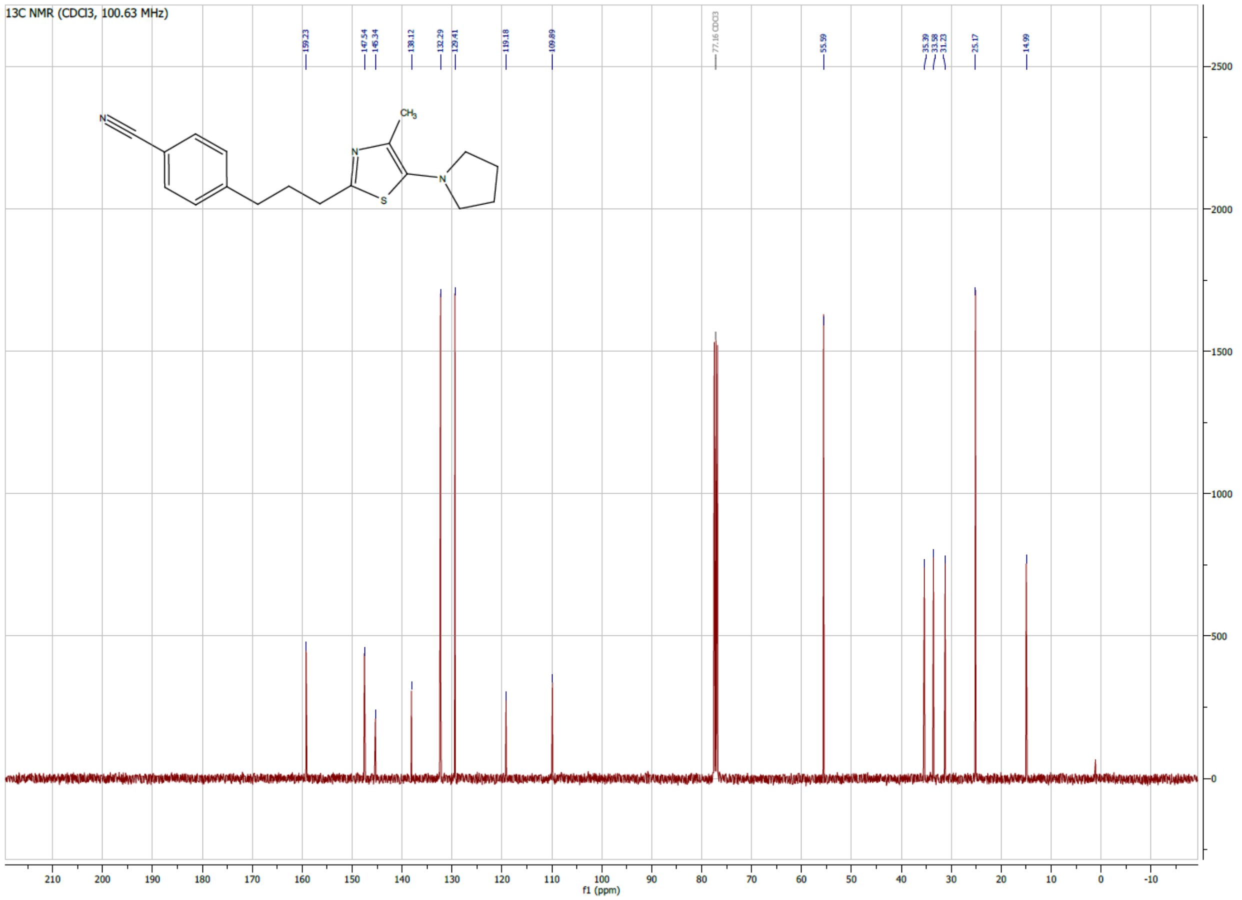

Figure S30. ^{1}H and ^{13}C NMR spectra of compound **16b**.

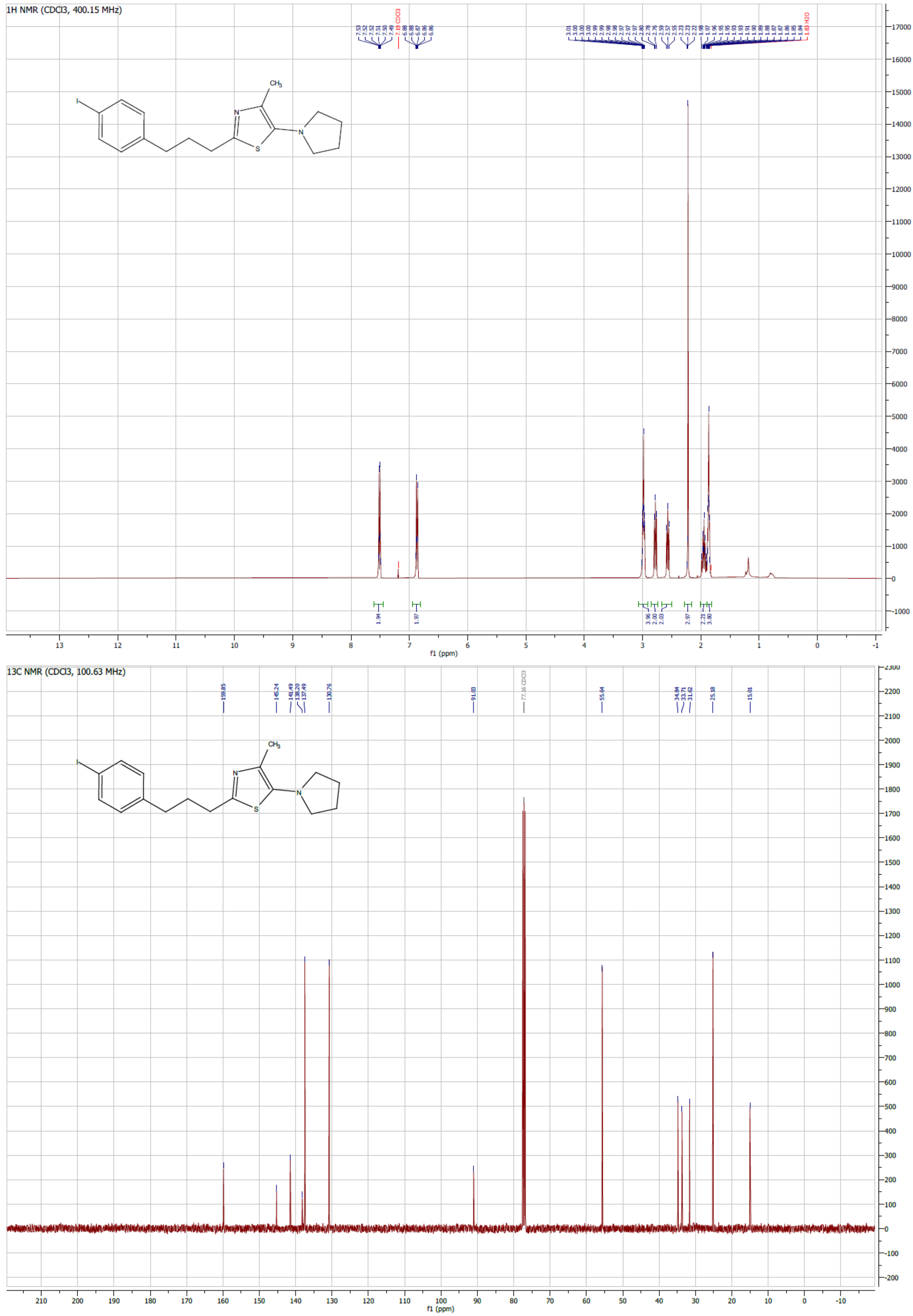

Figure S31. ^{1}H and ^{13}C NMR spectra of compound **16c**.

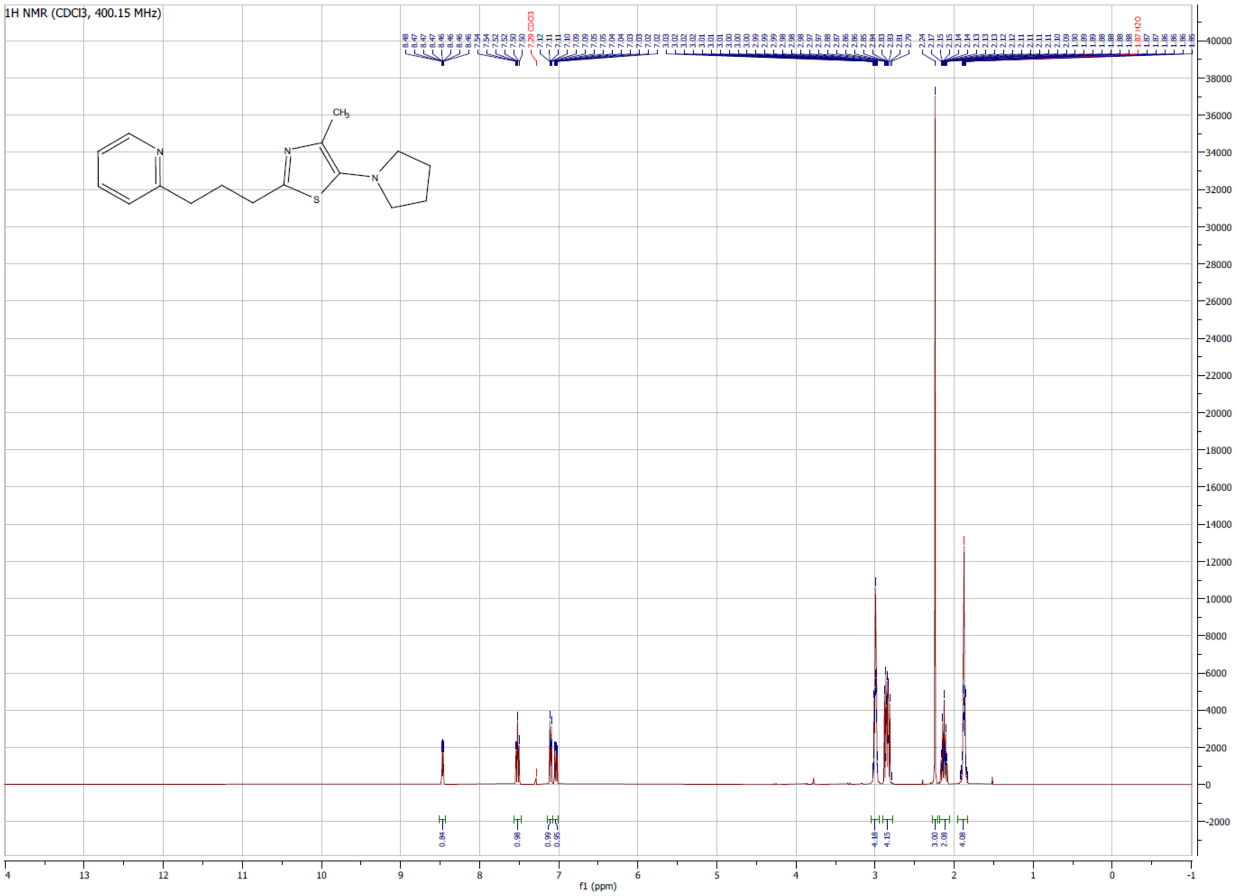

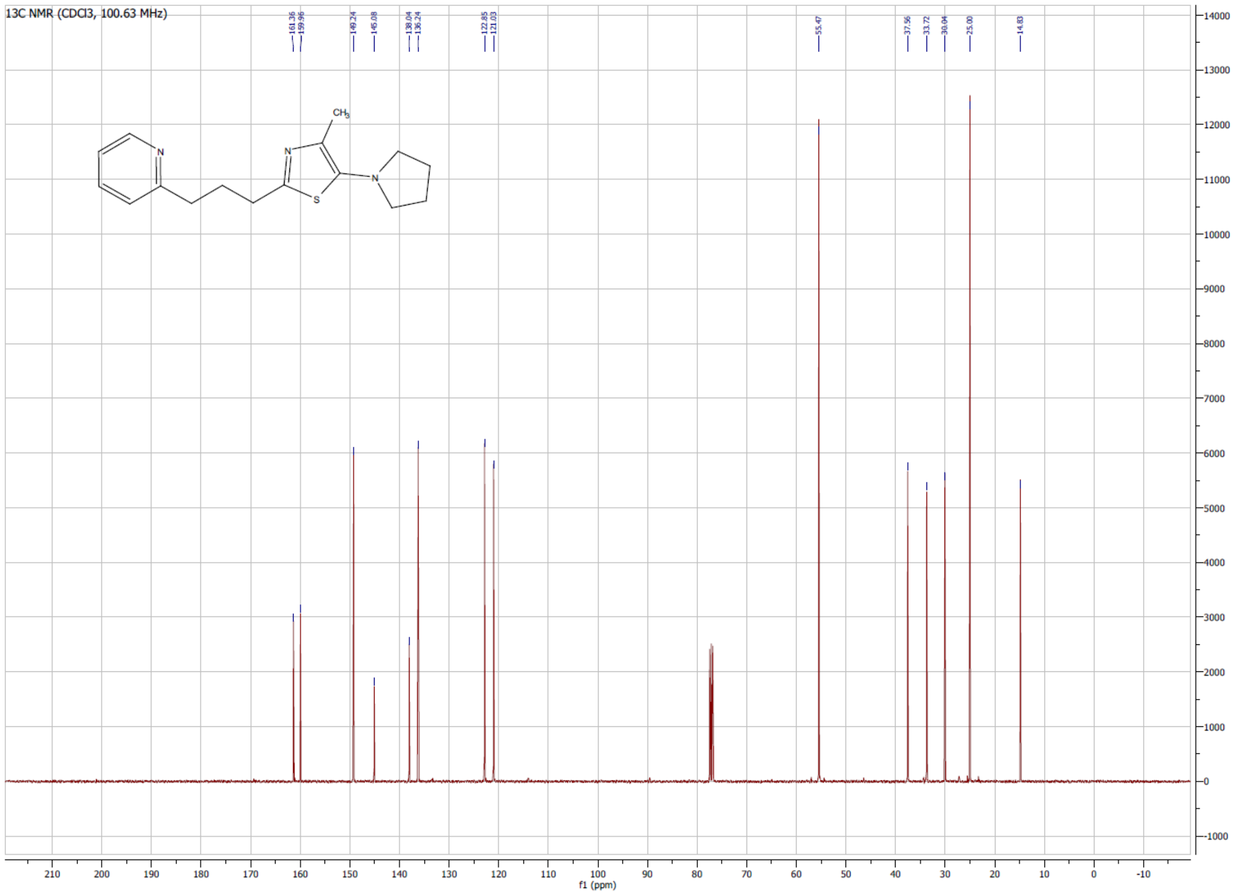

Figure S32. ^{1}H and ^{13}C NMR spectra of compound **16d**.

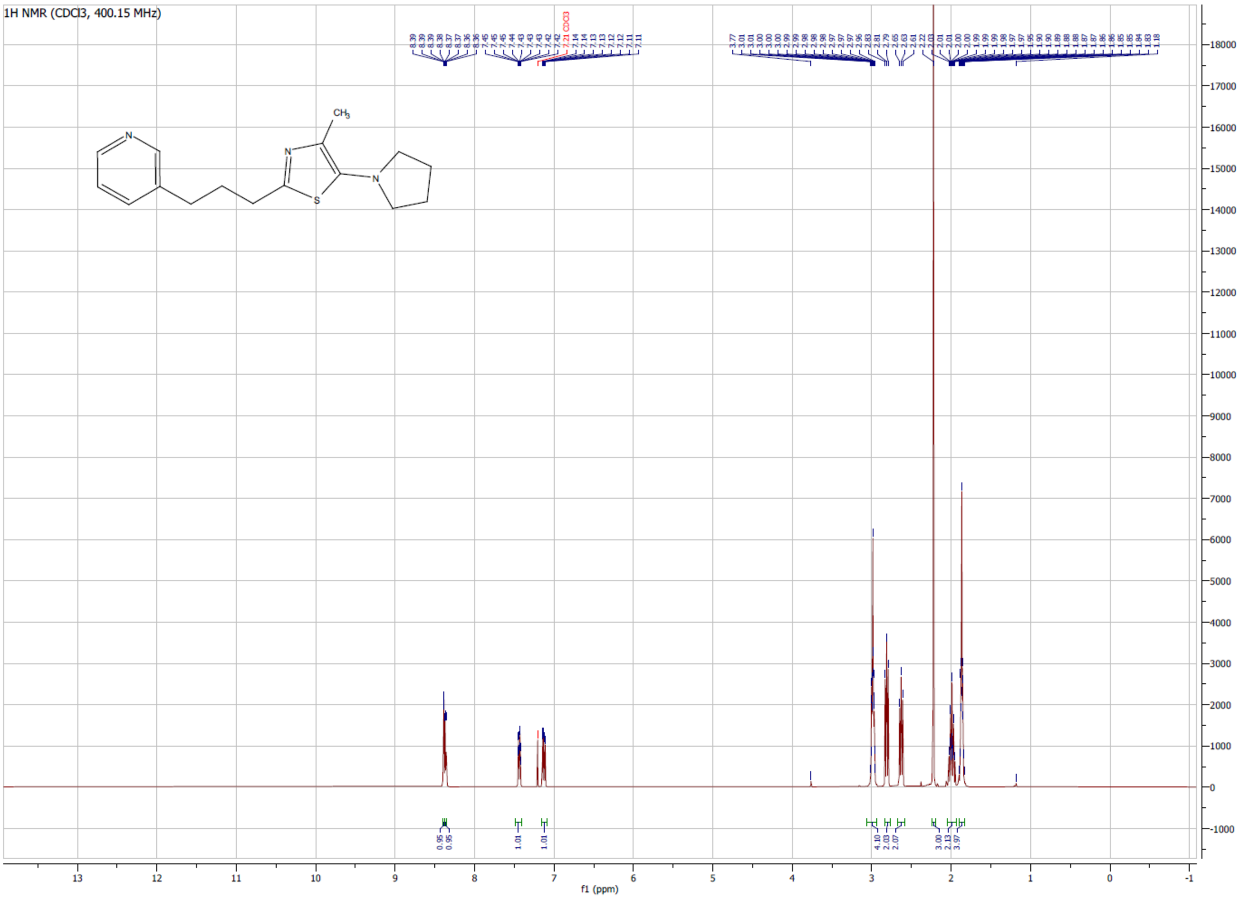

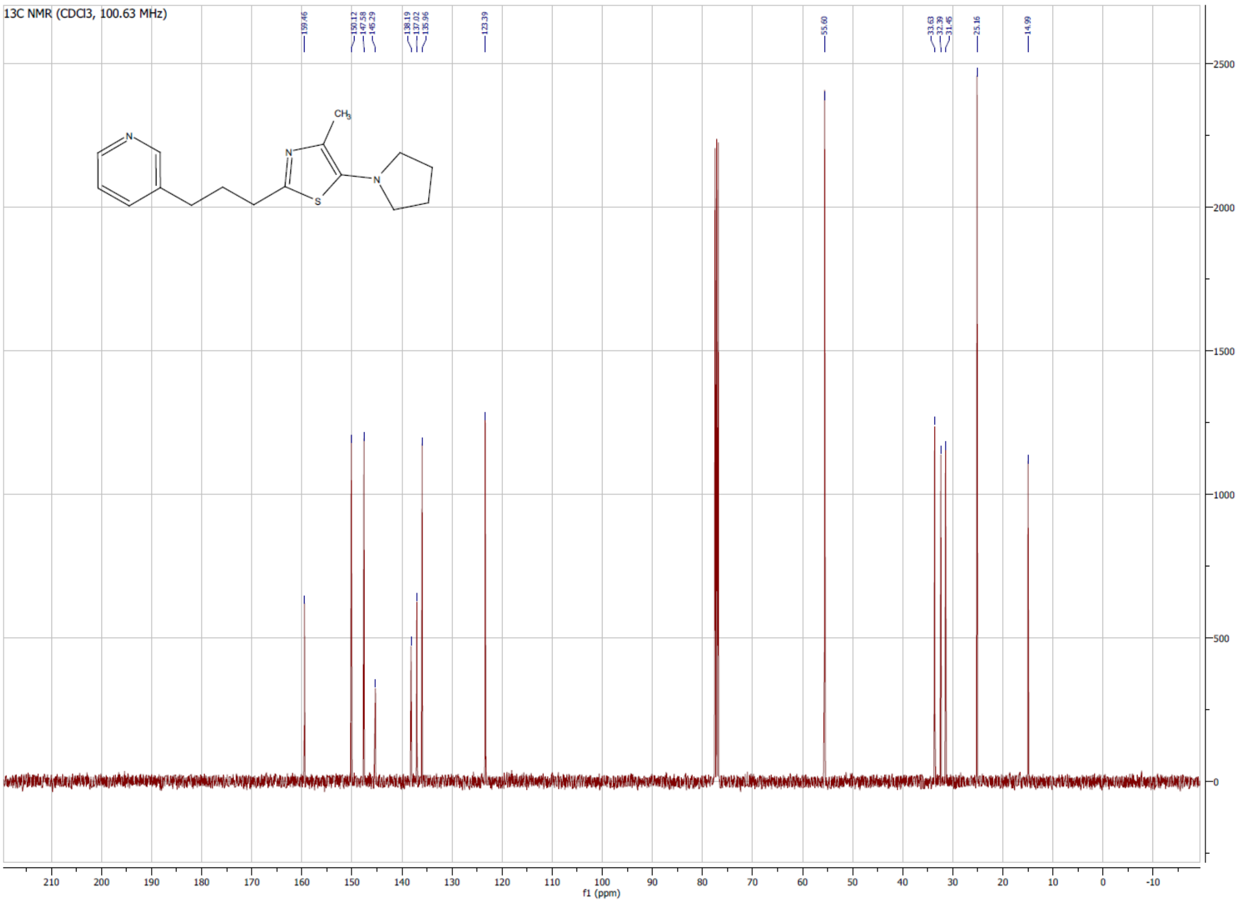

Figure S33. ^{1}H and ^{13}C NMR spectra of compound **16e**.

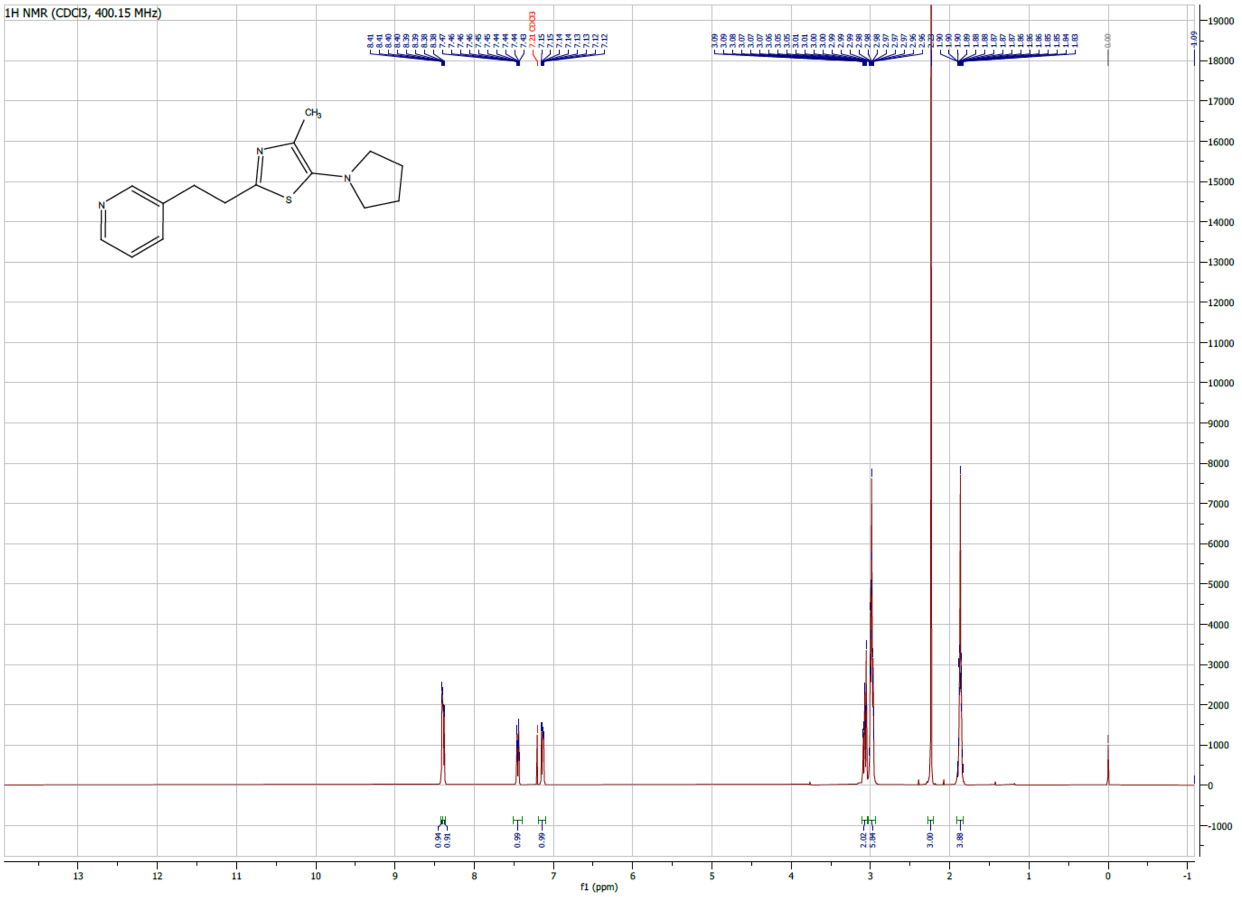

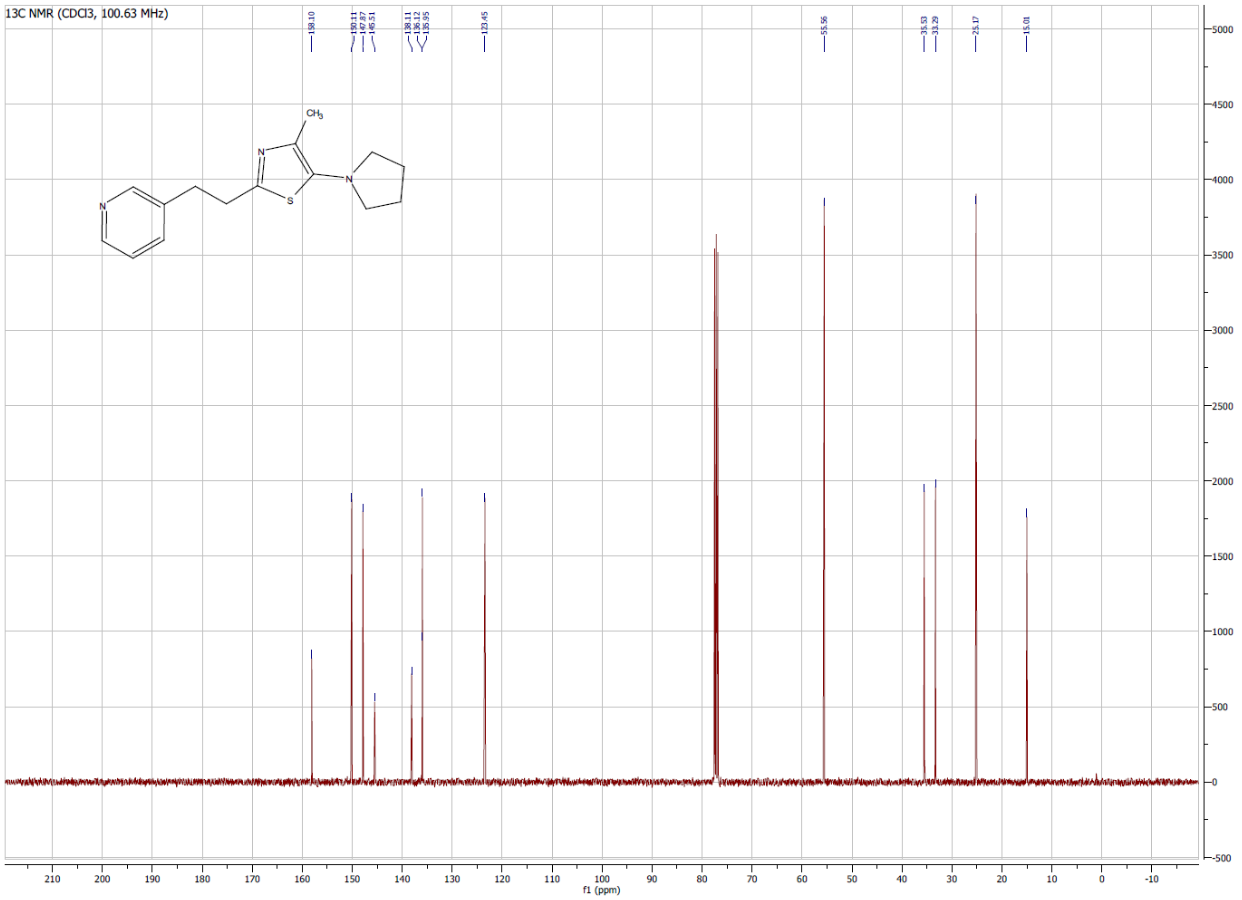

Figure S34. ^{1}H and ^{13}C NMR spectra of compound **16f**.

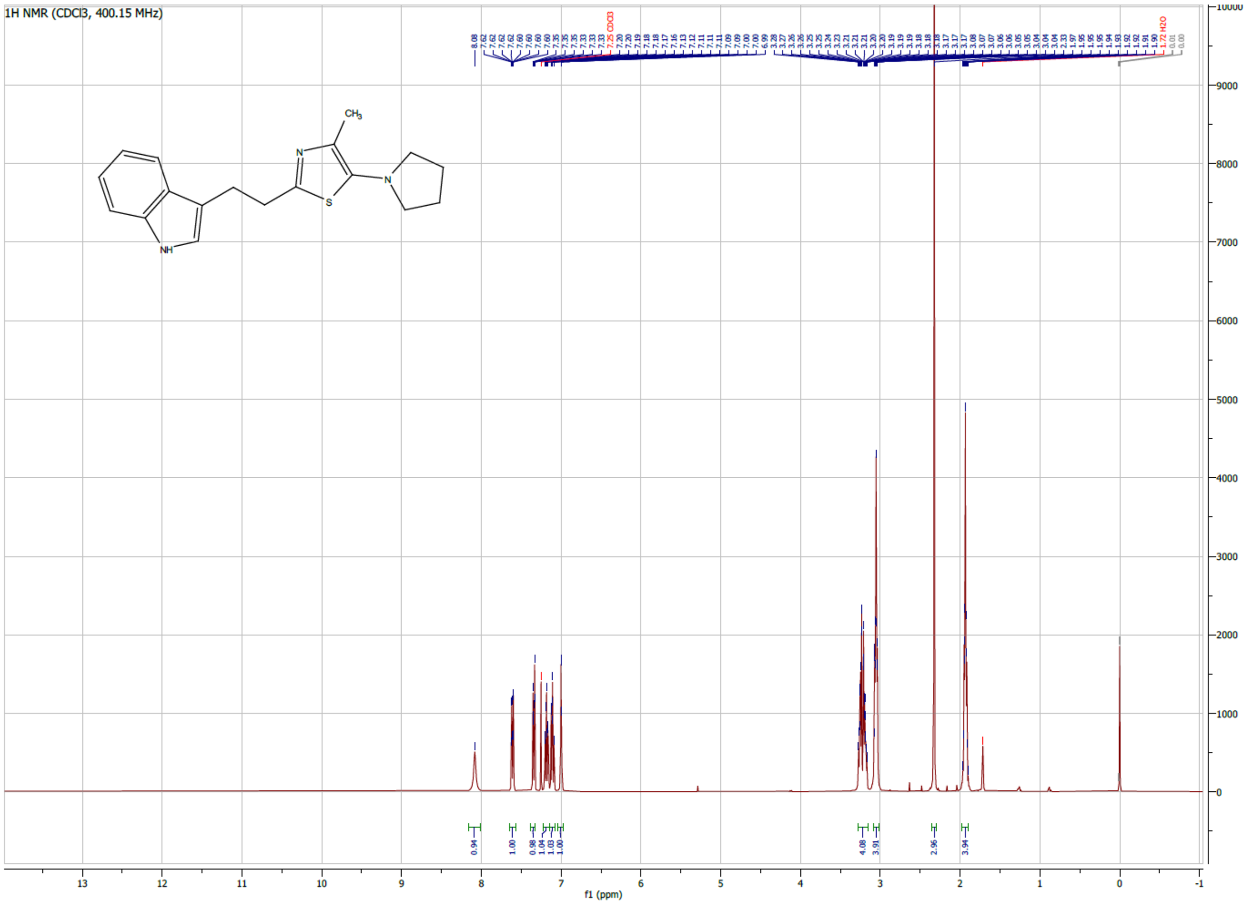

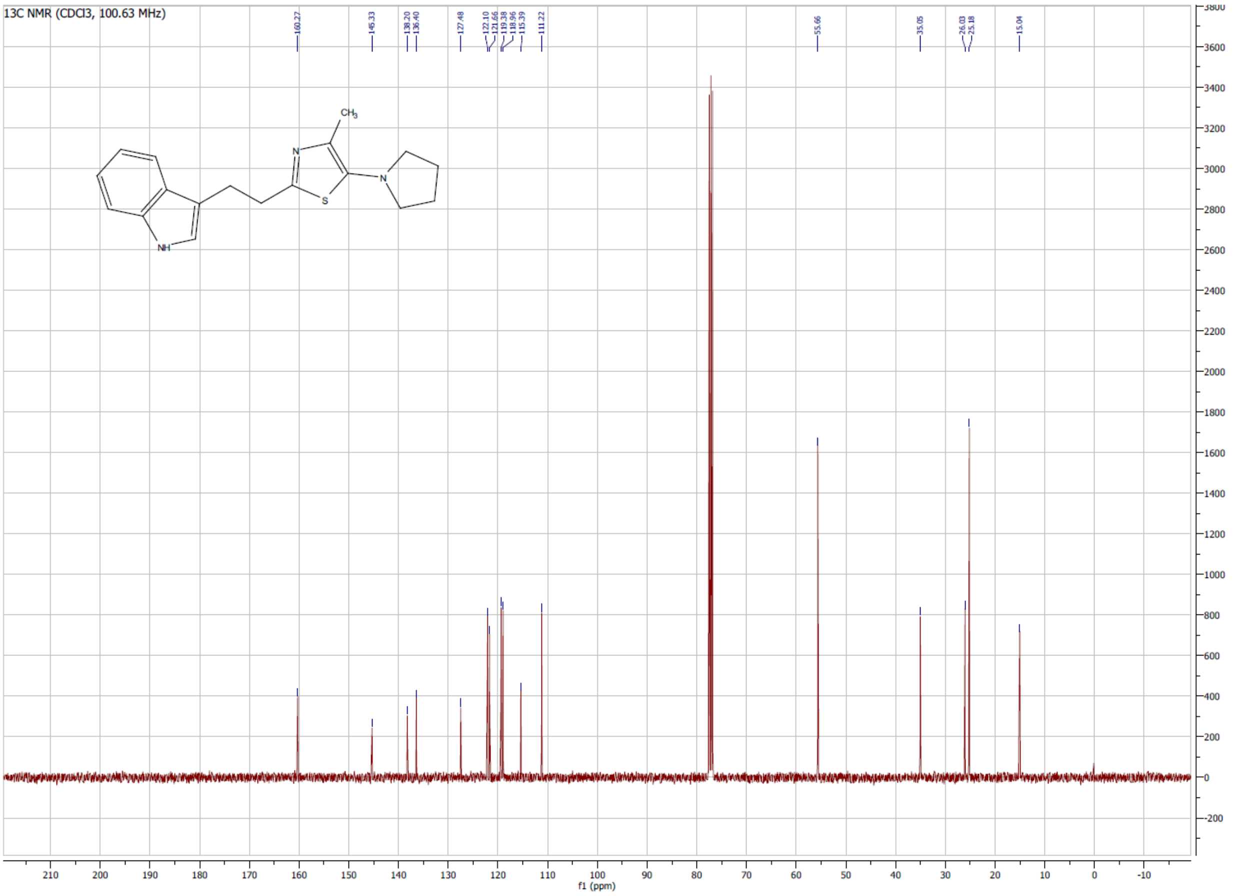

Figure S35. ^{1}H and ^{13}C NMR spectra of compound **16g**.

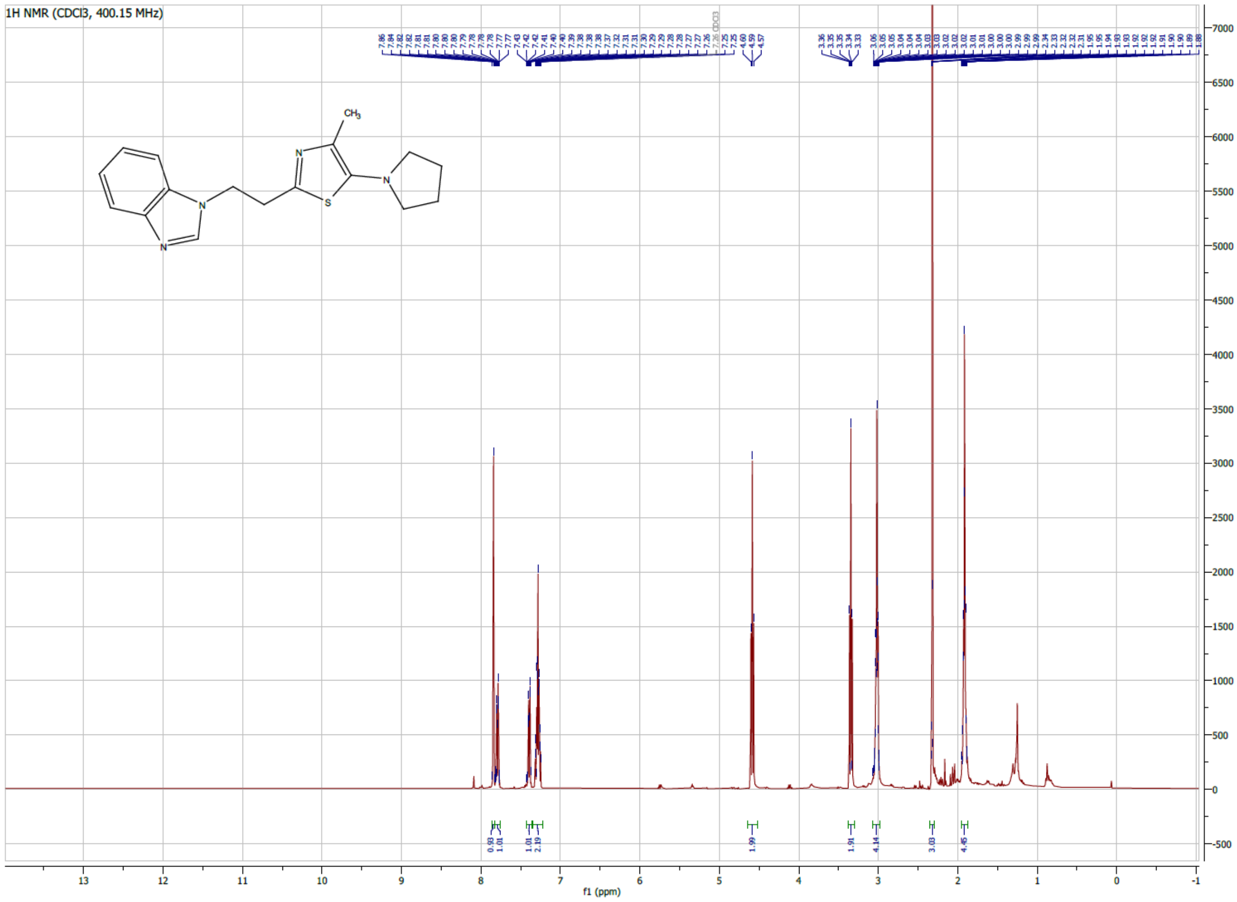

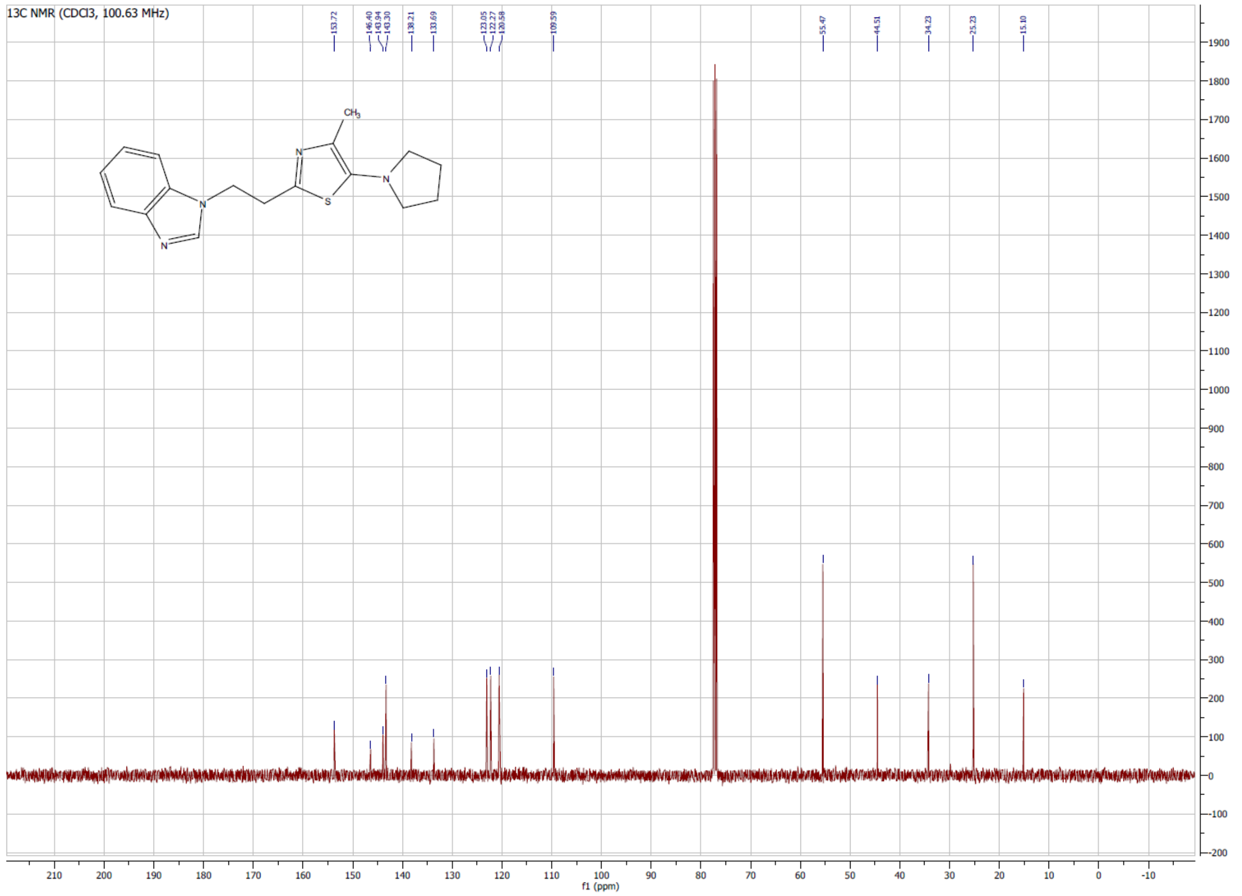

Figure S36. ^{1}H and ^{13}C NMR spectra of compound **16h**.

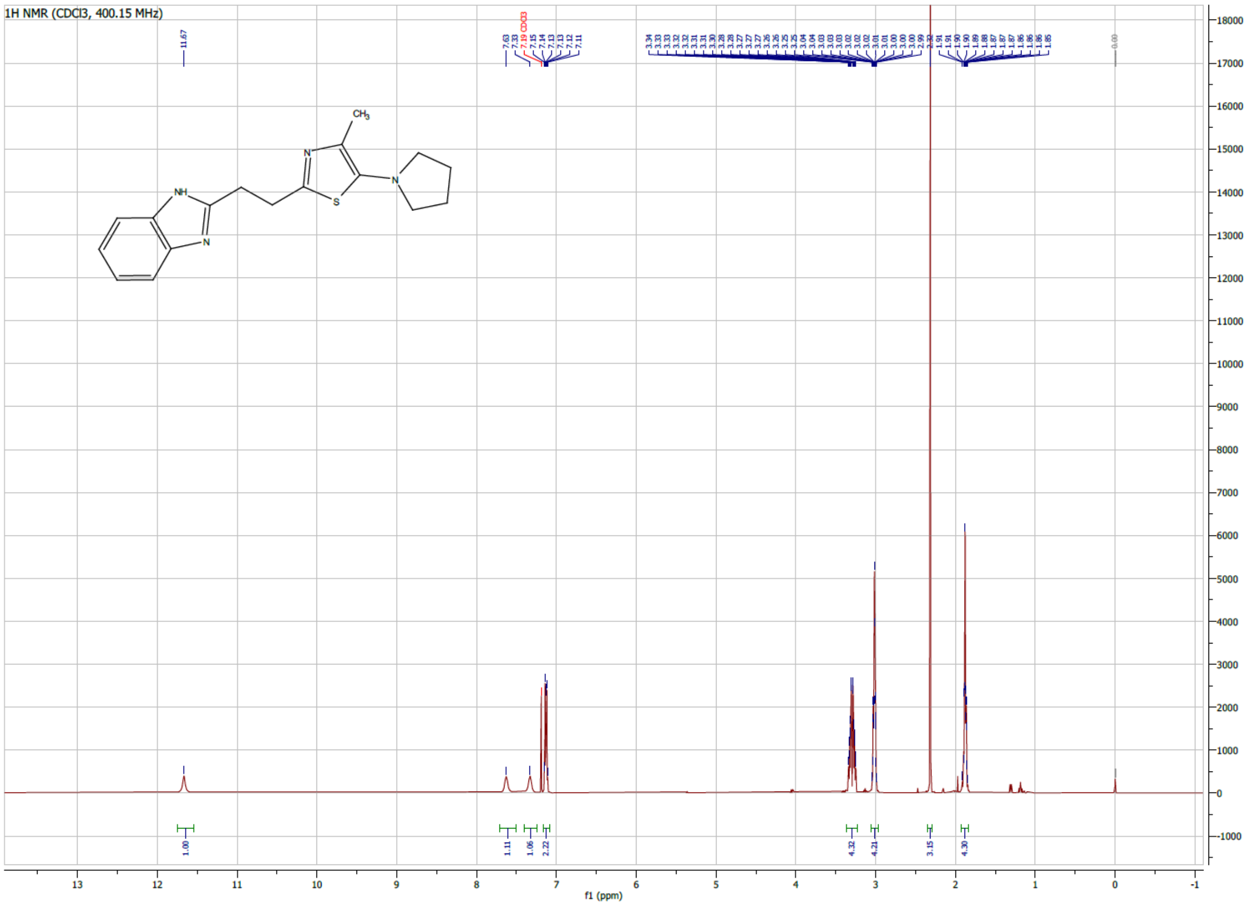

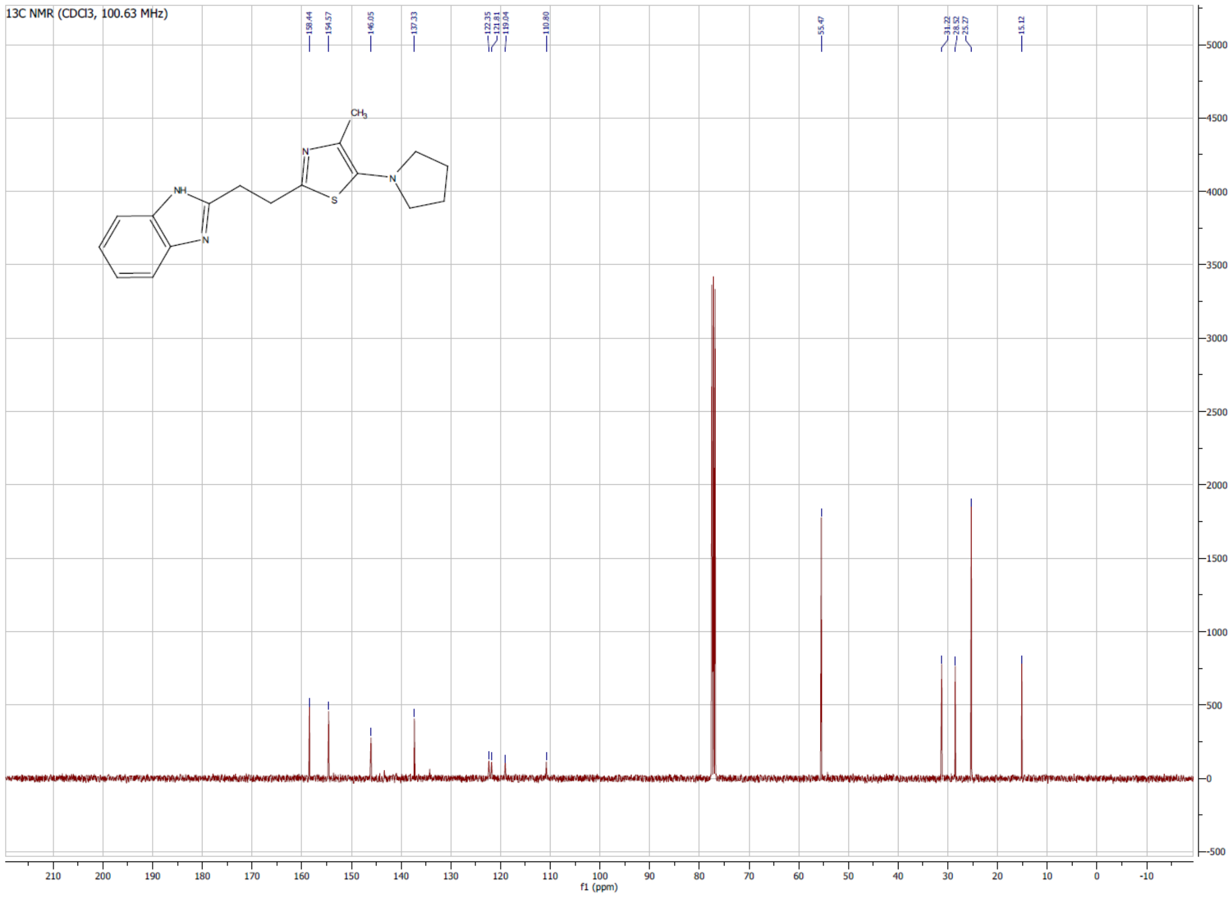

Figure S37. ^{1}H and ^{13}C NMR spectra of compound **16i**.

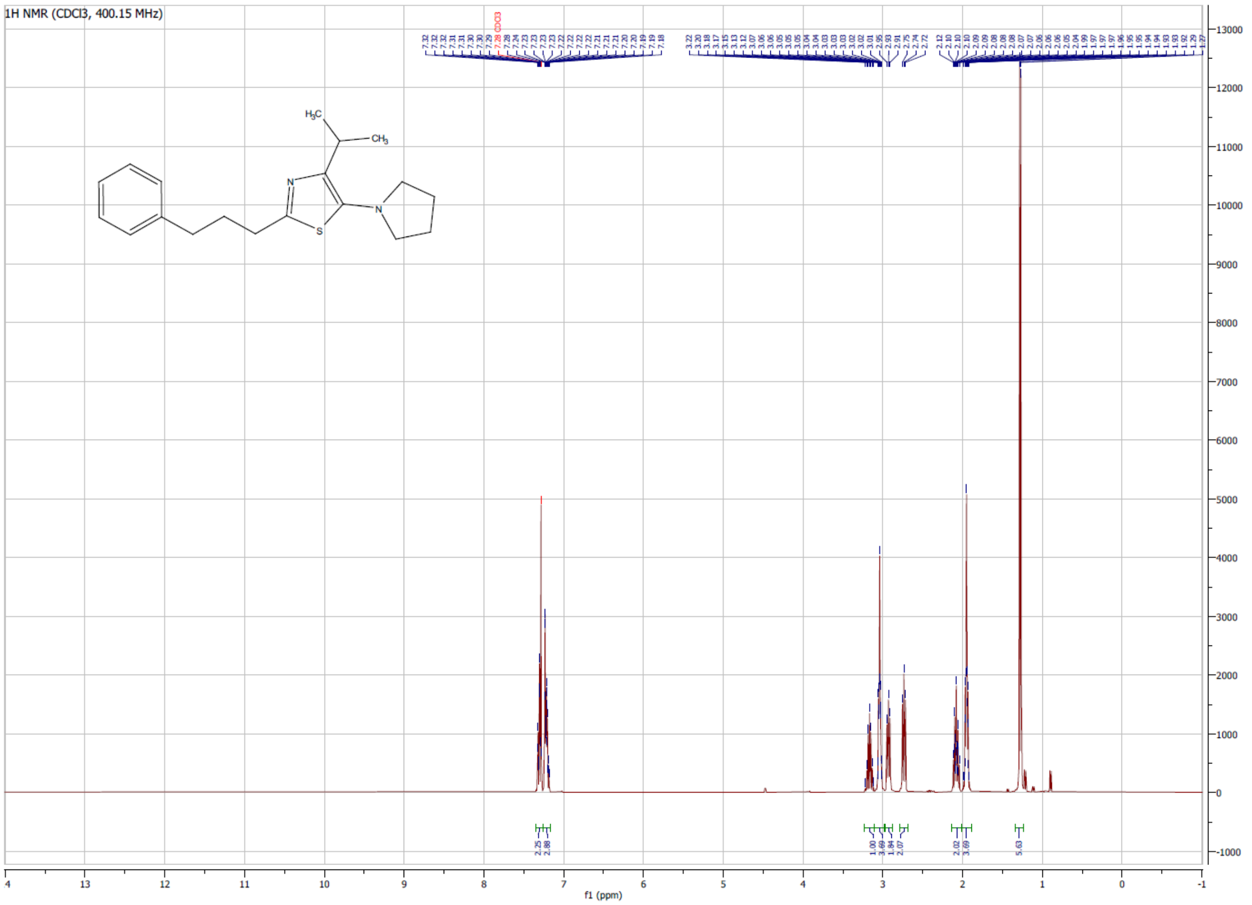

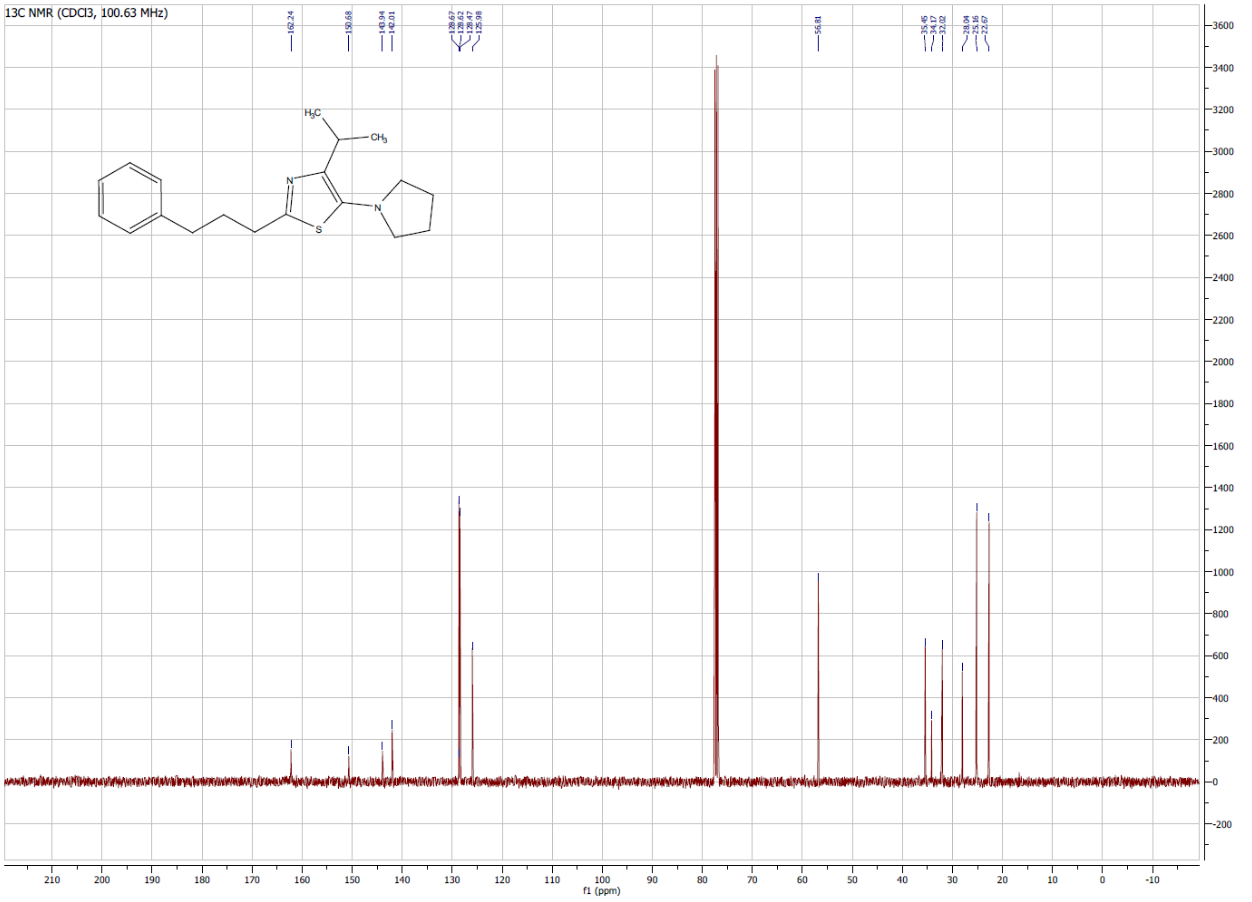

Figure S38. ^{1}H and ^{13}C NMR spectra of compound **16j**.

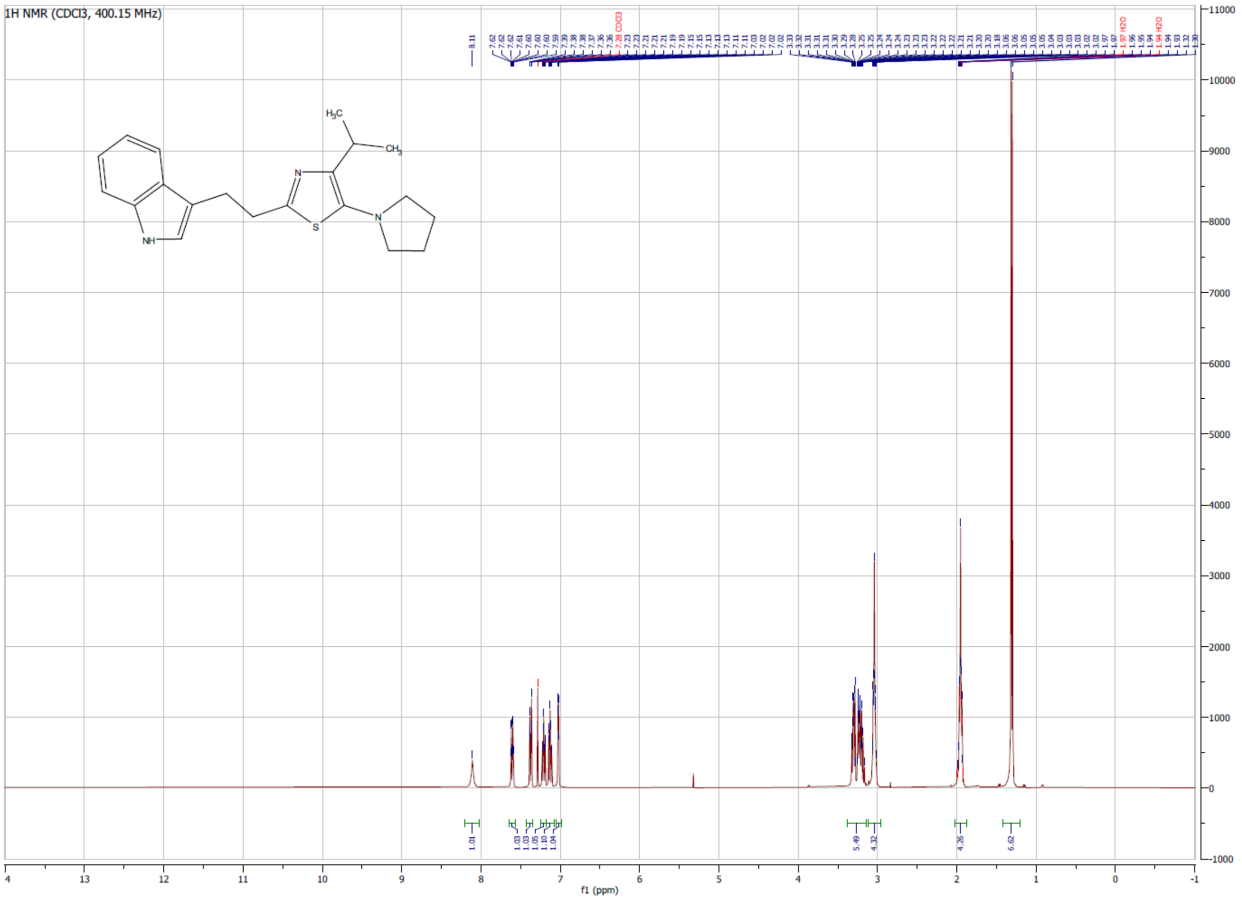

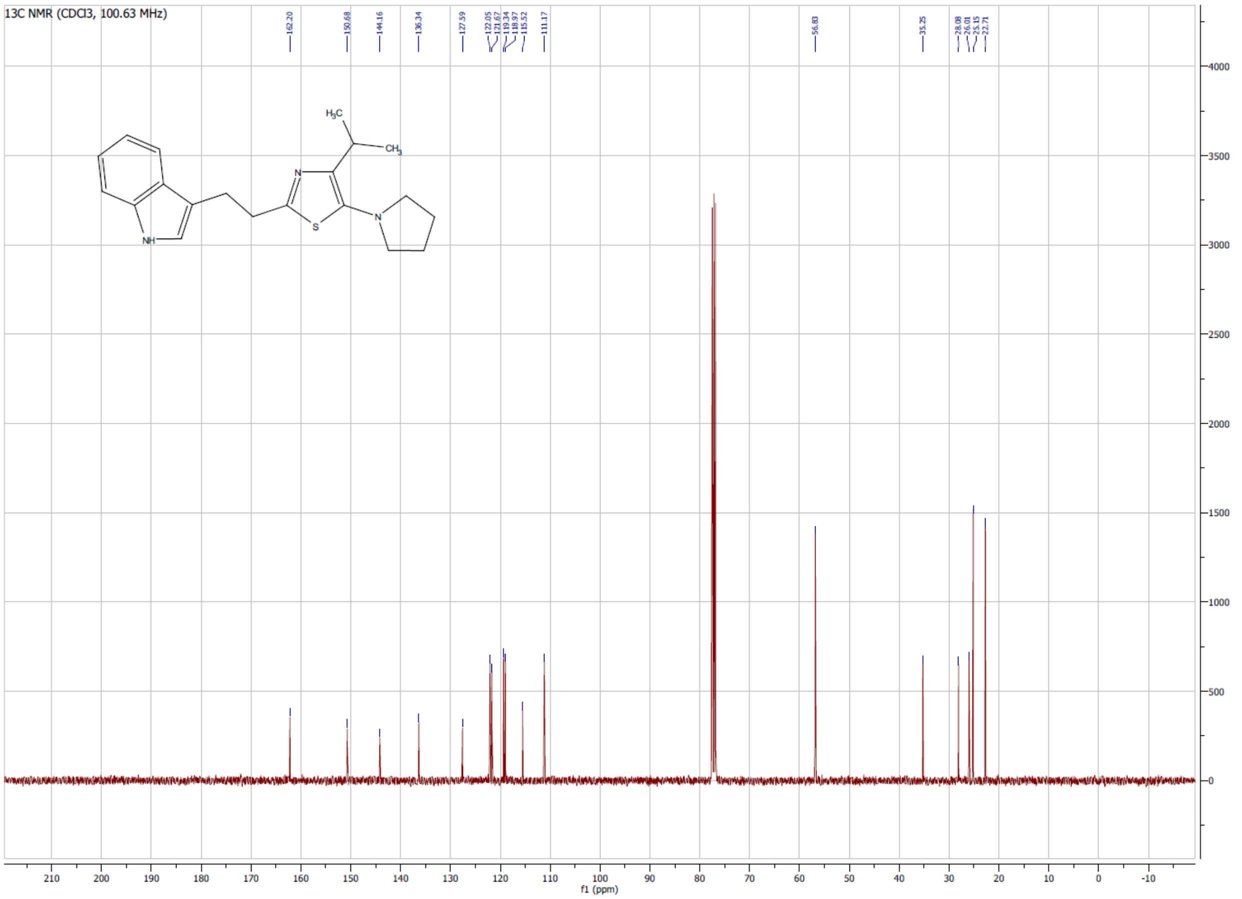

Figure S39. ^{1}H and ^{13}C NMR spectra of compound **16k**.

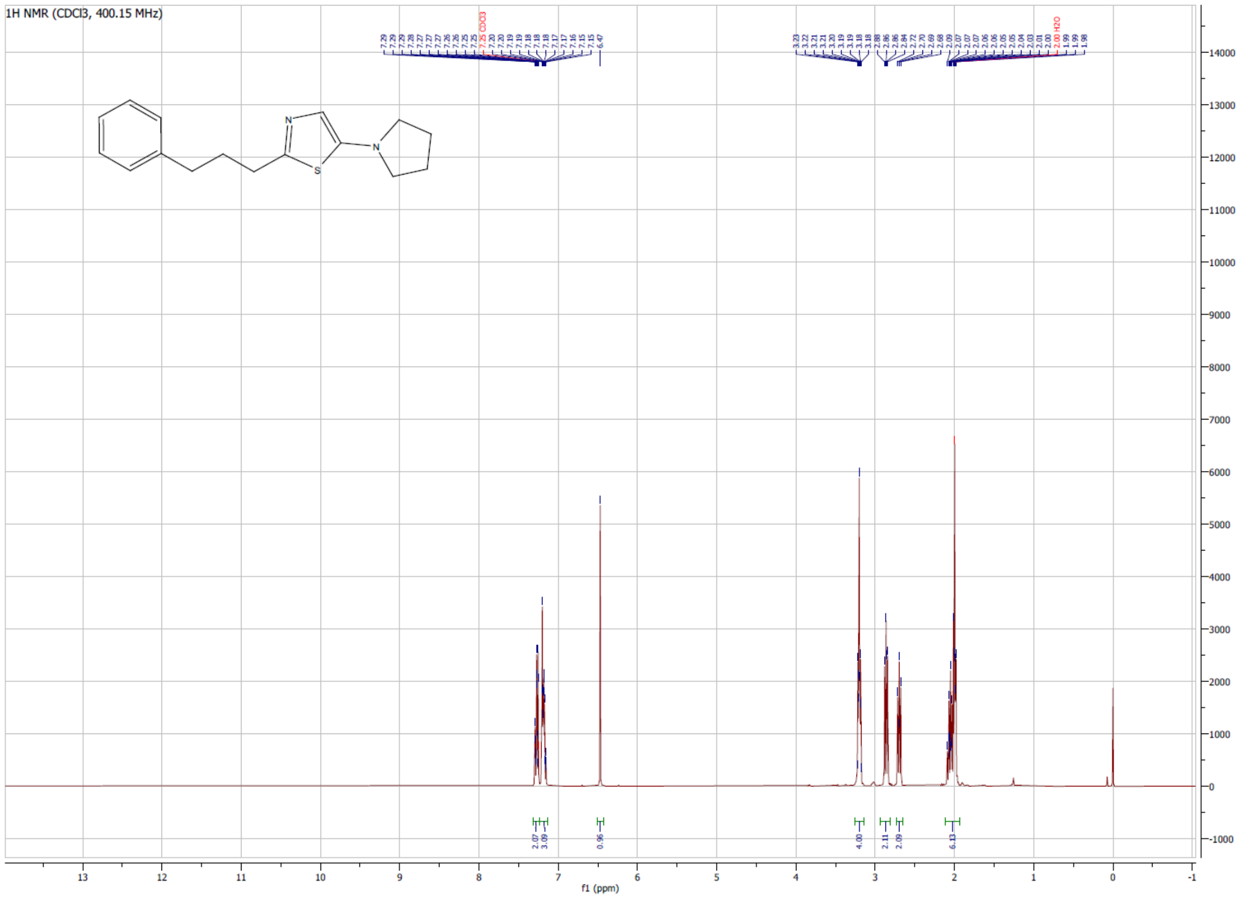

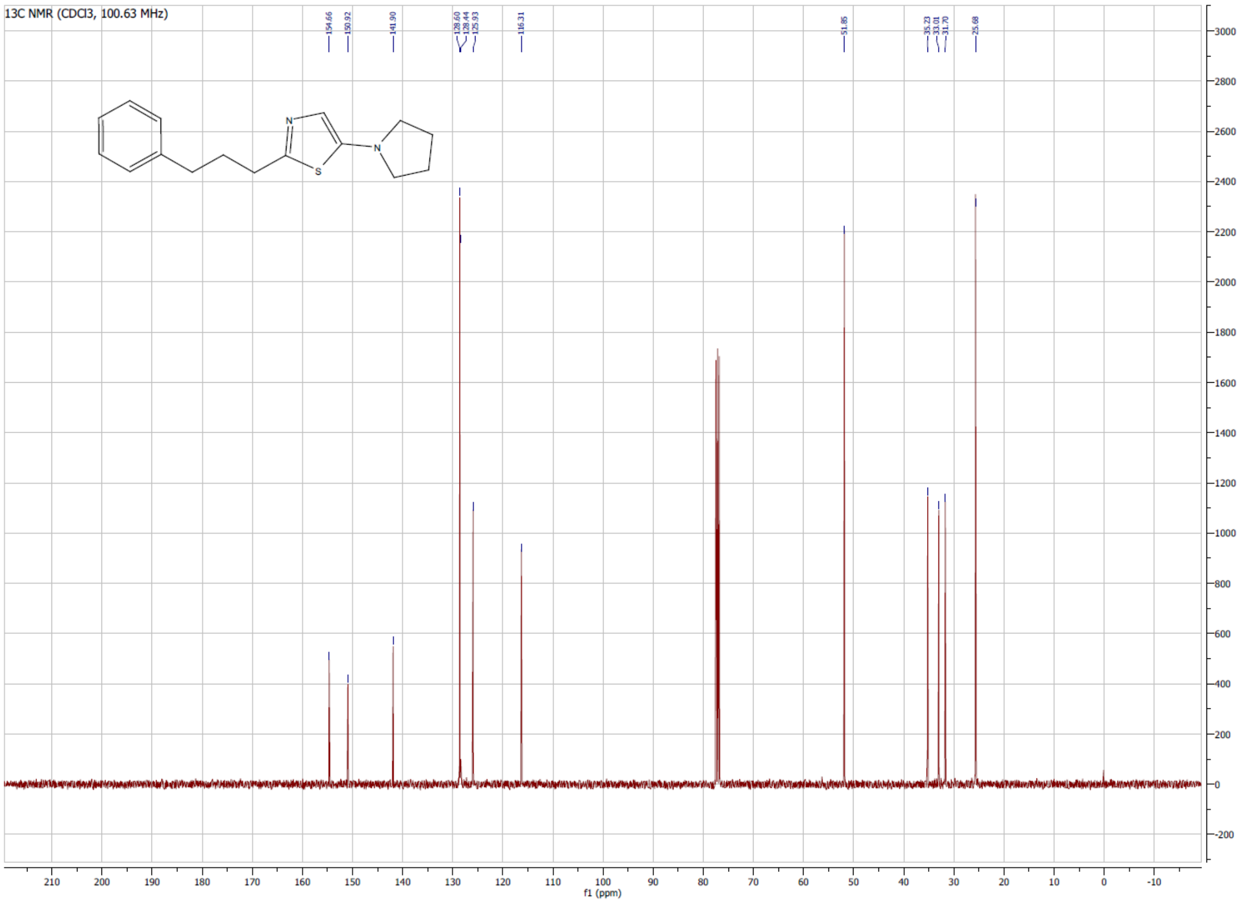

Figure S40. ^{1}H and ^{13}C NMR spectra of compound **16l**.

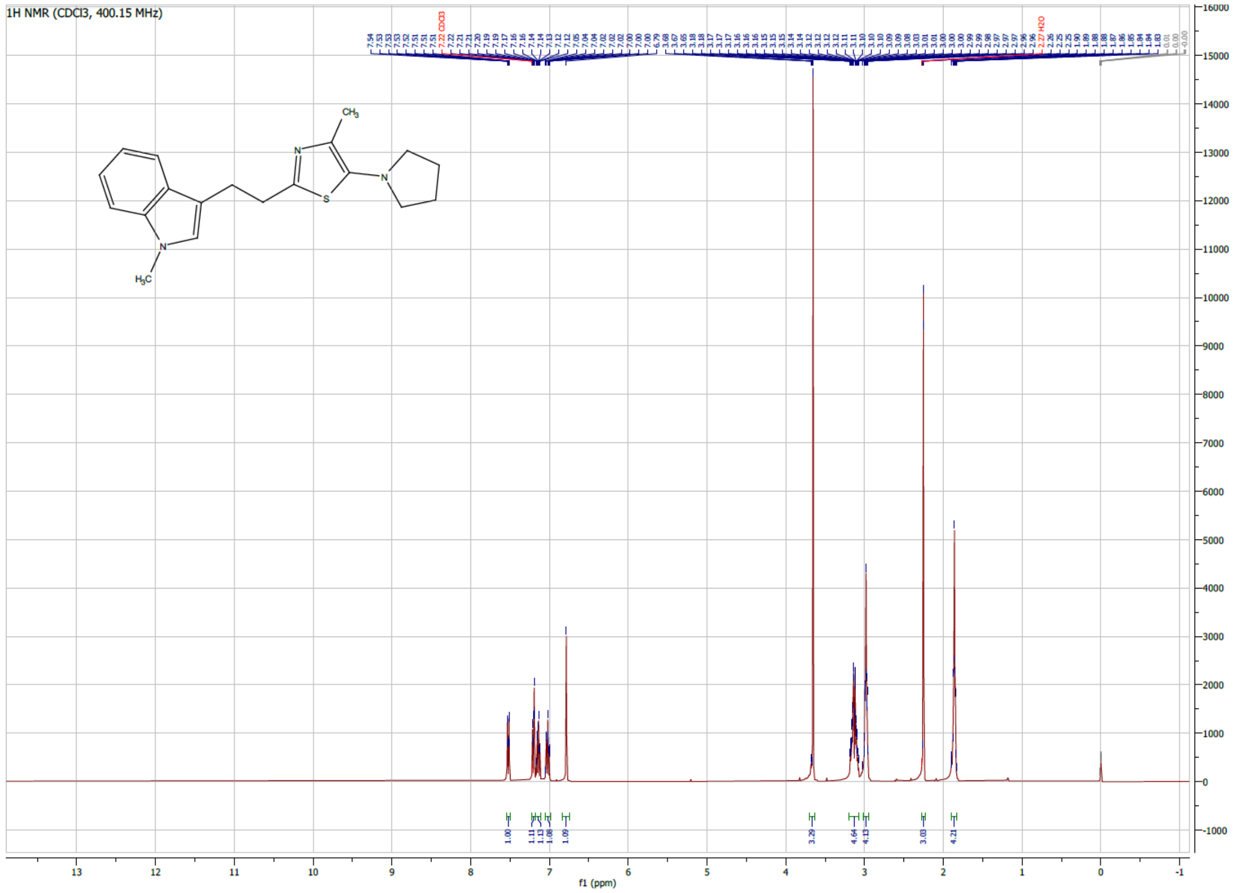

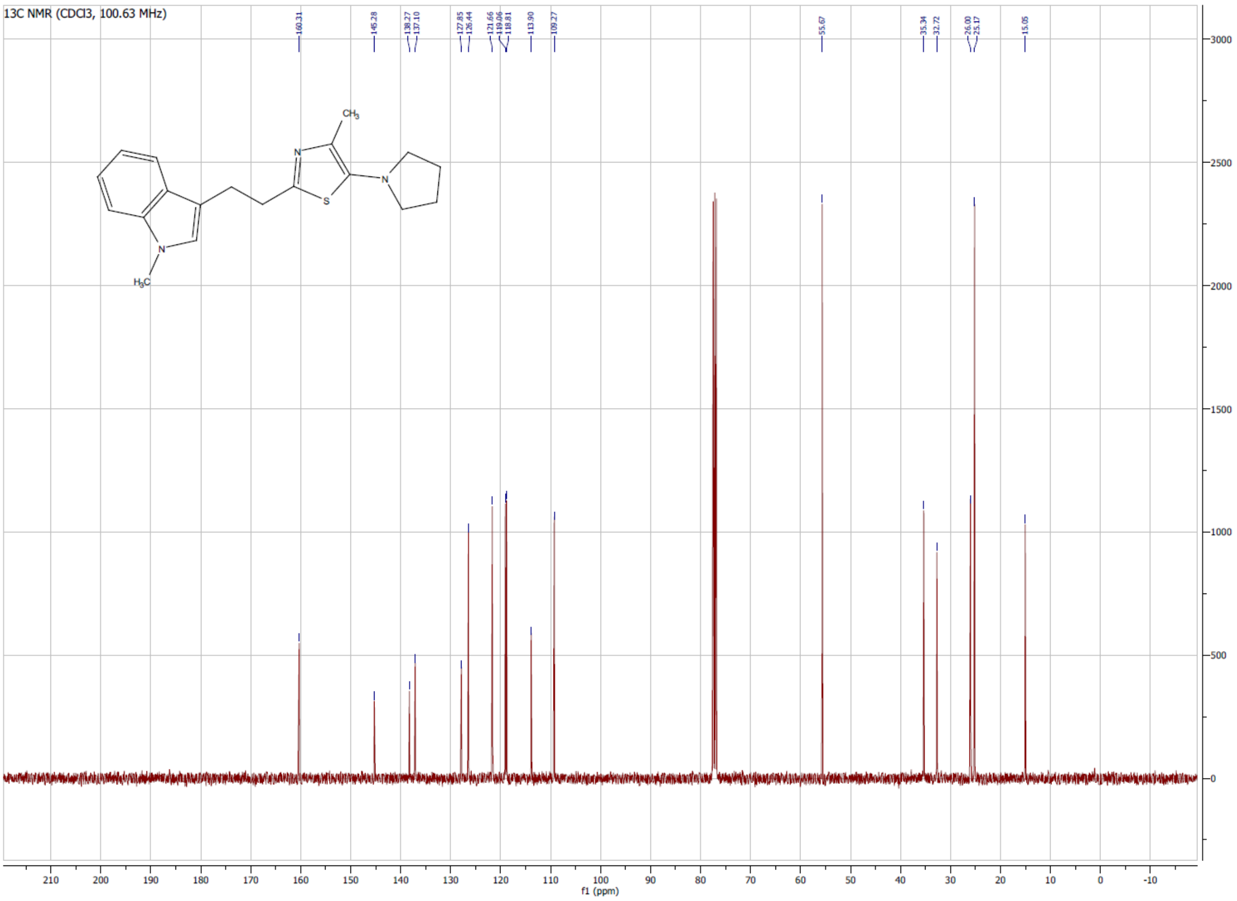

Figure S41. ^{1}H and ^{13}C NMR spectra of compound **17**.

Molecular Modelling Experimental Data

Crystal Structure Choice and Preparation

Several crystal structures (Table S1) were examined and used for comparison. The only available crystal structure of human PREP (hPREP) (PDB ID: 3DDU)[1] was chosen for a more detailed examination. It has a non-covalently bound peptide-like ligand and a relatively low resolution of 1.56 Å.

Table S1. Crystal structures of PREP used in this study.

Organism[a]	Ligand at active site	Resolution (Å)	PDB ID	Citation
Human	Peptide-like; non-covalent	1.56	3DDU	Haffner et al., 2008[1]
Porcine	ZPP; covalent	2.00	1QFS	Fülop et al., 1998[2]
Porcine	-	1.40	1QFM	Fülop et al., 1998[2]
Porcine[a]	KYP-2047; covalent	2.20	4AN0	Kaszuba et al., 2012[3]

[a]Expressed in E. coli.

The molecular modelling studies were primarily done using Schrödinger Maestro.[4] Proteins were prepared using Maestro's Protein Preparation Wizard with mostly default settings. During pre-processing, missing side chains were filled with Prime and heteroatom states were generated and selected at pH 7.4 with Epik. All small molecules except the active site ligands were removed. Hydrogen bond assignment was done with default settings at pH 7.4 using PROPKA.[5] Minimization was performed with default settings using force field OPLS3e.

Identifying potential binding sites

Possible binding sites of the crystal structure of hPREP (PDB ID: 3DDU) were searched for using Maestro's SiteMap tool using default settings (require at least 15 site points per reported site, more restrictive hydrophobicity, standard grid, and crop site maps at 4 Å from nearest site point).[4] The resulting sites were inspected visually and by comparing their calculated SiteScore.

Molecular Docking

Ligands were prepared for docking from their 2D structures with LigPrep using force field OPLS3e.[4] Docking was first done using glide at XP precision with van der Waals radii scaling factor set to 0.7. The grids for glide docking were generated with the center of the grid determined based on the position of the residues surrounding the site that was being docked to. Induced fit docking was done using Maestro's Induced Fit Docking protocol. The induced fit docking box was centered on the workspace ligand, the position of which was obtained from the Glide docking results.

Molecular Dynamics

The crystal structure of hPREP is missing two amino acids from one of the hinges (Thr426 and Gly429). In order to make it useable in molecular dynamics simulations, residues 425-433 were spliced in from a crystal structure of porcine PREP (PDB ID: 4AN0).[3] No significant changes to the predicted second binding site or overall protein structure were observed after this splice. Ligands were then docked into the predicted second binding site on the spliced structure using Glide docking to obtain the starting point for molecular dynamics simulations.[4] Molecular dynamics simulations were set up in using the Desmond system builder tool with mostly standard settings. No ions were

added and the force field OPLS3e was selected. Simulations were run with standard settings using Desmond. Simulations were first run for 50 ns, continued to 100 ns, and finally to 150 ns if the ligand remained in the binding site until the end of the previous 50 ns simulation. The results were analyzed visually and with the help of Desmond simulation interaction diagrams.

Molecular Modelling Supplementary Results

Docking to the active site

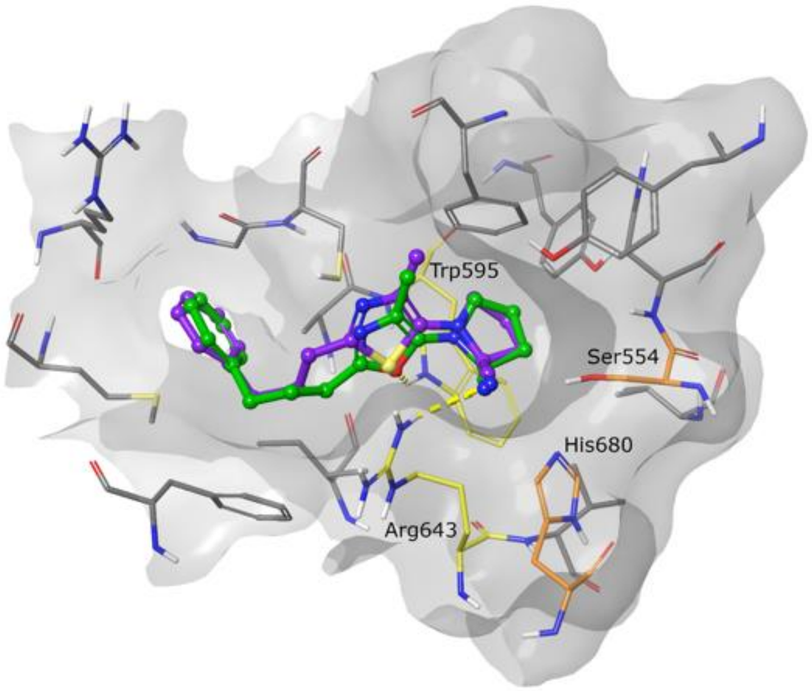

Figure S42. The oxazole HUP-55 (green) and the thiazole analogue **HUP-46** (purple) bound to the proteolytic active site (PDB ID: 3DDU).[1] Hydrogen bonds are shown as yellow dashed lines. The sulphur of the thiazole ring is unable to make the hydrogen bond made by the oxygen of the oxazole ring (to Trp595).

Docking to the new binding site

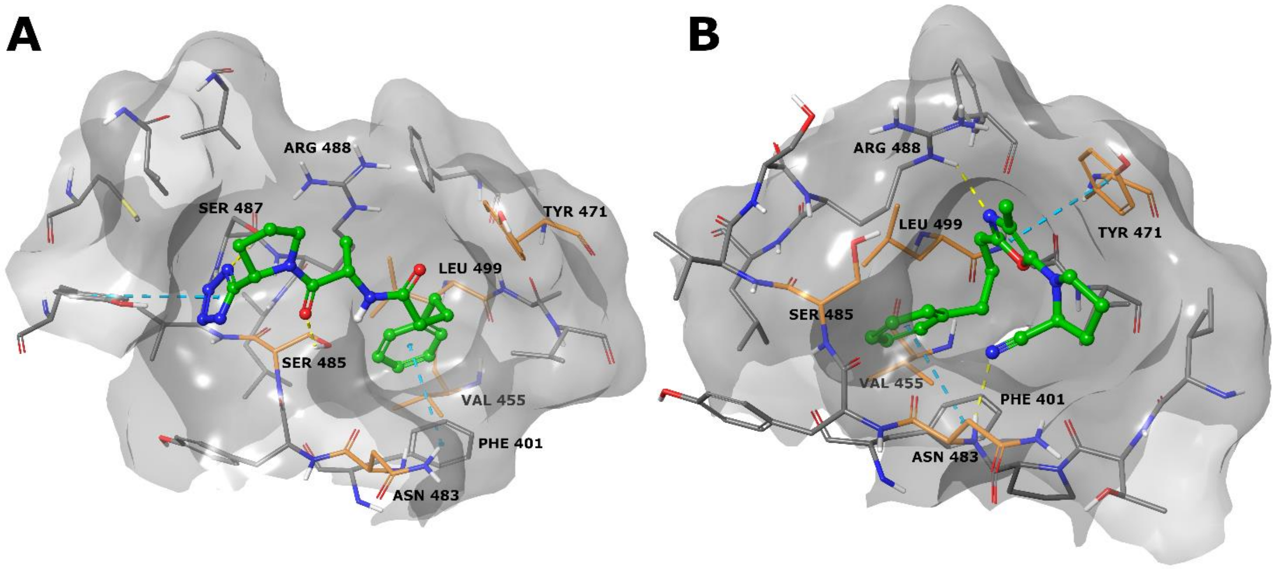

Figure S43. Induced fit docking poses for (A) HUP-28 and (B) HUP-55 at the postulated new binding site (PDB ID: 3DDU).[1] Hydrogen bonds are shown as yellow dashed lines and π-π stacking as blue dashed lines.

Conservation of the new binding site

The conservation of the site chosen for further investigation was evaluated by overlaying the crystal structure of hPREP with the other crystal structures in Table S1. The site appears very similar in all the crystal structures, with the side group of Arg488 being the most variable (Figure S44). In the chosen crystal structure (PDB ID: 3DDU), Arg488 is in such a position that the hydrophobic pocket of the predicted second binding site is more open than in other structures.

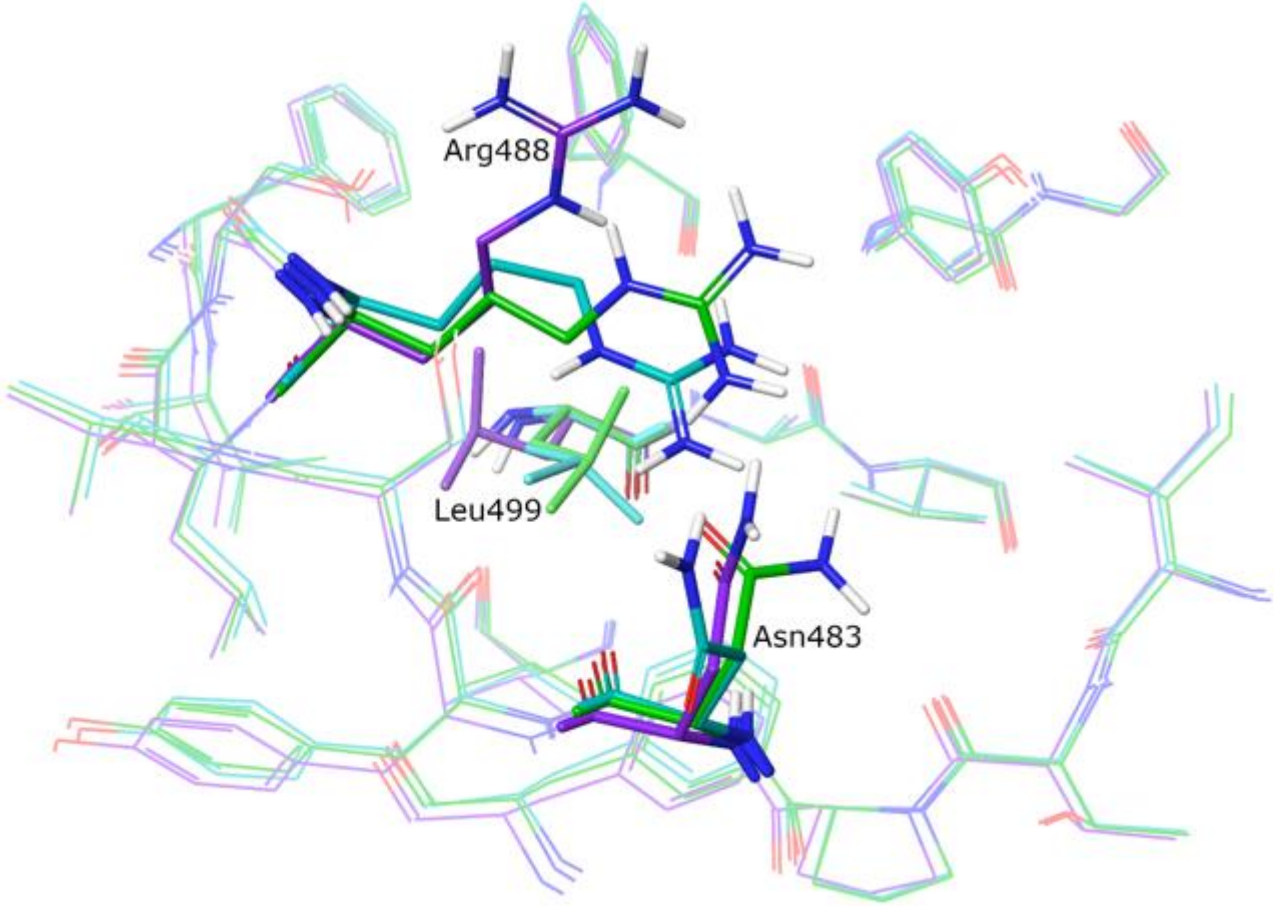

Figure S44. The predicted second binding site in crystal structures with PDB IDs 3DDU (purple),[1] 4AN0 (green),[3] and 1QFS (cyan).[2] The residues with most variability between the structures (Asn483, Arg488, and Leu499) are highlighted.

Molecular dynamics simulation interactions

Simulation interaction diagrams (SIDs) were created after each molecular dynamics simulation using Desmond.[4] The protein-ligand interactions are visualized in Figures S45 – S49. The interaction fraction represents the fraction of the simulation, where there was an interaction between the ligand and a certain amino acid residue. The fraction can be over 1 if the ligand has multiple simultaneous interactions to a residue.

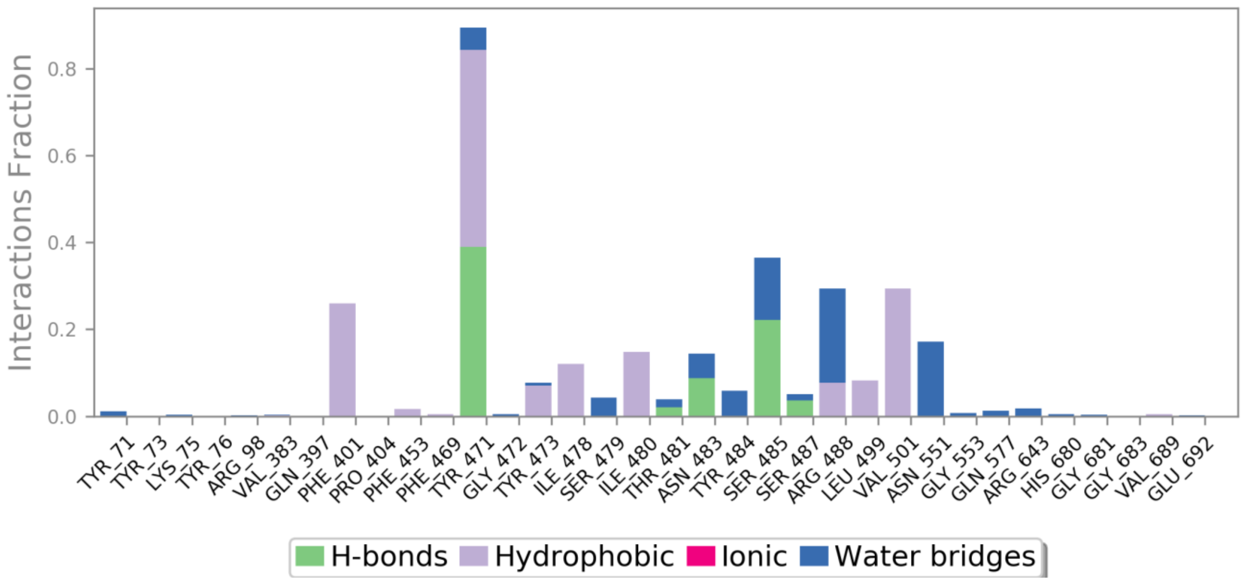

Figure S45. Protein-ligand contacts between HUP-55 and PREP during a MD simulation at time points 0 – 50 ns. HUP-55 was docked to the postulated new binding site before the simulation.

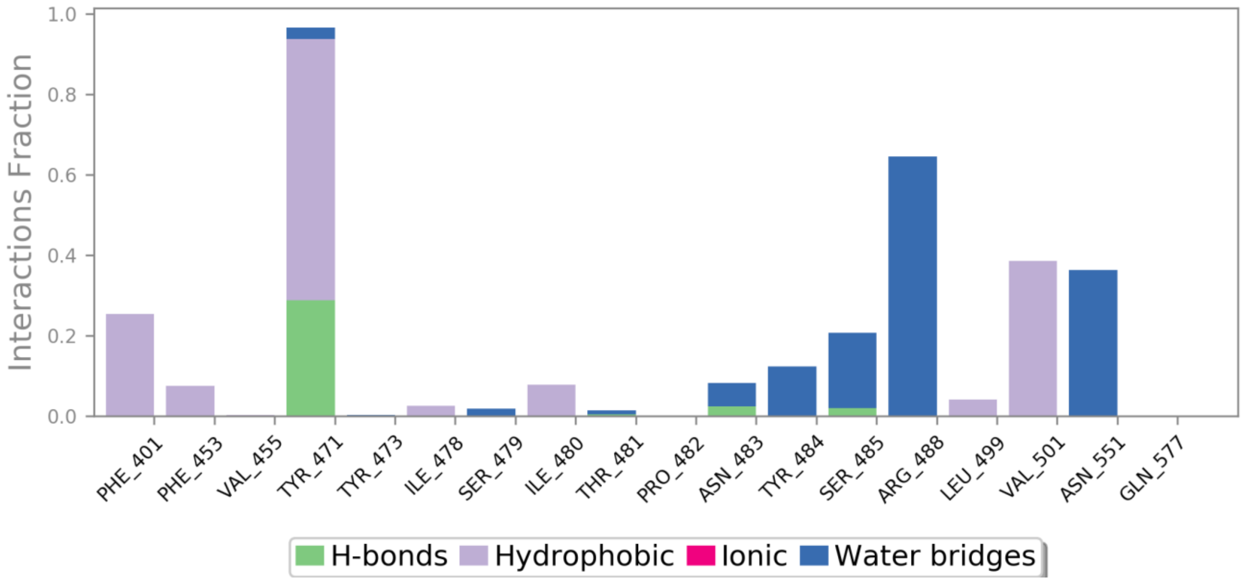

Figure S46. Protein-ligand contacts between HUP-55 and PREP during a MD simulation at time points 50 – 100 ns. The starting point for this simulation was the end of the first 50 ns simulation.

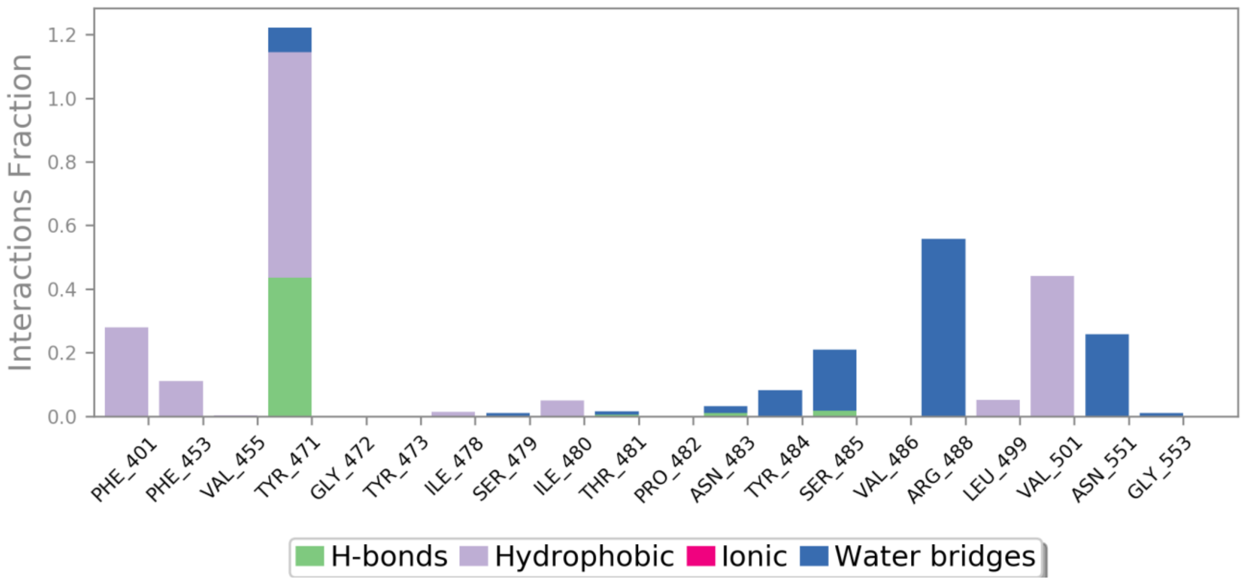

Figure S47. Protein-ligand contacts between HUP-55 and PREP during a MD simulation at time points 100 – 150 ns. The starting point for this simulation was the end of the first two 50 ns simulations.

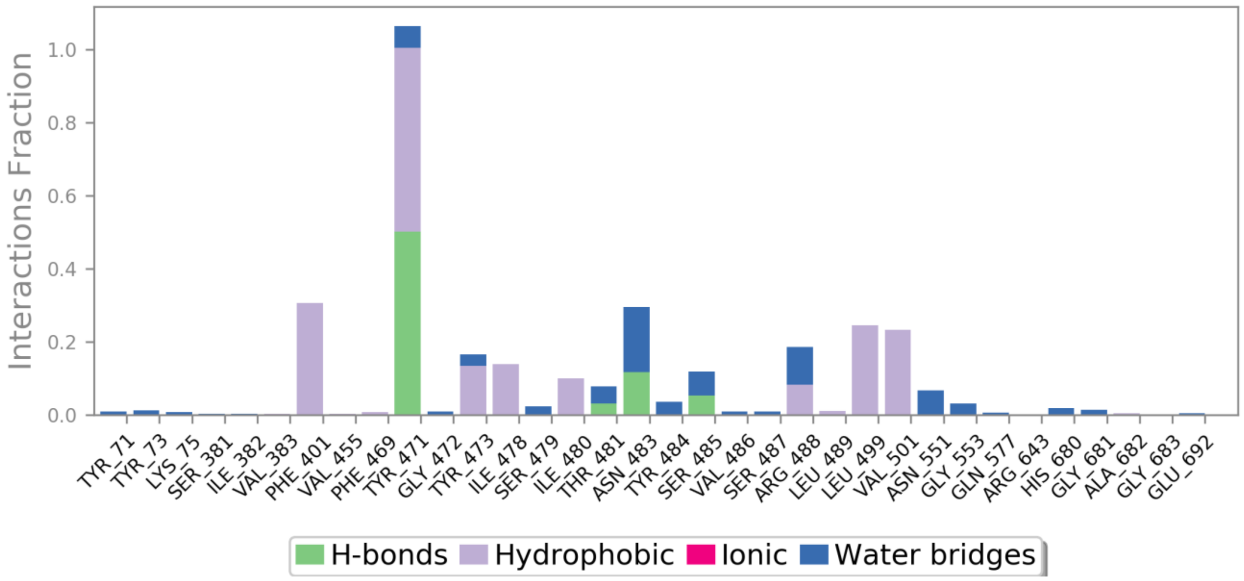

Figure S48. Protein-ligand contacts between **HUP-46** and PREP during a MD simulation at time points 0 – 50 ns. **HUP-46** was docked to the postulated new binding site before the simulation.

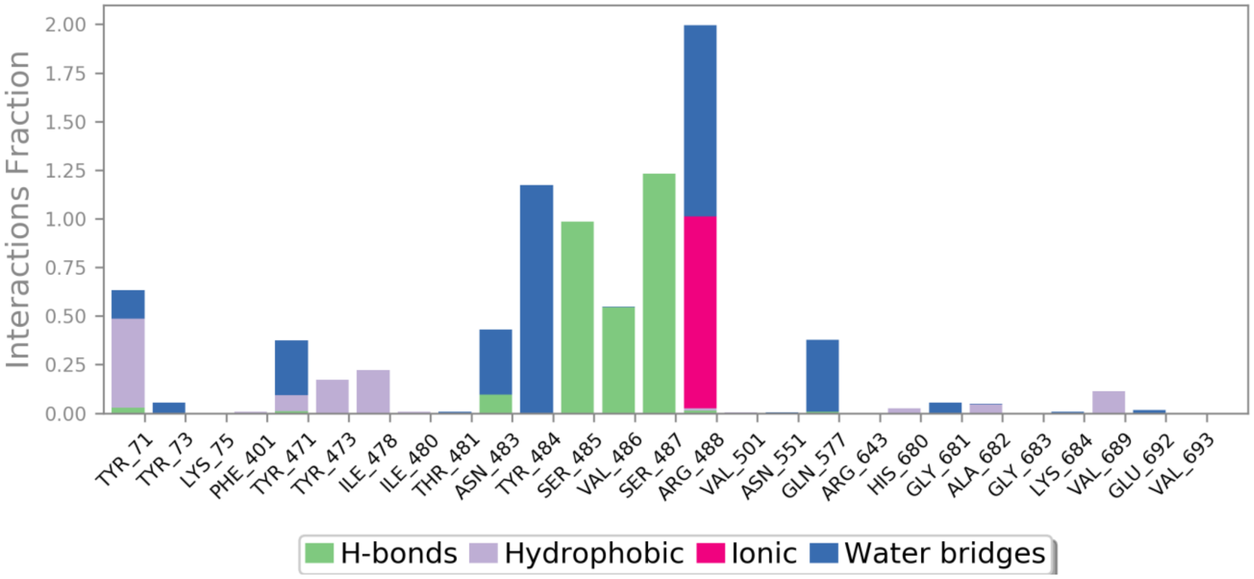

Figure S49. Protein-ligand contacts between HUP-28 and PREP during a MD simulation at time point 0 – 50 ns. HUP-28 was docked to the postulated new binding site before the simulation.

Covalent docking to the new binding site

Possible covalent binding to the Val455Cys and Leu499Cys mutants at the second binding site with analogues of HUP-28 and **7** are shown in Figure S50.

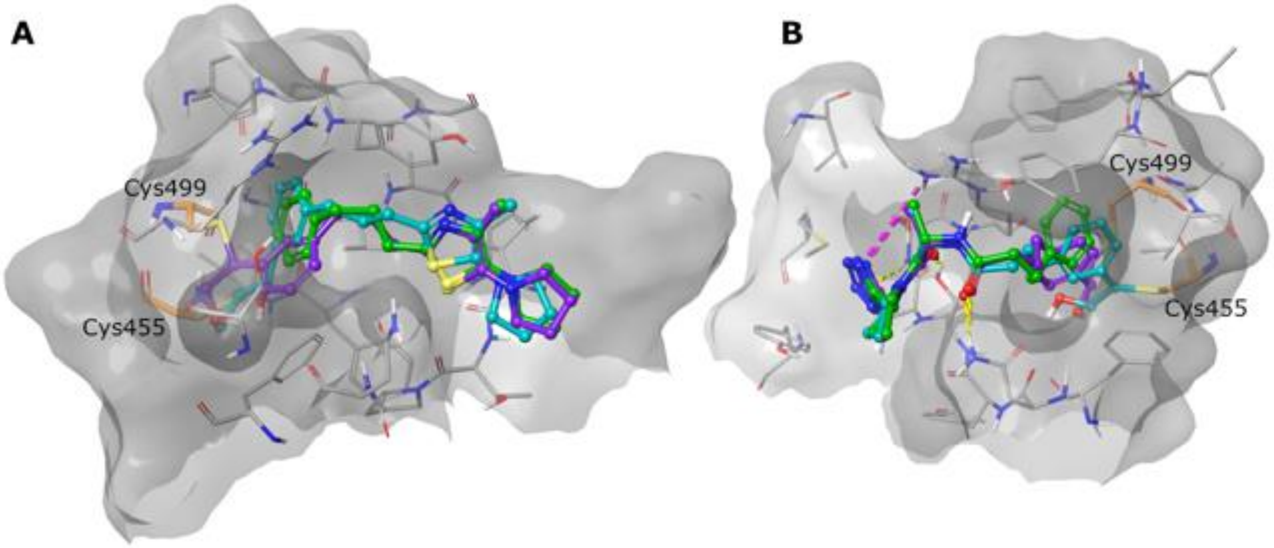

Figure S50. (A) Compound **7** (green) and (B) HUP-28 (green) bound to the unmutated second binding site (PDB ID: 3DDU),[1] overlayed with their epoxide-containing analogues bound covalently to the Val455Cys (cyan) or Leu499Cys (purple) mutated second binding site. Hydrogen bonds (yellow) and salt bridges (magenta) are shown as dashed lines.

Biological Experimental Data

Reagents

Reagents were purchased from Sigma-Aldrich (St. Louis, MO) if not otherwise specified. PREP ligands were diluted to cell culture medium from 100 mM stock in 100 % DMSO, and corresponding concentration of DMSO was used as a vehicle control.

Animals

C57BL/6JRccHsd mice were used for primary neuron collection and for HUP-46 brain penetration study. 10 weeks old male C57BL/6JRccHsd mice (*n* = 12) obtained from Envigo (The Netherlands) were used. The mice were singly housed in individually ventilated cages (Mouse IVC Green Line, Techniplast, Italy), kept under standard laboratory conditions (room temperature 23 ± 2 °C, 12 h light/dark cycle), and had access to food (Teklad 2016, Envigo) and irradiated tap water *ad libitum*. Animal experiments were conducted according to the 3R principles of the European Union directive 2010/63/EU regarding the care and use of experimental animals and following the local laws and regulations [Finnish Act on the Protection of Animals Used for Scientific or Educational Purposes (497/2013), Government Degree on the Protection of Animals Used for Scientific or Educational Purposes (564/2013)]. The experimental protocols were authorized by the National Animal Experiment Board of Finland (license, ESAVI/42235/2019). In addition, the animal experiments were reported in accordance with ARRIVE (Animal Research: Reporting of In Vivo Experiments) guidelines.

DNA Constructs

Preparation of wt human pAAV1-EF1α-PREP (hPREP; #59967, Addgene, Brandon Harvey Lab; RRID: Addgene_59967) and pAAV-EF1α-Ser554Ala PREP (S554A PREP; #59968, Addgene, Brandon Harvey Lab; RRID: Addgene_59968) is described earlier in Savolainen et al.[6] Additional PREP mutants (Asn483Ala, Leu499Cys, Tyr471Ala, Ser485Ala, Val455Cys) were prepared by using a site-directed mutagenesis of wt PREP (QuikChange II XL, #200521, Agilent Technologies). PREP mutant constructs were prepared by using site-directed mutagenesis of wt PREP (QuikChange II XL, #200521, Agilent Technologies) as described in Svarcbahs et al.[7]

Mouse primary cortical neuron cultures

Pure primary mouse cortical neurons were obtained from C57BL/6JRccHsd mice (P1). Cortical tissue was dissected out and dissociated in ice-cold Hank's Balanced Salt Solution (HBSS) medium (#14175, ThermoFisher Scientific) containing trypsin (0.25 mg/mL) and DNAse (5U/mL). After centrifugation, cortical neurons were re-suspended in Neurobasal Medium (NBM; #21103049, ThermoFisher Scientific) containing 2% (v/v) B27 (#17504044, ThermoFisher Scientific), 1% (v/v) penicillin-streptomycin solution, 100 µg/mL Primocin (#ant-pm-1, InvivoGen, Toulouse, France) and 2 mM glutamine (#25030081, ThermoFisher Scientific) and seeded onto poly-L-lysine (#p4707, Sigma)-coated cell culture plates at an appropriate density (40,000 cells/well to 96-well plates). After 24 h seeding, the medium was half-changed and neurons were maintained at 37 °C in a 5% CO_2 incubator. The neurons were cultured for 7 days for experimental use.

Cell Viability Assay

The impact of compounds on cell viability was assayed by the end of incubation period by using standard LDH and MTT assays as described earlier in Svarcbahs et al.[8] and Myöhänen et al.[9] HEK-

293, SH-SY5Y cell cultures and mouse primary neurons were seeded onto 96-well plates (10,000 cells/well) and incubated overnight prior to αSyn transfection and treatment with OS or lactacystin stress +/- study compounds as described above. After 24 h or 48 h (mouse primary neurons) incubation with study compounds, the mitochondrial dehydrogenase activity (MTT) tests were performed.

Close Relative Enzyme Specificity Assay

The recombinant proteins were prepared and purified as described in Van der Veken et al.[10]
PREP: Human recombinant PREP was expressed in BL21(DE3) cells and purified using immobilized Co-chelating chromatography (GE healthcare), followed by anion-exchange chromatography on a 1 ml Mono Q column (GE healthcare).
Fibroblast activating protein (FAP): A gateway-entry clone for human FAP was purchased from Dharmacon (Accession number DQ891423) and the human secretion signal was replaced with the HoneyBee mellitin secretion signal. For transfection and expression of FAP in Sf9 insect cells the C-terminal BaculoDirect kit from LifeTechnologies was used. The enzyme was purified from the supernatant of the insect cells using immobilized Ni-chelating chromatography (GE healthcare, Diegem, Belgium), followed by anion-exchange chromatography using a 1 mL HiTrap Q and size exclusion chromatography using the Superdex 200 column (GE healthcare, Diegem, Belgium).
Dipeptidyl peptidase 4 (DPP4): DPPIV was purified from human seminal plasma as described previously in de Meester et al.[11]
Dipeptidyl peptidase 9 (DPP9): Gateway-entry clones for human DPP9 were purchased from Dharmacon (Accession number DQ892325). For transfection and expression of DPP9 in Sf9 insect cells the N-terminal BaculoDirect kit from LifeTechnologies was used. The enzyme was purified using immobilized Ni-chelating chromatography (GE healthcare, Diegem, Belgium), followed by anion-exchange chromatography using a 1 mL Mono Q (GE healthcare, Diegem, Belgium).
Dipeptidyl peptidase 2 (DPP2): Recombinant human DPP2 was purchased from R&D Systems (#3438-SE-010).

Specific assays

PREP: Initial screening of the **HUP-46** was done using N-succinyl-Gly-Pro-7-amino-4-methylcoumarine (AMC) (Bachem) as the substrate at a concentration of 250 µM at pH 7.4 (0.1 M K-phosphate, 1 mM EDTA, 1 mM DTT). **HUP-46** was tested at two concentrations, 1 and 10 µM, being the final concentration in the well. **HUP-46** were pre-incubated with the enzyme for 15 minutes at 37 °C, afterwards the substrate was added and the velocities of AMC release were measured kinetically at λex= 380 nm, λem= 465 nm for at least 10 minutes at 37 °C. Measurements were done on the Infinite 200 (Tecan Group Ltd.) and the Magellan software was used to process the data.
FAP: Initial screening of the **HUP-46** was done using Z-Gly-Pro-AMC (Bachem) as the substrate at the concentration of 50 µM at pH 8 (0.05 M Tris-HCl buffer with 0.1% glycerol, 1 mg/ml BSA and 140 mM NaCl). **HUP-46** were pre-incubated with the enzyme for 15 minutes at 37 °C, afterwards the substrate was added and the velocities of AMC release were measured kinetically at λex= 380 nm, λem= 465 nm for at least 10 minutes at 37 °C. Measurements were done on the Infinite 200 as above.
DPP4 and DPP9: Ala-Pro-paranitroanilide (pNA) was used as the substrate at the respective concentrations of 25 µM (DPP4) or 150 µM (DPP9) at pH 7.4 (0.05 M HEPES-NaOH buffer with 0.1 % Tween-20, 0.1 mg/mL BSA and 150 mM NaCl). **HUP-46** was pre-incubated with the enzyme for 15 minutes at 37 °C, afterwards the substrate was added and the velocities of pNA release were

measured kinetically at 405 nm for at least 10 minutes at 37 °C. Measurements were done on the Infinite 200 as above.

DPP2: Lys-Ala-pNA was used as the substrate at the concentration of 1 mM at pH 5.5 (100 mM NaAc, 10 mM EDTA, 14 μg/ml aprotinin). Similar as above, **HUP-46** was tested at two concentrations (1 and 10 μM), and preincubated for 15 min at 37 °C. The substrate was added and the velocities of pNA release were measured kinetically at 405 nm for at least 10 minutes at 37 °C. Measurements were done on the Infinte 200 as above.

Activity-Based Protein Profiling

Competitive activity-based protein profiling ABPP was performed by using PREP and mutant PREP transfected HEK-293 PREP knock-out cell (PREP-KO) lysates (Asn483Ala, Leu499Cys, Tyr470Ala and Ser485Ala PREP mutants) to visualize the selectivity of inhibitors towards PREP and against other serine hydrolases in cell and tissue proteomes with active site serine-targeting fluorescent fluorophosphonate probe TAMRA-FP (ActivX Fluorophosphonate Probes, ThermoFisher Scientific Inc., cat#88318, Rockford, IL, USA). Cell lysates and tissue homogenates (1,25 mg/ml) were pre-treated for 1 h with DMSO or a specific PREP inhibitor (KYP-2091, KYP-2112, **HUP-46**) (from 100 pM to 10 μM, depending on the inhibitors potency) after which TAMRA-FP (2 μM) incubation was conducted for 1 h at room temperature to label active serine hydrolases. The reaction was quenched by adding 2× gel loading buffer, after which 0,25 μg protein was loaded per lane and the proteins were resolved in 10 % SDS-PAGE together with molecular weight standards. TAMRA-FP labelled proteins were visualized by ChemiDoc™ MP imaging system (BIO-RAD, Hercules, CA, USA) with Cy3 blot application (602/50, Green Epi, Manual Exposure 10s–120s). The OD was measured by using ImageJ as above (WB), and the PREP signal was correlated with a serine protease signal showing equal signal between the rows.

Recombinant Human PREP Mutant Protein Production and Purification

Recombinant human wild type (wt) PREP, Asn483Ala and Leu499Cys PREP-enzymes were purified in a two-step purification procedure. Asn483Ala and Leu499Cys PREP were prepared by Genscript (Riswijk, The Netherlands) based on wt hPREP.[12] First HIS-tagged proteins were expressed in *E.coli* BL21 (DE3) cells. The cells expressing desired plasmids (pET46 EK/LIC-hPREP plasmid backbone) were plated on LB-agar-ampicillin plate and one colony was picked for 15 ml of TB-media (1.2% peptone, 2.4% yeast extract, 0.4% glycerol, 17mM KH_2PO_4 and 72mM K_2HPO_4) with 100 μg/ml ampicillin. Pre-cultures were incubated in a shaking incubator at 37°C and 250 rpm overnight. 5 ml of preculture were transferred to 1000 ml baffled flasks, each containing 330 mL TB-medium and 100 μg/ml ampicillin. Nine bottles were used (about 3 l in total) and cultures were allowed to grow at 37°C. When the OD_{600}-value reached 0.4 (after approx. 3.5 hours), ethanol was added to a final concentration of 2 % and the cultures were incubated at 25 °C for approx. one hour to reach OD_{600}-value of 0.6. The production of protein was initiated using isoprolylthio-galactoside at a concentration of 1 mM and incubated overnight at 25 °C. The following day, cells were collected by centrifugation for 30 min at 3000 x g and 4°C, followed by resuspending of the pellets at 40 ml/liter of culture in binding buffer (20 mM Tris, 5 mM imidazole, 330 mM NaCl, pH 7.3 and 1 mM TCEP). Cells were lysed by freezing the samples in liquid nitrogen, followed by thawing on ice and sonication (1 s sonication, 2 s pause, and total sonication 60 s). Samples were centrifuged at 4°C and 21 000 g for 2x60 min to get rid of all cell debris.

The lysate was purified using an Äkta Pure chromatography system (Cytiva). After loading of the cobalt (0.1 M $CoCl_2$) onto the column (HiTrap Chelating HP® 5 mL column, GE Healthcare), the

column was washed with MQ H_20 and equilibrated with binding buffer. 90 ml of the lysate was loaded on a series of 2 5 ml columns. The columns were washed with 5 column volumes (CV) of binding buffer and 3 CVs of wash buffer (20 mM TRIS-HCl, 15 mM Imidazole, 330 mM NaCl, pH 7.3) to get rid of unspecific binding. Next, elution was performed by eluting first with 100 mM imidazole in 1.5 ml fractions, followed by a second elution step with 300 mM imidazole, similarly in 1.5 ml fractions. For wt and Leu499Cys PREP, washing was done with 30 mM imidazole and elution with 300 mM imidazole, but more pure product and better yield was acquired for the Asn483Ala mutant with the aforementioned procedure since PREP sometime eluates already with washing step if the buffer has 30 mM concentration of imidazole. Resulting fractions were pooled according to PREP activity, and dialyzed overnight in buffer containing 20 mM TRIS-HCl, 1 mM EDTA and 5 mM DTT at pH 7.3 to get rid of imidazole. After dialysis, the sample was centrifuged at 4°C, 21 000 g for 60 min to get rid of precipitated protein impurities.
The second purification step was performed with anion exchange chromatography using a 1 ml HiTrap Q column (GE healthcare). Column was first washed with 2 CV of 2 M NaCl, 4 CVs of 1 M NaOH and 3 CV of 2 M NaCl followed by washing with MQ H20 and equilibration with 5 CVs of start buffer (20 mM TRIS-HCl, 1 mM EDTA and 5 mM DTT, pH 7.3). Sample was loaded to the column and washed with 4 CVs of start buffer followed by elution with 30 CV gradient to 500 mM NaCl (end buffer 20 mM Tris-HCl pH 7.3, 1 mM EDTA, 5 mM DTT, 500 mM NaCl). 1.5 ml fractions were collected and PREP-activity measurements, SDS-page with coomassie stain and anti-histag western blot were performed on the fractions to evaluate Prep purity and quality.

Isothermal Titration Calorimetry

Recombinant human wild type (wt), Asn483Ala and Leu499Cys PREP were dialyzed at approx. 1000 µL of sample against 1 L buffer (100 mM Tris pH 7.4, 1 mM EDTA, 3 mM DTT at pH 7.4). An initial dialysis step for 2 hours, was followed by transfer to a new buffer volume and additional dialysis overnight. After dialysis, concentration was determined using A280 absorbance measurements (ε= 127000) in a low-volume cuvette with a Spectramax-384-Plus reader (Molecular Devices) at a 1/20 or 1/25 dilution. Dialysis buffer was used as isothermal titration calorimetry (ITC) assay-buffer.
Tested PREP ligands were in 100 mM DMSO stock. This stock was diluted further to 10 mM or 2 mM DMSO stock depending on the dilution scheme needed for ITC and diluted further in the buffer. Prep and inhibitor solutions were matched in composition, by using dialysis buffer for performing dilutions and matching the DMSO composition of the PREP-solutions. The final DMSO concentration in solutions varied from 0.24 % to 4.5 % depending on the concentration of tested compound. Before the preparation of the titration samples, the buffer solution was equilibrated to room temperature and the buffer and DMSO were ultrasonicated to remove air bubbles. The titration samples themselves were not sonicated since the effect of sonication on the integrity of the samples is not known. All titrations were performed using the Peaq-ITC (Malvern Pananalytical, Malvern, UK). For every titration, the same standard set-up was used where the initial spacing was 180 s, spacing between the injections was 150 s, Differential power (DP) was 5 µcal/s, stirring was 750 rpm and feedback was set at 'High'. Injection volume was 2.5 µl and number of injections was 15, excluding the first 0.4 µL injection. After each titration, the sample cell and syringe were cleaned with the standard cleaning procedure using milliQ H_20, 14 % decon and MeOH. Control titrations for each inhibitor were performed in the absence of PREP, by injecting inhibitor in a buffer-filled cell, using the exact same conditions as for the experimental runs. All conditions were run at least in triplicate. Thermograms were analyzed using the Microcal Peaq-ITC Analysis software, using the 'one set of sites' binding model, by including the corresponding control titration.

LC-MS detection of HUP-46 in the mouse brain

I.p. injection of **HUP-46** (10 mg/kg) was given to C57BL/6JRccHsd mice (n=3/timepoint), and the mice were deeply anesthetized with sodium pentobarbital anaesthesia (i.p. 200 mg/kg), perfused briefly with PBS and the brains were removed at 0, 30, 60 and 120 min after the injection. The mouse brain tissue and cells were disrupted with ball mill followed by freeze-thaw cycle integrated with ultra-sonication. The samples were extracted with 500 µl MeCN twice, evaporated to dryness, and reconstituted in 200 µl of MeCN. The chromatographic separation was performed in Waters Acquity UPLC BEH C18 column (Ø1.7 µm, 2.1 mm x 50 mm) in 40 °C, and with a flow rate of 0.6 ml/min. The mobile phase consisted of 0.1% formic acid in MQ H_2O (A) and 0.1% formic acid in MeCN (B). The linear gradient started from 5% B and increased to 95% B in 9 min, then switched back and left to stabilize for 1min. Exion UPLC - 6500+ QTRAP/MS instrumentation (Sciex) was used for quantification of **HUP-46** with following transition in Multiple Reaction Monitoring (MRM) method. Transition 312→285 was used for **HUP-46**. The concentration of **HUP-46** was quantified using calibration curve with corresponding standard, and the data of the brain samples was normalized to the fresh weight (FW).

Statistical Analysis

All experiments were done at least in triplicate. Data are expressed as mean values ± standard error of the mean (mean ± SEM), and negative control average was set as 100% on each biological assay to reduce variability between repeats. To analyze the statistical differences between groups, 1-way analysis of variance (ANOVA) was followed by Dunnett’s post hoc comparison if ANOVA assay gave statistical significance ($p < 0.05$). In all cases, $p < 0.05$ were considered to be significant. Statistical analysis was performed using PRISM GraphPad statistical software (version 7.0, GraphPad Software, Inc.).

Biological Supplementary Results

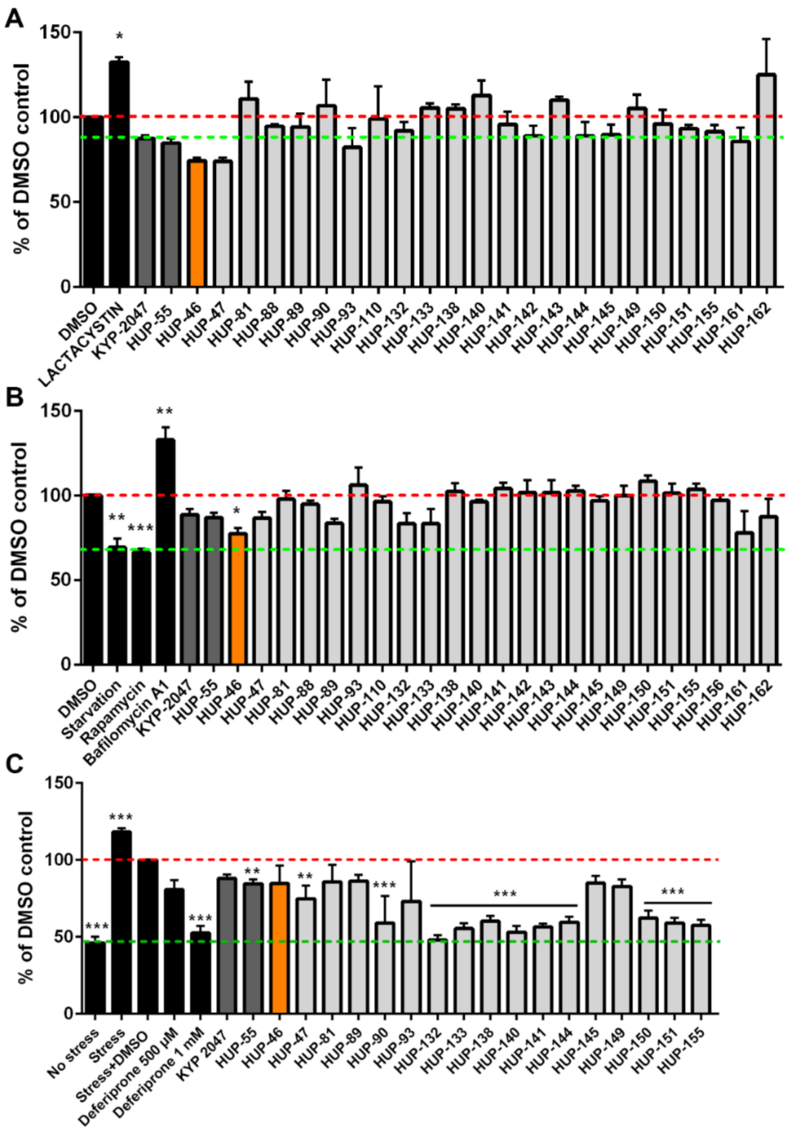

Figure S51. (A) Luminescence signal of αSyn-αSyn interaction detected by protein-fragment complementation assay after 48 h transfection and 4 h treatment in N2A cells. Lower signal compared to control indicates decreased αSyn-αSyn interaction. 10 µM concentration for test compounds were used. 10 µM proteasome inhibitors lacatacystin and MG-132 were used as a control for increased αSyn dimerization. Red line indicates the level of DMSO control and green hatched line indicates the level of reference compound KYP-2047. (B) GFP signal after 24 h treatment in GFP-LC3-RFP HEK-293 cells. Lowe signal indicates increased autophagic flux compared to control. 10 µM concentration for test compounds were used. 500 nM rapamycin and serum starvation was used as a positive control for increased autophagic flux and 20 nM bafilomycin 1A were used as autophagy blocker. Red line indicates the level of DMSO control and green hatched line indicates the level of rapamycin control. (C) Fluorescence signal after 3 h oxidative stress ($FeCl_2$+H_2O_2) +/- PREP ligand. 10 µM concentration for test compounds were used. Red line indicates the level of stress+DMSO control and green line for non-stressed cells. *, p<0.05; **, p<0.01; *, p<0.001 1-Way ANOVA with Dunnett's post hoc test (compared to DMSO control). Results are presented as mean+SEM.

Cell Viability

The impact of selected thiazole compounds on cell viability was tested in human kidney (HEK-293) and neuronal (SH-SY5Y) cell cultures and in mouse primary neurons. Effective dose that was used in the cellular assay (10 µM) did not show toxicity with any of the compounds but particularly **HUP-46** showed toxicity in high dose (100 µM; Figure S52). This could be due to the possible off-targets with higher doses or then due the excessive PP2A activation (see Figure 2). However, no toxicity was seen in the PD mouse model during 4-week administration. Interestingly, **7** that lacks CN group in the P1 position showed less toxicity in cells than **HUP-46**.

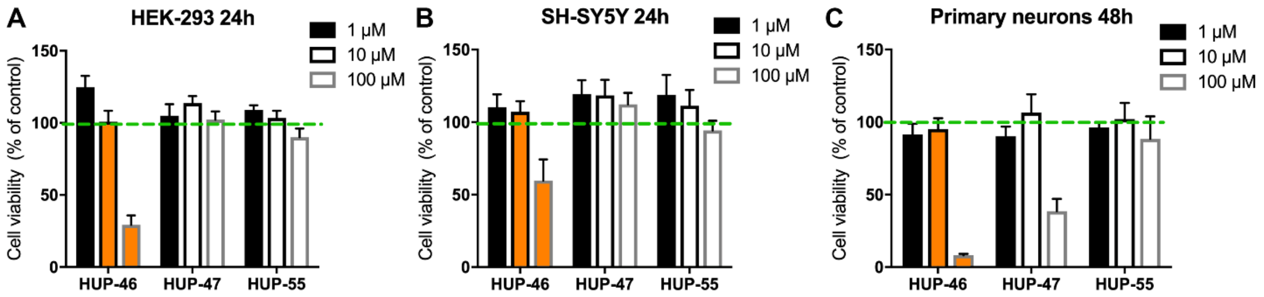

Figure S52. The impact of **HUP-46** and **7** on cell viability of (A) HEK-293 and (B) SH-SY5Y cells after 24 h incubation, and (C) on mouse primary neurons after 48 h incubation on MTT assay. HUP-55 from oxazole-based PREP ligands served as a control. Data is presented as mean +/- SEM.

Close relative enzyme specificity assay

The effect of **HUP-46** on close-relative enzymes of PREP, FAP, DPP4, DPP9 and DPP2 was tested on specific enzyme activity assays. No inhibition on other peptidases than PREP was seen with 1 and 10 µM concentrations (Figure S53).

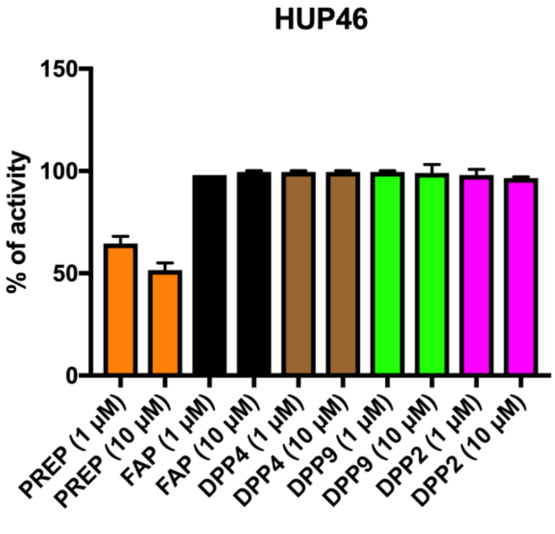

Figure S53. The impact of **HUP-46** on enzyme activities of PREP, fibroblast activating protein (FAP), dipeptidyl peptidase 4, 9 and 2. No inhibition was seen except in PREP. Data is presented as mean +/- SEM.

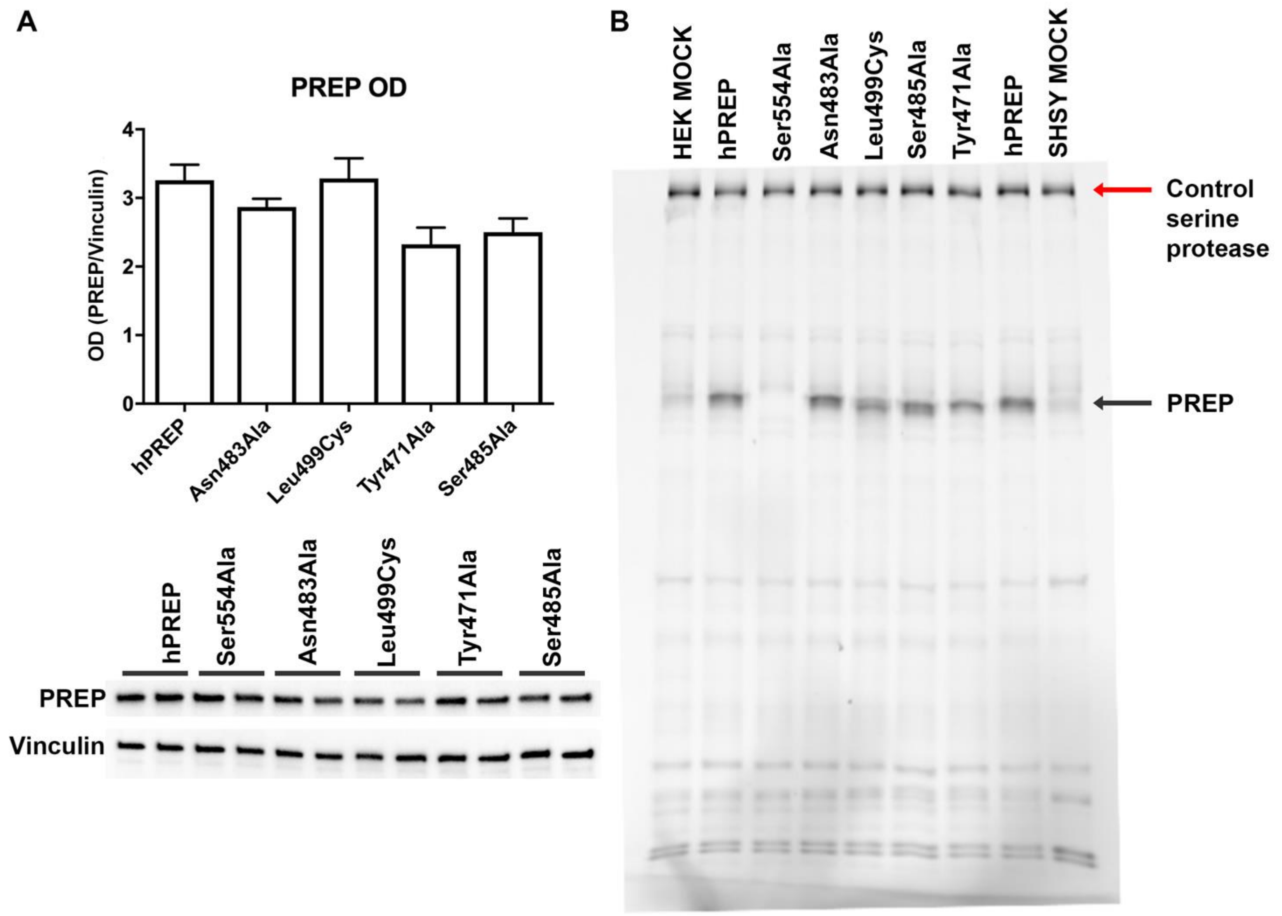

Figure S54. The expression levels of PREP in PREP-KO HEK-293 cells after hPREP and mutant hPREP construct transfections (A). The whole ABPP membrane showing PREP band and a control serine protease that was used as a loading control (B). Serine 554 Alanine PREP mutant was used as a control for PREP ABPP, and tested also for expression in A. Data are presented as means ± SEM.

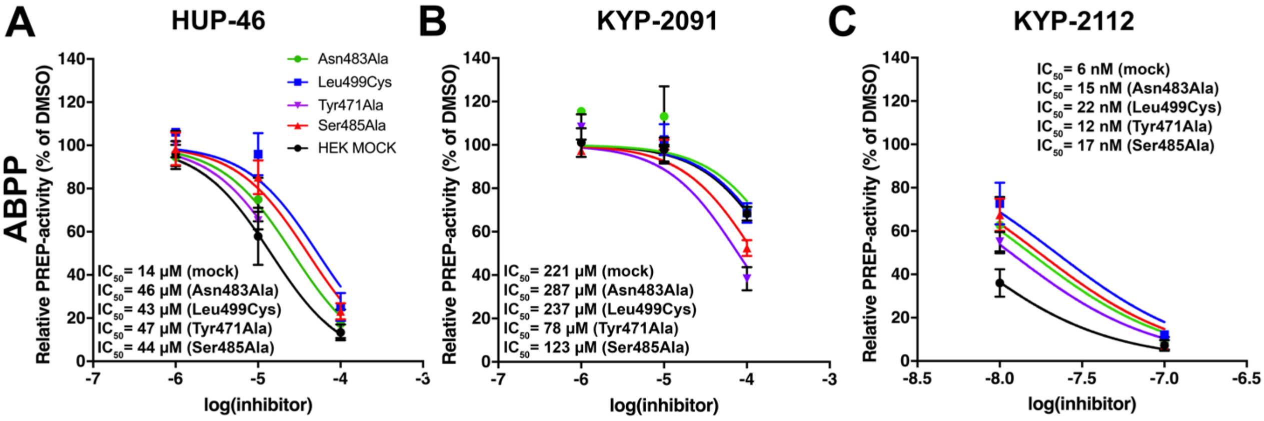

Figure S55. The inhibition of proteolytic activity of PREP by **HUP-46**, KYP-2091 and KYP-2112 was tested in the activity-based protein profiling assay (ABPP) assay in mutant transfected HEK-293 cells (A-C; mock is cells transfected with wt hPREP). IC_{50} values are presented in the figures, and no statistical differences were seen between the mutants. Data are presented as means ± SD.

ITC

ITC was used to measure K_d-values for KYP-2112 and KYP-2091 against wt PREP and Asn483Ala or Leu499Cys point mutated PREP. Interestingly, also the stoichiometry shown in Table S2 was different for KYP-2091 in mutants compared to wt protein. Stoichiometry of 2.2 in wt might indicate that KYP-2091 binds to another binding site as well. For KYP-2112 the number of binding sites did not change between mutants. Similar effects were also observed in the change of enthalpy (Table S3) as KYP-2091 had lower binding enthalpy when binding to mutants compared to wild type, but no such difference was seen with KYP-2112.

Table S2. Number of binding sites for PREP and two mutants for KYP-2112 and KYP-2091. Number of n-sites changes for KYP-2091 when using mutants instead of wild type PREP but for KYP-2112 similar effect was not seen (*, $p<0.05$ compared to wt hPREP in Kruskal-Wallis test combined with a Dunn's multiple comparison's test).

Protein	n-sites (n)	
	KYP-2112	KYP-2091
wt hPREP	1.5 ± 0.4	2.3 ± 0.2
Asn483Ala	1.8 ± 0.4	1.3 ± 0.02 *
Leu499Cys	1.7 ± 0.3	1.5 ± 0.3 *

Table S3. ΔH for binding to PREP and two mutants for KYP-2112 and KYP-2091. KYP-2091 has a lower ΔH value when binding to mutated PREP compared to wild type PREP, but no such effect was present with KYP-2112 (*, $p<0.05$; **, $p<0.01$ compared to wt hPREP in Kruskal-Wallis test combined with a Dunn's multiple comparison's test).

Protein	ΔH (kcal/mol)	
	KYP-2112	KYP-2091
wt hPREP	-14.5 ± 6.5	-2.9 ± 0.5
Asn483Ala	-12.9 ± 1.6	-4.9 ± 1.2**
Leu499Cys	-12.6 ± 0.6	-4.5 ± 0.8*

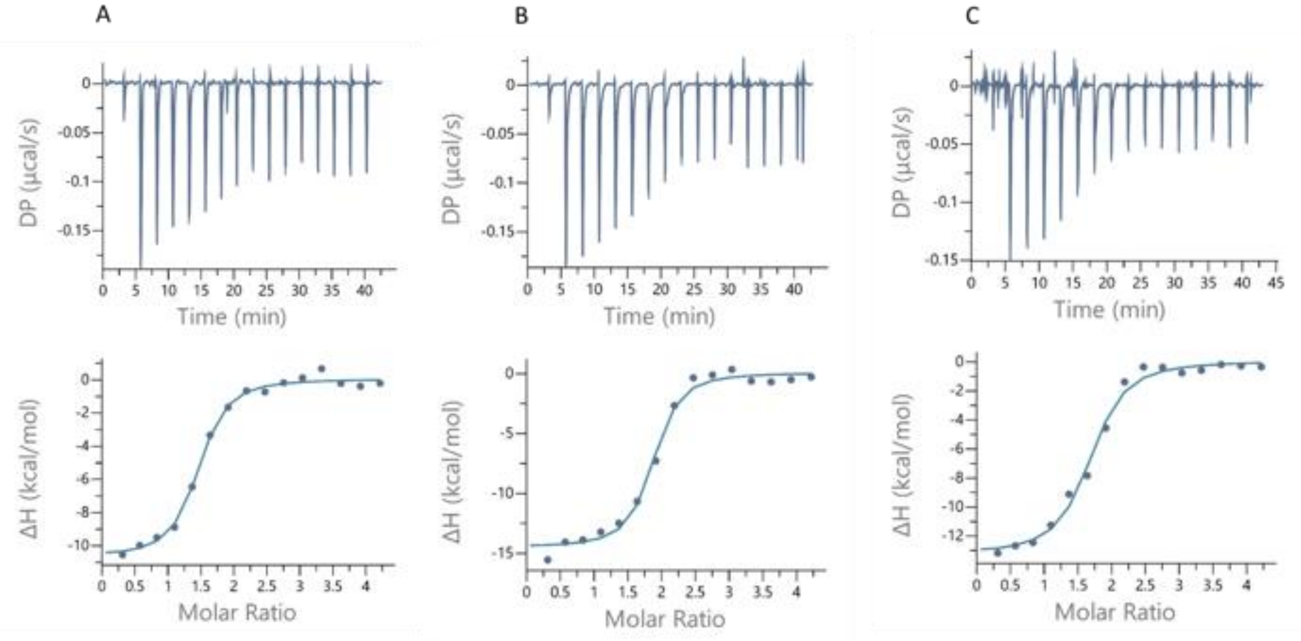

Figure S56. Binding curves for KYP-2112. The binding curves for wild type (A), Asn483Ala (B) and Leu499Cys (C) PREP are similar even though the n-value and ΔH are slightly different in both of the mutants compared to wild type. Used protein concentration was 2 µM and used KYP-2112 concentration was 43 µM

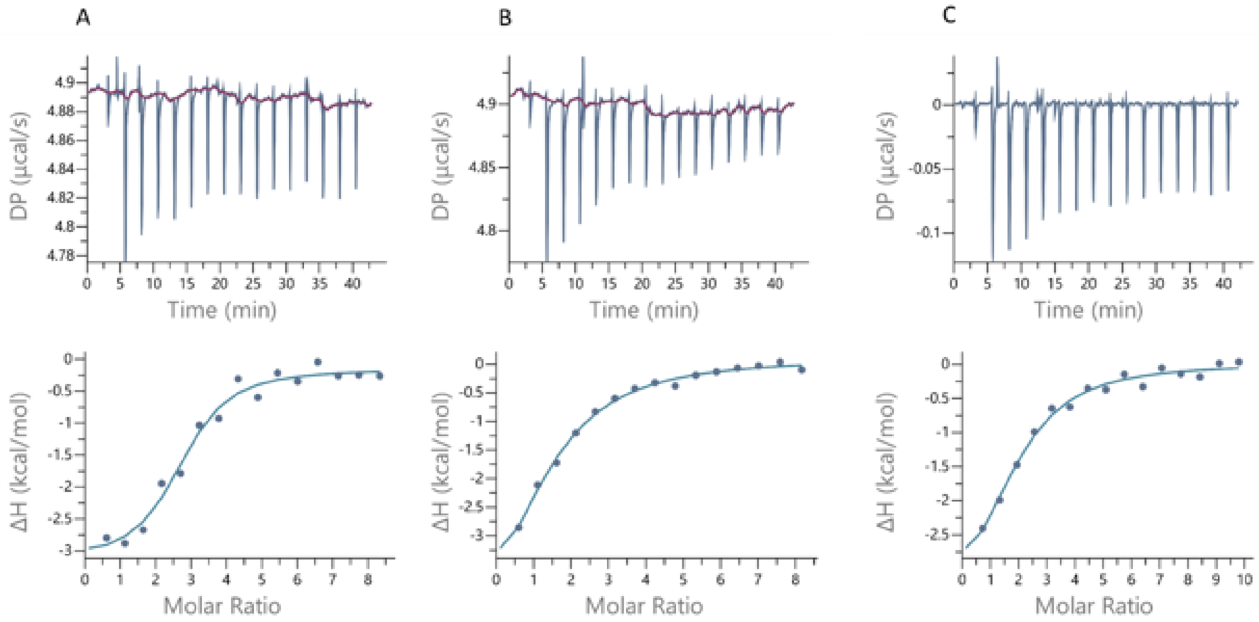

Figure S57. The binding curves for KYP-2091 against wild type (A), Asn483Ala (B) and Leu499Cys (C) PREP. Binding curves demonstrate the difference in binding between the wildtype and two mutants, n-value is higher in wild type and K_d-value is lower compared to mutants. 85 µM concentration of KYP-2091 and 2 µM concentration of PREP was used for wild type, for both mutants 3 µM of protein were used and 125 µM of KYP-2091 were used for Asn483Ala and 150 µM for Leu499Cys.

The signature binding plots were also evaluated and are presented in figures below. Blue bar indicates difference in Gibbs free energy, which is related to the affinity of the molecule. Green bar, enthalpy, indicates the change in energy content of the bonds broken and created, e.g. caused by hydrogen bonds and van der Waals forces. Red bar shows the change in entropy which is mainly due to hydrophobic interactions. As seen in Figure S58 there is no change in the signature plots for KYP-2112 and the binding is mainly enthalpy-driven with a relatively small unfavorable entropic contribution, as the green bar is highly negative and the red bar is slightly positive. However, KYP-2091 binds completely differently to the mutants compared to wildtype (Figure S59). When KYP-2091 binds to wildtype PREP, the interaction seems mainly driven by entropy, with a smaller enthalpic contribution. For the mutants, the enthalpic contribution to the binding seems relatively more important, also taking into account the lower affinity compared to the wild type PREP.

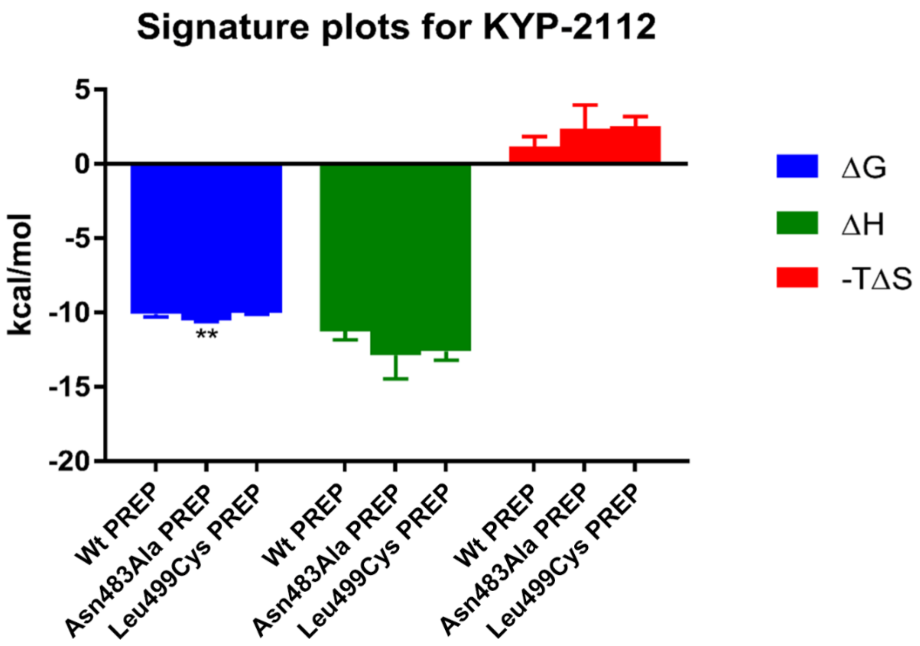

Figure S58. Signature plots comparison between mutants for KYP-2112. The signature binding plots for wild type (A), Asn483Ala (B) and Leu499Cys (C) PREP are similar indicating that mutations do not change the binding of KYP-2112. ** p<0.005, 1-way ANOVA with Dunnett's multiple comparison. Data are presented as means ± SD.

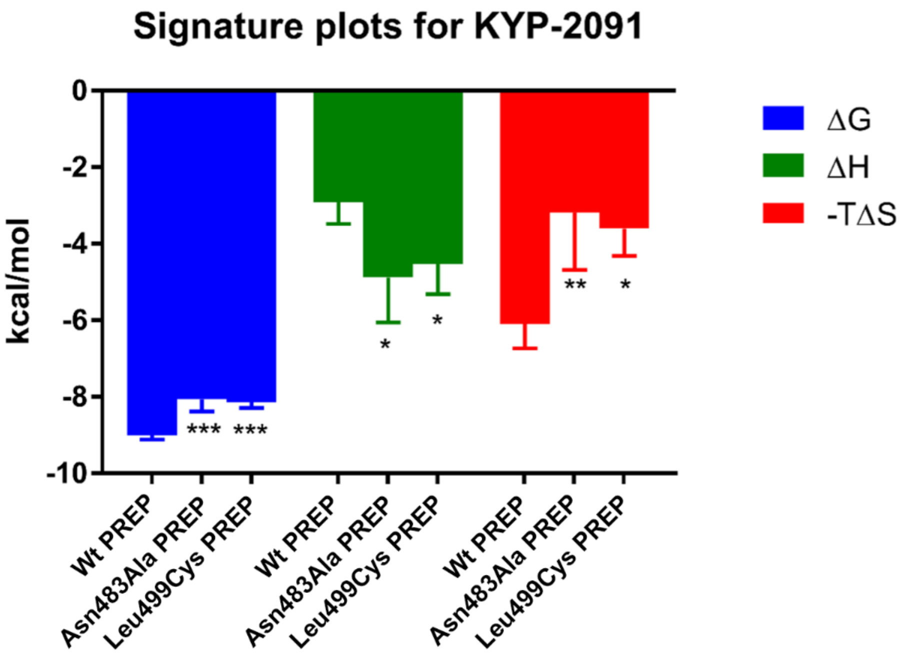

Figure S59. Signature plots comparison between mutants for KYP-2091. The signature binding plots for the Asn483Ala and Leu499Cys mutants are significantly different from the wild type, indicating that mutations changes the binding of KYP-2091. * p<0.05, ** p<0.005, *** p<0.001, 1-way ANOVA with Dunnett's multiple comparison. Data are presented as means ± SD.

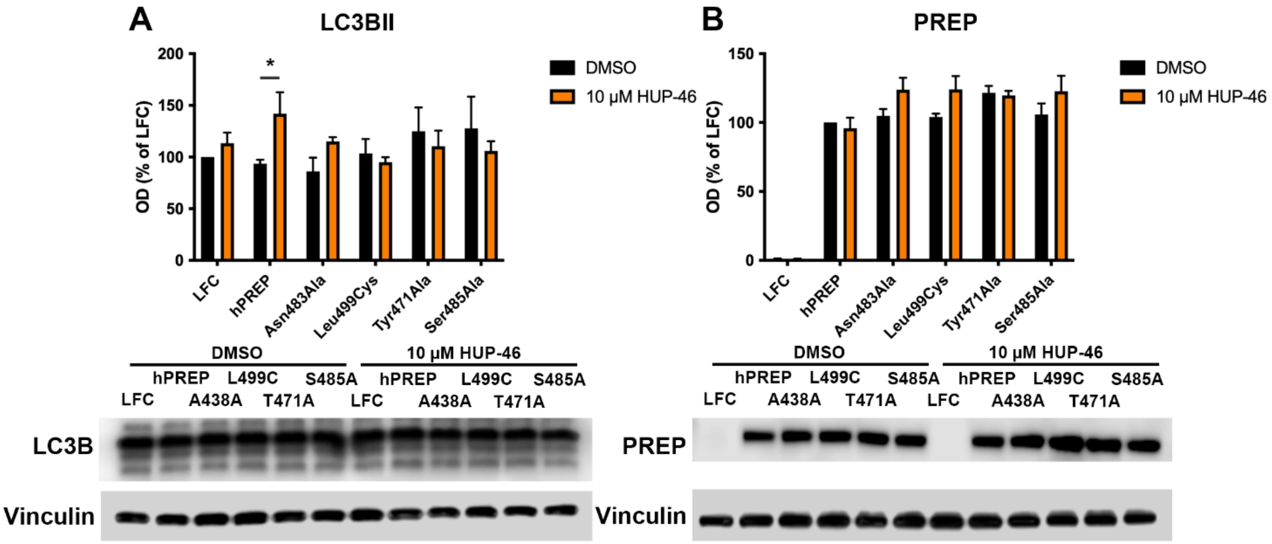

Figure S60. The effect of mutant PREP constructs on LC3BII and the levels of total PREP. Similar to pPP2A, HUP-46 elevated LC3BII levels only in the cells transfected with hPREP (A). Other changes caused by PREP mutants were not significant. Total PREP levels between the constructs were equal (B). *, $p<0.05$, 2-way ANOVA with Uncorrected Fisher's LSD. Data are presented as mean ± SEM.

Brain Penetration

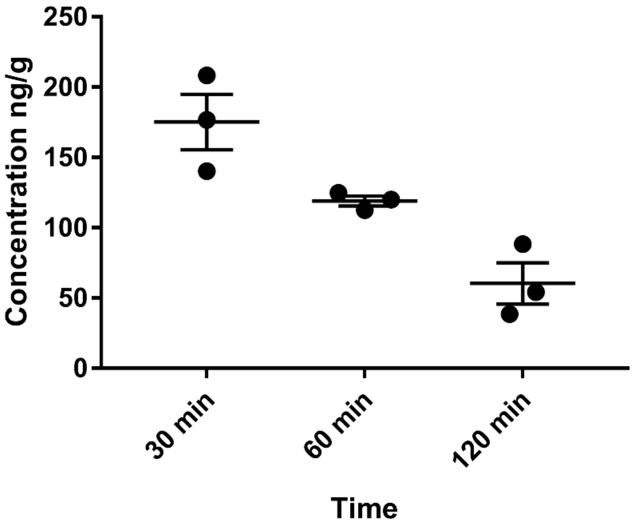

Figure S61. Brain concentrations of **HUP-46** after 10 mg/kg i.p. injection measured by MS-LC.

References

1. Haffner CD, Diaz CJ, Miller AB, et al. Pyrrolidinyl pyridone and pyrazinone analogues as potent inhibitors of prolyl oligopeptidase (POP). *Bioorg Med Chem Lett*. 2008;18(15):4360-4363. doi:10.1016/j.bmcl.2008.06.067
2. Fülöp V, Böcskei Z, Polgár L. Prolyl oligopeptidase: an unusual beta-propeller domain regulates proteolysis. *Cell*. 1998;94(2):161-170. doi:10.1016/s0092-8674(00)81416-6
3. Kaszuba K, Róg T, Danne R, et al. Molecular dynamics, crystallography and mutagenesis studies on the substrate gating mechanism of prolyl oligopeptidase. *Biochimie*. 2012;94(6):1398-1411. doi:10.1016/j.biochi.2012.03.012
4. Schrödinger Release 2022-3: Maestro, Protein Preparation Wizard, Prime, Epik, SiteMap, LigPrep, Glide, Induced Fit Docking protocol, Desmond Molecular Dynamics System; Schrödinger, LLC, New York, NY, 2020.
5. Olsson MH, Søndergaard CR, Rostkowski M, Jensen JH. PROPKA3: Consistent Treatment of Internal and Surface Residues in Empirical pKa Predictions. *J Chem Theory Comput*. 2011;7(2):525-537. doi:10.1021/ct100578z
6. Savolainen MH, Yan X, Myöhänen TT, Huttunen HJ. Prolyl oligopeptidase enhances α-synuclein dimerization via direct protein-protein interaction. *J Biol Chem*. 2015;290(8):5117-5126. doi:10.1074/jbc.M114.592931
7. Svarcbahs R, Jäntti M, Kilpeläinen T, et al. Prolyl oligopeptidase inhibition activates autophagy via protein phosphatase 2A. *Pharmacol Res*. 2020;151:104558. doi:10.1016/j.phrs.2019.104558
8. Svarcbahs R, Julku UH, Norrbacka S, Myöhänen TT. Removal of prolyl oligopeptidase reduces alpha-synuclein toxicity in cells and in vivo. *Sci Rep*. 2018;8(1):1552. doi:10.1038/s41598-018-19823-y
9. Myöhänen TT, Hannula MJ, Van Elzen R, et al. A prolyl oligopeptidase inhibitor, KYP-2047, reduces α-synuclein protein levels and aggregates in cellular and animal models of Parkinson's disease. *Br J Pharmacol*. 2012;166(3):1097-1113. doi:10.1111/j.1476-5381.2012.01846.x
10. Van der Veken P, Fülöp V, Rea D, et al. P2-substituted N-acylprolylpyrrolidine inhibitors of prolyl oligopeptidase: biochemical evaluation, binding mode determination, and assessment in a cellular model of synucleinopathy. *J Med Chem*. 2012;55(22):9856-9867. doi:10.1021/jm301060g
11. De Meester I, Vanhoof G, Lambeir AM, Scharpé S. Use of immobilized adenosine deaminase (EC 3.5.4.4) for the rapid purification of native human CD26/dipeptidyl peptidase IV (EC 3.4.14.5). *J Immunol Methods*. 1996;189(1):99-105. doi:10.1016/0022-1759(95)00239-1
12. Van Elzen R, Schoenmakers E, Brandt I, Van Der Veken P, Lambeir AM. Ligand-induced conformational changes in prolyl oligopeptidase: a kinetic approach. *Protein Eng Des Sel*. 2017;30(3):217-224. doi:10.1093/protein/gzw079
